# Effect of antenatal corticosteroid administration-to-birth interval on maternal and newborn outcomes: a systematic review

**DOI:** 10.1016/j.eclinm.2023.101916

**Published:** 2023-03-24

**Authors:** Annie R.A. McDougall, Lily Aboud, Tina Lavin, Jenny Cao, Gabrielle Dore, Jen Ramson, Olufemi T. Oladapo, Joshua P. Vogel

**Affiliations:** a Maternal, Child and Adolescent Health Program, Burnet Institute, Melbourne, Australia; b Department of Sexual and Reproductive Health and Research, UNDP/UNFPA/UNICEF/WHO/World Bank Special Programme of Research, Development and Research Training in Human Reproduction (HRP), World Health Organization, Geneva, Switzerland; c Royal Brisbane and Women's Hospital, Brisbane, Australia; d School of Public Health and Preventive Medicine, Monash University, Melbourne, Australia

**Keywords:** Betamethasone, Dexamethasone, Glucocorticoids, Evidence-synthesis, Neonatal mortality

## Abstract

**Background:**

Antenatal corticosteroids (ACS) are highly effective at improving outcomes for preterm newborns. Evidence suggests the benefits of ACS may vary with the time interval between administration-to-birth. However, the optimal ACS administration-to-birth interval is not yet known. In this systematic review, we synthesised available evidence on the relationship between ACS administration-to-birth interval and maternal and newborn outcomes.

**Methods:**

This review was registered with PROSPERO (CRD42021253379). We searched Medline, Embase, CINAHL, Cochrane Library, Global Index Medicus on 11 Nov 2022 with no date or language restrictions. Randomised and non-randomised studies of pregnant women receiving ACS for preterm birth where maternal and newborn outcomes were reported for different administration-to-birth intervals were eligible. Eligibility screening, data extraction and risk of bias assessment were performed by two authors independently. Fetal and neonatal outcomes included perinatal and neonatal mortality, preterm birth-related morbidity outcomes and mean birthweight. Maternal outcomes included chorioamnionitis, maternal mortality, endometritis, and maternal intensive care unit admission.

**Findings:**

Ten trials (4592 women; 5018 neonates), 45 cohort studies (at least 22,992 women; 30,974 neonates) and two case–control studies (355 women; 360 neonates) met the eligibility criteria. Across studies, 37 different time interval combinations were identified. There was considerable heterogeneity in included administration-to-birth intervals and populations. The odds of neonatal mortality, respiratory distress syndrome and intraventricular haemorrhage were associated with the ACS administration-to-birth interval. However, the interval associated with the greatest improvements in newborn outcomes was not consistent across studies. No reliable data were available for maternal outcomes, though odds of chorioamnionitis might be associated with longer intervals.

**Intepretation:**

An optimal ACS administration-to-birth interval likely exists, however variations in study design limit identification of this interval from available evidence. Future research should consider advanced analysis techniques such as individual patient data meta-analysis to identify which ACS administration-to-birth intervals are most beneficial, and how these benefits can be optimised for women and newborns.

**Funding:**

This study was conducted with funding support from the UNDP-UNFPA-UNICEF-WHO-World Bank Special Programme of Research, Development and Research Training in Human Reproduction (HRP), Department of Sexual and Reproductive Health and Research (SRH), a co-sponsored programme executed by the 10.13039/100004423World Health Organization.

**Research in context**

**Evidence before this study**

Evidence from animal studies suggest that the time interval between administration of antenatal corticosteroids (ACS) and birth is associated with newborn outcomes. The 2006 iteration of the Cochrane review on trials of ACS efficacy included a subgroup analysis that explored the effect of administration to delivery interval (Roberts et al., 2006). This analysis included data from five trials and four time intervals: <24 h, <48 h, 1–7 days and >7 days after administration, identifying few trials per interval. The large body of evidence from observational studies had not previously been included in any systematic reviews.

**Added value of this study**

To the best of our knowledge, this is the first systematic review that specifically investigates the evidence from both clinical trials and observational studies for an association between the ACS administration-to-birth interval and maternal, fetal and newborn outcomes. We found significant heterogeneity between the time interval reported, and the populations included across studies, making direct statistical comparisons difficult. Narratively, we present evidence that the odds of neonatal mortality, respiratory distress syndrome and intraventricular haemorrhage are likely associated with the administration-to-birth interval, but optimal time intervals identified for newborn outcomes were not consistent across studies, or for different outcomes.

**Implications of all the available evidence**

This review suggests that there is an association between the ACS administration-to-birth interval and maximising benefits for preterm newborns. An inherent challenge for this research question is that the administration-to-birth interval can be difficult to predict or modify for individual women. Further research on this topic should consider the use of advanced statistical modelling techniques and individual patient data meta-analysis to identify which ACS administration-to-birth intervals are most beneficial.

## Introduction

Every year, 15 million babies are born preterm (<37 completed weeks’ gestation).^1^ Preterm birth is the leading cause of death in children under 5, and approximately 35% of neonatal deaths in the first 28 days of life are caused by preterm birth complications.^2^^,^^3^ Preterm newborns are at increased risk of developing respiratory distress syndrome, intraventricular haemorrhage, necrotising enterocolitis and sepsis, as well as longer-term morbidities such as chronic lung disease and neurological disabilities.^4^

Antenatal corticosteroids (ACS) are an effective intervention for improving outcomes for neonates born to women at risk of early preterm birth. They confer benefits by crossing the placenta and accelerating structural maturation of fetal lung tissue and other organs.^5^^,^^6^ The 2020 update of the Cochrane review on ACS efficacy found that ACS use significantly reduces the risk of moderate/severe respiratory distress, perinatal death and neonatal death, and probably reduces the risk of intraventricular haemorrhage (IVH) and developmental delay in childhood.^7^ WHO currently recommends that ACS should be administered to women between 24 and 34 weeks’ gestation who are at risk of imminent preterm birth, provided that certain criteria related to a minimum level of maternal and preterm newborn care can be met.^8^

Although the benefits of ACS in early preterm birth are established, questions remain as to the optimal time interval between initiation of ACS and birth. Preclinical animal studies suggest that longer time intervals favour fetal lung maturation–in fetal sheep given a direct injection of betamethasone, increased lung compliance was evident 15 h later.^9^ When maternal sheep were treated with betamethasone, early signs of lung maturation in the fetus–such as increased ventilator efficiency index, increased airspace, and decreased alveolar wall volume–were observed after 2 days.^10^ By 7 days, additional signs of lung maturation were present, such as increased compliance, markers of surfactant production and alveolar wall thinning.^10^ These improvements in fetal sheep lung function were observed up to 21 days after a single dose of maternal betamethasone.^11^ A 2020 sheep model study specifically examined the association between administration-to-birth interval and lung maturation, finding higher lung gas volume and ventilation efficiency index at 2, 5, 7 and 10 days after betamethasone administration with peak improvement at 5 and 7 days, though markers of increased surfactant production were increased at the 7-day interval only.^12^ Human trials provide some additional evidence on the role of ACS administration-to-birth interval. For example, the 2020 WHO ACTION-I trial reported that longer intervals were associated with better newborn outcomes for early preterm newborns, regardless of gestational age at time of administration.^13^

Available evidence suggests that the administration-to-birth interval probably has important effects on the degree of fetal lung maturation and consequently preterm newborn outcomes, though the ideal (or optimal) interval is unclear. Many factors can complicate the effects of ACS, such as the gestational age at administration, as well as additional fetal development occurring in women that remained undelivered. Evidence on this question is important to guide clinicians and other stakeholders on identifying how benefits from ACS might be optimised, and possible harms minimised. Therefore, the aim of this systematic review was to assess the relationship between ACS administration-to-birth intervals and maternal and newborn outcomes and to identify the “optimal interval” to achieve greatest benefit.

## Methods

The systematic review was conducted in accordance with Cochrane Handbook guidance (Version 5.1).^14^ The review protocol was registered on PROSPERO (CRD42021253379) and reported according to the PRISMA checklist (Appendix S1). As a systematic review of published studies, ethical approval was not required.

### Eligibility criteria

Eligible studies were primary research studies of women with a singleton or multiple pregnancy who were administered ACS in the context of anticipated preterm birth, whether due to spontaneous preterm labour, preterm prelabour rupture of the membranes or provider-initiated preterm birth. Both randomised and non-randomised designs were eligible, including observational designs (i.e. cohort, cross-sectional and case–control studies), and non-randomised interventional studies. Studies were eligible regardless of ACS type, dose, or regimen. Studies that reported including women who received multiple (repeat or rescue) courses of ACS were excluded, however if studies presented women who received single or multiple courses separately, studies were included and only data on women who received single courses were extracted. We only included studies that reported outcome data for two or more different ACS administration-to-birth intervals. Included studies could have a comparison group of any time interval or no ACS. There were no restrictions in terms of language, date of publication or setting. Translation services were used for studies reported in a language other than English. Studies pertaining to ACS administration to term fetuses (≥37 weeks’ gestational age) were excluded, as were animal studies, case reports/series, letters, commentaries, conference abstracts, protocol papers and systematic reviews.

### Search strategy, study selection, data extraction and risk of bias assessment

We developed a search strategy with assistance from an information specialist. Five databases were searched (Medline, Embase, CINAHL, Cochrane Library, Global Index Medicus, see Appendix S2 for search strategy) on 11 November 2022. Citations were collated in Endnote and screened in duplicate by two review authors using Covidence.^15^ Full-text articles were recovered for potentially eligible studies which were screened by two authors independently. Any disagreements during title/abstract or full-text screening were resolved through discussion or consulting a third author.

Review outcomes were based on those used in WHO's recommendations on interventions to improve preterm birth outcomes.^16^ Newborn outcomes of interest were perinatal mortality, neonatal mortality, moderate or severe respiratory distress syndrome (RDS), chronic lung disease (bronchopulmonary dysplasia), IVH (grade 3 or 4), sepsis, necrotising enterocolitis (NEC), retinopathy of prematurity (ROP), patent ductus arteriosis (PDA), neonatal intensive care unit (NICU) admission, neonatal hypoglycaemia, and mean birth weight. Maternal outcomes of interest were maternal mortality, chorioamnionitis, endometritis, and intensive care unit admission. Outcomes were as defined by study authors.

Data extraction was performed using a pre-designed data extraction spreadsheet that was pilot tested on five eligible studies and revised. For each included study, data were extracted on study design (individually randomised trial, cluster-randomised trial, cohort study, cross-sectional study or case–control study), country, publication year, mean gestational age, population characteristics, plurality, sample size (maternal or newborn participants), type of ACS used (e.g. betamethasone, dexamethasone) and mean gestational age at birth. The ACS administration-to-birth time intervals used in the study were extracted, as well as how time of ACS administration was defined (from first dose, from first course, from last dose or other). We then extracted any reported data for all review outcomes for each of the ACS administration-to-birth time intervals used. In accordance with Cochrane handbook guidance, the quality of included studies were assessed using the Cochrane Risk of Bias 2 (RoB 2) tool for trials and the Risk Of Bias In Non-randomised Studies of Interventions (ROBINS-I) tool for non-randomised studies.^14^ Based on these tools, an overall risk of bias judgement was determined for each study. Data extraction and quality assessment were performed independently by two authors, with discrepancies resolved through discussion or consultation with a third reviewer.

### Data analysis

Characteristics of studies, populations, prevalence of outcomes and measures of association (relative risk [RR] or odds ratios [OR], including their 95% confidence intervals [CI]) were reported descriptively. For studies where outcome prevalence data were reported (five or more events), but not measures of association, the crude OR (95% CI) was calculated by systematic review authors, compared to the control group designated by original study authors. For each included study, we used the available data and author's conclusions to identify what (if any) ACS administration-to-birth interval was associated with improved health outcomes. That is, the administration-to-birth interval/s where the OR (or RR) was significantly lower than the comparator and was thus defined as an “optimal interval” by the study authors, for a given outcome.

While the review protocol pre-specified using meta-analysis to pool data for different time intervals, the available data were clinically heterogenous. Specifically, the study populations varied on several factors including gestational age range, inclusion or exclusion of multiple births, and others. Gestational age of administration and number of gestations are known confounders of studies examining ACS and are often reported separately in meta-analysis.^7^ In addition, studies varied in terms of the ACS administration-to-birth intervals used. The variation was so great that pooling of intervals without having overlapping intervals across studies would require so many individual intervals to be pooled that significant information would be wasted. In light of these differences, we considered it inappropriate to pool data. However, for those studies in which the RR or OR (95% CI) were available using a comparator of no ACS (such as a placebo arm in randomised trials, or the “no ACS” group in observational studies), we used descriptive forest plots without meta-analysis for visual comparison of individual study findings for the reported time intervals. This allowed the largest number of studies to be examined and demonstrated which time intervals conferred benefit (or harm) or had no effect.

### Role of funding source

This review was commissioned by the UNDP-UNFPA-UNICEF-WHO-World Bank Special Programme of HRP, SRH to inform WHO recommendations for antenatal care. Authors TL and OTO are staff of WHO, had access to the dataset and contributed to the decision to submit for publication.

## Results

**Fig. 1:**
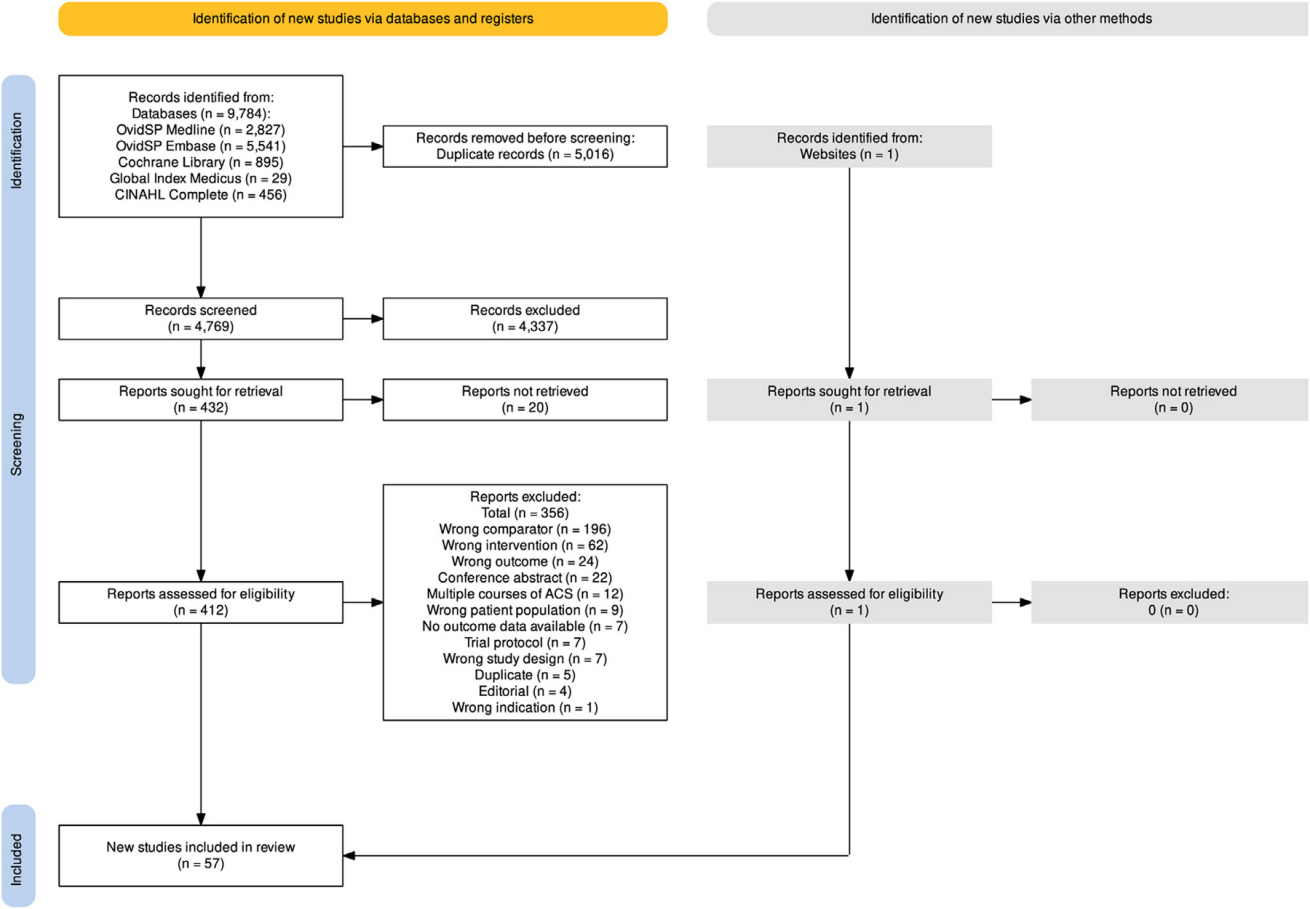
**PRISMA flowchart ofincluded studies.**

The search identified a total of 9784 records, after abstract screening 412 articles were reviewed in full text. A total of 57 articles were eligible and included in the review (Fig. 1).

### Characteristics of included studies

The review identified 10 randomised controlled trials (Table 1), 45 cohort studies and two case–control studies (Table 2). In total, 37 different combinations of ACS administration-to-birth intervals were used across these studies (Table S1). Data from the 10 randomised controlled trials has been presented separately from the observational studies, however it is acknowledged that the women in these trials were not randomised to receive ACS at different time intervals and as such, the data more accurately reflects cohort studies nested within a trial.

**Table 1:** Characteristics of included randomised controlled trials.

Study	Country	Country income level	Population	GA (weeks)	Sample size	Intervention groups	Comparisons	Review outcomes reported
Anonymous 1981^17^	USA	High income	Women with singleton or multiple pregnancy at high risk of preterm labour	26–37	696 women 757 neonates	Dexamethasone No ACS	<24 h, 24 h to 7 days, >7 days from 1st dose	RDS
Block 1977^18^	USA	High income	Singleton neonates	NS	128 women 128 neonates	Betamethasone No ACS	≤24 h, >24 h from 1st dose	RDS
Dexiprom 1999^19^	South Africa	Upper middle income	Singleton and multiple neonates of women with PPROM	28–34	204 women 208 neonates	Dexamethasone No ACS	<24 h, >24 h from 1st dose	Perinatal mortality, neonatal mortality, fetal mortality, RDS, Chorioamnionitis
Gamsu 1989^20^	UK	High income	Women with singleton or multiple pregnancy in spontaneous preterm labour or with complications requiring birth	<34	251 women 262 neonates	Betamethasone No ACS	1–6 days, 1–14 days, 1–21 days from 1st dose	RDS
Kari 1994^21^	Finland	High income	Women with singleton or multiple pregnancies and threatened preterm birth with intact fetal membranes and no CAM, insulin treated diabetes or fetal anomaly	24–<32	157 women 189 neonates	Dexamethasone No ACS	<24 h, 1–14 days from 1st dose	Neonatal mortality, RDS, IVH
Liggins 1972^22^	New Zealand	High income	Women with singleton or multiple pregnancy and threatened or planned (due to obstetric complications) preterm labour	24–36	213 women 226 neonates	Betamethasone No ACS	<24 h, 24–48 h, 2–7 days, >7 days from 1st dose	RDS
Luerti 1987^23^^a^	Italy	High income	Women with singleton or multiple pregnancies and threatened or planned (due to obstetric complications) preterm labour	27–34	152 women 169 neonates	Betamethasone	<2 days, 2–7 days, >7 days from ACS (dose/course not specified)	RDS
Schutte 1980^24^	Netherlands	High income	Women with singleton or multiple pregnancies with threatened preterm labour	26–32	79 women 95 neonates	Betamethasone No ACS	<12 h, 12 h to 7 days, 8–21 days, >21 days from administration of 1st dose	RDS
Teramo 1980^25^	Finland	High income	Women with singleton or multiple pregnancies	28–35	74 women 80 neonates	Betamethasone No ACS	1–7 days, >7 days from administration of 1st dose	RDS
WHO 2022^26^	Bangladesh, India, Kenya, Nigeria, Pakistan	Lower middle income	Women with singleton or multiple pregnancy and confirmed live fetus	26–34	2638 women 2904 neonates	Dexamethasone No ACS	<6 h, >6–12 h, >12–24 h, >24 h to 7 days, >7 days from 1st dose 0–28 days (continuous variable)	Perinatal mortality; neonatal mortality

Notes: ACS = antenatal corticosteroid; GA = gestational age at trial entry; IVH = intraventricular haemorrhage; RDS = respiratory distress syndrome.

a Luerti et al., 1987 trial compared outcomes between betamethasone or ambroxol treated women. Only data from subgroup analysis of RDS in betamethasone group for different administration-to-birth intervals included in this review.

**Table 2:** Characteristics of included observational studies.

Study	Study design	Country	Country income level	Population	GA (weeks)	Sample size	Intervention	Time intervals	Review outcomes reported
Arulalan 2022^27^	Bidirectional cohort	India	Lower middle income	Pregnant women with twin gestations who completed a single course of ACS	28–34	268 women.536 neonates	Betamethasone or dexamethasone	≤14 days, >14 days from administration of last dose	Neonatal mortality; RDS
Asl 2005^28^	Prospective cohort	Iran	Upper middle income	Pregnant women at GA of 30–36 weeks, had regular uterine contractions with cervix dilation of at least 2 cm and cephalic presentation.	30–36	Number of women not stated 170 neonates	Dexamethasone No ACS	No ACS, <24 h from administration of 1st dose	RDS
Barrett 1982^29^	Prospective cohort	USA	High income	Women with singleton or multiple pregnancy with ruptured membranes and no CAM, fetal distress or abrupt placentae	24–36	89 women 93 neonates	Unspecified ACS No ACS	Hours: <24, 24 to 47, 48–71, 72–95, 96–143, >144 from 1^st^dose	Neonatal mortality; sepsis; CAM
Battarbee 2020^30^	Prospective cohort (secondary analysis)	USA	High income	Singleton neonates	20–36^6^	2259 women 2259 neonates	Unspecified ACS	<2 days, 2–<7 days, 7–<14 days and 14 days from 1st dose	Neonatal mortality; RDS; BPD; IVH; NEC; mean BW; CAM
Biedermann 2022^31^	Prospective cohort	Germany	High income	Neonates with a birthweight <1500 g and gestational age <34 weeks, treated in the NICU	<34	239 women 239 neonates	Betamethasone No ACS	<48 h, 2–7 days, >7 days from administration of 1st dose	Neonatal mortality, BPD, sepsis, NEC
Chawla 2010^32^	Retrospective cohort	USA	High income	Singleton neonates with BW 401–1000 g and without congenital anomalies	≤28	169 women 169 neonates	Unspecified ACS No ACS	<24 h, 24 h to 7 days, >7 days from 1st dose	Neonatal mortality; BPD; IVH; sepsis; NEC; mean BW; CAM
Di Pasquo 2020^33^	Retrospective cohort	Italy	High income	Singleton neonates without congenital anomalies, hypoxic ischemic encephalopathy or sepsis	24^0^–36^7^	99 women 99 neonates	Betamethasone	24 h to 7 days, <24 h or >7 days from ACS (does not specify whether dose or course)	Hypoglycaemia
Dzidek 2020^34^	Prospective cohort	Poland	High income	Women with singleton or multiple pregnancy and threatened preterm birth, PROM, medical indications for birth or cervical incompetence	24–34	459 women 530 neonates	Betamethasone or dexamethasone	≤7 days, >7 days from ACS (does not specify whether dose or course)	Mean BW
Ferguson 2009^35^	Retrospective cohort	Canada	High income	Women with singleton pregnancy and severe hypertension of pregnancy	26–34	172 women 172 neonates	Betamethasone or dexamethasone	≤48 h, >48 h from 1^st^dose	RDS; sepsis; mean BW
Fortmann 2022^36^	Prospective cohort	Germany	High income	VLBW neonates born before 30 weeks gestational age	23–30	Number of women not stated 672 neonates	Betamethasone No ACS	No ACS, <24 h (1 dose), 24 h–7 days (2 doses), >7 days (2 doses) from administration of 1st dose	Neonatal mortality, BPD, IVH, sepsis,
Fuller 2017^37^	Retrospective cohort	USA	High income	Singleton Neonates at 23^0^–33^6^	23^0^–33^6^	Number of women not stated 498 neonates	Betamethasone No ACS	No ACS, 10–23 h, 24 h–47 h, 2–7 d, >7 d from administration of 1st dose	Neonatal mortality, RDS, IVH, NEC
Gaur 2017^38^	Prospective cohort	India	Lower middle income	Women aged 18–45 with singleton or multiple pregnancy and without diabetes or other illness	<37	123 women 111 neonates	Betamethasone	<24 h, >24 h from single dose	Perinatal and neonatal mortality, RDS
Gulersen 2021^39^	Retrospective cohort	USA	High income	Women at risk of late preterm birth. Singleton neonates from 34^0^–36^6^	34^0^–36^6^	1248 women 1248 Neonates	Betamethasone	<2 d, 2–7 d, >7 d from administration of 1st dose	RDS, Neonatal hypoglycaemia
Guruvare 2015^40^	Retrospective cohort	India	Lower middle income	Singleton preterm neonates from 28 to 34 weeks GA	28–34	Number of women not stated 284 neonates	Betamethasone and Dexamethasone	0–7 d, 8–14 d, 15–21 d, 22–28 d, >29 d (dose/course not specified)	RDS
Haas 2006^41^	Retrospective cohort	USA	High income	Singleton neonates without congenital anomalies or fetal demise	24–36	166 women 163 neonates	Betamethasone	<24 h, 24–<48 h from 1st dose	Neonatal mortality; RDS; IVH; BPD; NEC
Hurrell 2022^42^	Prospective cohort and RCT (secondary analysis)	UK and Ireland	High income	Women with confirmed preeclampsia delivering before 35 weeks' gestation	<35	250 women 250 neonates	Betamethasone or dexamethasone No ACS	No ACS, ≤7 days, >7 days (dose/course not specified)	Perinatal mortality, neonatal mortality, RDS, BW
Janssen 2021^43^	Retrospective cohort	USA	High income	Neonates born between 34 and 37 weeks' gestational age	34–<36 36–<37	423 women 500 neonates	Betamethasone No ACS	No ACS, within 2 days, within 7 days from administration of last dose.	RDS
Karmoker 2020^44^	Retrospective cohort	Bangladesh	Lower middle income	Singleton neonates without congenital anomalies	24–36	200 women 200 neonates	Dexamethasone	>48 h to <7 days, 7–14 days from administration (not otherwise specified)	Neonatal mortality; RDS; IVH; NEC; CAM
Kosinska-Kaczynska 2016^45^	Retrospective cohort	Poland	High income	Women with twin pregnancy	26^0^–33^6^	106 women 211 neonates	Betamethasone or dexamethasone	<7 days, ≥7 days from completion of ACS course	Perinatal mortality; RDS; BPD; IVH; NEC; sepsis; mean BW; NICU admission
Kuk 2013^46^	Retrospective cohort	South Korea	High income	Twin neonates	23–34	234 women 468 neonates	Betamethasone or dexamethasone No ACS	<2 days, 2–7 days, >7 days from 1st dose	Neonatal mortality; RDS; IVH; sepsis; NEC; mean BW; NICU admission; CAM
Kyser 2012^47^	Retrospective cohort	USA	High income	Singleton or multiple neonates with BW 401–1000 g and without major anomalies admitted to the NICU	22–25	Number of women not reported 237 neonates	Unspecified ACS	<7 days, 2 doses between 12 h and 7 days of birth, 1 dose <12 h from ACS	Neonatal mortality
Lau 2017^48^	Retrospective cohort	Singapore	High income	Singleton and multiple neonates	23^5^–36^6^	302 women 352 neonates	Dexamethasone	<48 h, 48 h to 7 days, >7 days from 1st dose	RDS; mean BW
Li 2022^49^	Retrospective cohort	China	Upper middle income	Neonates born <32 weeks' gestation, who were transferred to the NICU within 2 h of birth	24–32	706 women 706 neonates	Dexamethasone	<24 h, 1–2 days, 2–7 days, >7 days from administration of 1st dose	Neonatal mortality, RDS, BPD,
Liebowitz 2016^50^	Prospective cohort	USA	High income	Singleton and multiple neonates without major anomalies admitted to the NICU.	<28	Number of women not reported 667 neonates	Betamethasone	within 6 h, 7–23 h, ≥24 h, <10 days, ≥10 days from 1st dose	Neonatal mortality; BPD; IVH; sepsis; NEC; mean BW; CAM
McEvoy 2008^51^	Prospective cohort	USA	High income	Singleton and multiple neonates with BW ≤2000g and without congenital anomalies	25–32	Number of women not reported 56 neonates	Betamethasone	<7 days, ≥7 days from completion of course	Perinatal mortality; neonatal mortality; RDS; mean BW
Melamed 2015^52^	Retrospective cohort	Canada	High income	Singleton live-born neonates admitted to level III NICU	24^0^–33^6^	6870 women 6870 neonates	Betamethasone or dexamethasone No ACS	<24 h, >24 h and <7 days, >7 days from 1^st^dose	Neonatal mortality; BPD; IVH; mean BW; NEC
Nagy 1978^53^	Prospective cohort	Hungary	High income	Women with singleton or multiple pregnancy at risk of preterm birth	≤37	577 women 460 neonates	Dexamethasone No ACS	≤48 h, >48 h from 1^st^dose	Perinatal mortality; RDS
Nair 2009^54^	Retrospective cohort	USA	High income	Singleton neonates without congenital anomalies admitted to NICU	24–28	163 women 163 neonates	Unspecified ACS No ACS	<24 h from 1st dose	Neonatal mortality; RDS; BPD; IVH; CAM
Norberg 2017^55^	Prospective cohort	Sweden	High income	Singleton and multiple neonates, including with congenital anomalies	22–26	Number of women not specified 707 neonates	Unspecified ACS No ACS	<24 h, 24–47 h, 48 h to 7 days, >7 days from 1st dose	Neonatal mortality
Norman 2017^56^	Prospective cohort	Belgium, Denmark, Estonia, France, Germany, Italy, Netherlands, Poland, Portugal, Sweden, UK	High income	Singleton live births	24–31	4594 women 4594 neonates	Betamethasone or dexamethasone No ACS	<24 h, 24 h to 7 days, >7 days from 1st dose	Neonatal mortality
Palas 2018^57^	Prospective cohort	France	High income	Twin neonates admitted to NICU	24–31	390 women 750 neonates	Betamethasone No ACS	≤7 days, >7 days from 1st dose	Neonatal mortality; BPD
Peaceman 2005^58^	Retrospective cohort	USA (Chicago)	High income	Singleton and multiple neonates	26–34	162 women 197 neonates	Betamethasone or dexamethasone	≤7 days, >7 days from 1st dose	Neonatal mortality; IVH; sepsis; NEC; mean BW
Ring 2007^59^	Retrospective cohort	USA	High income	Singleton neonates without congenital anomalies	26–34	357 women 357 neonates	Betamethasone or dexamethasone	>48 h to 14 days, >14 days from 1st dose	CAM
Ryu 2019^60^	Retrospective cohort	South Korea	High income	Singleton neonates born to women with and without histological CAM	23^0^–33^6^	254 women 254 neonates	Betamethasone or dexamethasone No ACS	2–7 days, <48 h or >7 days from 1^st^dose	Neonatal mortality; RDS; BPD; IVH; sepsis; NEC
Schmidt 2011^61^	Retrospective cohort	Canada, US, Australia, NZ, Hong Kong	High income	Singleton and multiple neonates with BW 500–999 g	NR	Number of women not specified 1195 neonates	Unspecified ACS No ACS	<24 h, 24 h to 7 days, >7 days before birth	Neonatal mortality; IVH
Sehdev 2004^62^	Retrospective cohort	USA	High income	Singleton neonates with birth weight 500–1500 g born to women admitted for preterm labour, PROM, or indicated for labour (CAM, non-reassuring fetal testing)	<28	325 women 325 neonates	Betamethasone	<24 h, 24–48 h, 48 h to 7 days, >7 days from 1st dose	Neonatal mortality, RDS, BPD, IVH, NEC, mean BW, CAM
Sekhavat 2011^63^	Prospective cohort	Iran	Lower middle income	Singleton neonates	28–34	104 women 104 neonates	Dexamethasone	<2 days, 2–7 days, >7 days from 1st dose	RDS, mean BW
Sen 2002^64^	Retrospective cohort	UK	High income	Singleton and multiple neonates admitted to NICU having received surfactant within first 2hrs of life	<31	Number of women not reported 226 neonates	Betamethasone No ACS	4–24 h, 24 h to 7 days from 1st dose	Neonatal mortality, IVH, NEC, mean BW
Siegler 2022^65^	Retrospective cohort	Israel	High income	Singleton neonates	24–<34	327 women 327 neoates	Betamethasone	<2 days, 2–7 days from administration of 1st dose	Neonatal mortality, RDS, BPD, IVH, BW, NEC
Tomotaki 2021^66^	Retrospective cohort	Japan	High income	VLBW neonates	<30	Number of women not reported 115 neonates	Betamethasone	No ACS or less than 24 h, 24 h to 7 days, >8 days from administration of last dose	RDS, BPD, IVH, ROP, PDA, CAM
Vermillion 2001^67^	Retrospective cohort	South Carolina, USA	High income	Women with singleton pregnancy, intact membranes and no fetal anomalies	28–34	216 women 216 neonates	Betamethasone	1–2 days, 3–7 days, 8–14 days from 1st dose	RDS, IVH, sepsis, mean BW, CAM
Waters 2009^68^	Retrospective cohort	USA	High income	Singleton neonates without congenital anomalies	30–33^6^	524 women 524 neonates	Betamethasone or dexamethasone No ACS	<48 h, 48 h to 7 days, >7 days from ACS (does not specify dose or course)	Neonatal mortality, RDS
Wilms 2011^69^	Retrospective cohort	Netherlands	High income	Singleton or multiple neonates	24^5^–34	220 women 254 neonates	Betamethasone	0–7 days, 8–14 days, 15–21 days, 22–28 days from 1st dose	RDS, BPD
Wong 2014^70^	Retrospective cohort	Australia	High income	Singleton or multiple neonates without congenital anomalies admitted to NICU	<29	Number of women not reported 2549 neonates	Betamethasone or dexamethasone No ACS	<24 h, 48 h to 7 days, >7 days from 1st dose	Neonatal mortality, BPD, IVH, sepsis, CAM
Yasuhi 2017^71^	Retrospective cohort	Nagasaki, Japan	High income	Women with singleton pregnancy with no fetal anomalies	24–33	397 women 397 neonates	Betamethasone	<7 days, 7–14 days, >14 days from 2nd dose	RDS, mean BW, CAM
Caspi 1976^72^	Case-control	Israel	High income	Women with singleton or multiple pregnancy with threatened preterm birth	28–36	55 women 60 neonates	Dexamethasone No ACS	Days: 1, 2, 3,4, 5, 6, 7 from 1st dose	RDS
Madarek 2003^73^	Case-control	Iran	Lower middle income	Women with singleton pregnancies giving birth preterm	26–36	300 women 300 neonates	Dexamethasone	<24 h, 24–48 h, >48 h from 1st dose	Neonatal mortality

Notes: ACS = antenatal corticosteroid; BPD = bronchopulmonary disease; BW = birth weight; CAM = chorioamnionitis; GA = gestational age at trial entry; IUGR = intrauterine growth restriction; IVH = intraventricular haemorrhage; NEC = necrotising enterocolitis; NICU = neonatal intensive care unit; PROM = premature rupture of membranes; RDS = respiratory distress syndrome.

#### Randomised trials

Eight trials were conducted in high-income countries (HIC), with one trial conducted in an upper middle income country (South Africa), and one trial conducted in five low- and middle-income countries (LMIC) (Table 1). The 10 trials included 4592 women and 5018 neonates, the majority of which are from the WHO ACTION-I trial.^13^^,^^74^ The WHO ACTION-I secondary analysis on women who received a single course of ACS was included here,^74^ however the original trial was excluded due to the inclusion of women who received multiple courses of ACS (∼5% of the included population).^13^ All but one recruited women with singleton or multiple pregnancies. Gestational age at trial entry ranged from 24 to 37 weeks, though one trial did not report the gestational age range.^18^ Six trials included babies born extremely preterm (<28 weeks’ gestation).^17^^,^^21^, ^22^, ^23^, ^24^^,^^74^ Three trials did not exclude late preterm births (>34 weeks).^17^^,^^22^^,^^25^ Three trials excluded women with hypertensive disorders of pregnancy,^23^, ^24^, ^25^ and one excluded FGR pregnancies.^24^ Five trials included women with hypertension/hypertensive disorders of pregnancy.^17^^,^^20^, ^21^, ^22^^,^^26^ The inclusion of women with hypertensive disorders of pregnancy or SGA pregnancies was not reported in two^18^^,^^19^ and nine trials,^17^, ^18^, ^19^, ^20^, ^21^, ^22^, ^23^^,^^25^^,^^26^ respectively. No trials specifically excluded women based on mode of delivery, but three trials did not report any information on mode of delivery.^18^^,^^22^^,^^24^ ACS administration-to-birth interval was defined by the time from first ACS dose in all but one study. Each of the 10 trials used a unique combination of time intervals for reporting findings, however the time intervals <24 h, 1–7 days and >7 days were used by more than one study (Table 1, Table S1). Six trials had a high risk of bias, two trials had some concerns for risk of bias, and two had a low risk of bias (Figs. S1 and S2). Data were available for the outcomes RDS (9 trials), neonatal mortality (3 trials) and perinatal mortality (2 trials), CAM (1 trial), NEC (1 trial) and IVH (1 trial). No data were reported for other review outcomes.

#### Observational studies

Of the 45 cohort studies, 32 were conducted in HICs (Table 2). These studies included at least 22,992 women (some studies did not report the total number of women) and 30,974 neonates. The studies were heterogeneous in terms of plurality, gestational age ranges of participants and time intervals reported. Participants were women with either singleton or multiple pregnancy (14 studies), singleton pregnancies only (23 studies) or twin pregnancies only (four studies). Four studies did not report singleton vs multiple pregnancies. Gestational ages ranged from 20 to 37 weeks; however few studies used the same gestational age range and one studies did not specify.^61^ Seven studies included only women who delivered extremely preterm (<29 weeks).^32^^,^^47^^,^^50^^,^^54^^,^^55^^,^^62^^,^^70^ In contrast, 10 studies did not exclude late preterm births (>34 weeks).^28^^,^^38^^,^^39^^,^^41^, ^42^, ^43^^,^^48^^,^^53^^,^^72^^,^^73^ Three studies excluded women with hypertensive disorders of pregnancy,^28^^,^^63^^,^^72^ and two excluded SGA pregnancies.^28^^,^^37^ Most trials reported that women with hypertensive disorders of pregnancy were included (41 studies) and 15 studies reported the inclusion of SGA pregnancies. Two study included only women with severe pregnancy-induced hypertension or confirmed pre-eclampsia.^35^^,^^42^ The inclusion of women with hypertensive disorders of pregnancy or SGA pregnancies was not reported in 11 and 34 studies, respectively. No studies specifically excluded women based on mode of delivery, but 10 studies did not report any information on mode of delivery.^29^^,^^37^^,^^38^^,^^44^^,^^59^^,^^64^^,^^68^^,^^69^^,^^72^^,^^73^ The definition of the start time of ACS administration was from first dose in 31 studies, while the remainder used different starting points (15 studies) or did not specify (seven studies). One study had a low risk of bias, four studies had a critical risk of bias (attributed to bias due to confounding factors), 18 had a serious risk of bias, and the remainder were moderate risk of bias (Figs. S3 and S4). The most commonly reported outcomes were neonatal mortality (28 studies), RDS (30 studies), IVH (20 studies), mean birthweight (21 studies), NEC (15 studies), BPD (17 studies), chorioamnionitis (13 studies), neonatal sepsis (13 studies), patent ductus arteriosus (8 studies), retinopathy of prematurity (7 studies), perinatal mortality (4 studies), NICU admission (2 studies) and neonatal hypoglycaemia (2 studies). No data were available for maternal mortality, endometritis, and maternal intensive care unit admission.

Two case–control studies were included (355 women, 360 neonates), one from a HIC (Israel), and one from a LMIC (Iran) (Table 2). Gestational age ranged from 26 to 36 weeks. The two studies used different ACS administration-to-birth intervals, and both had an overall critical risk of bias (Figs. S3 and S4). Outcome data were available for RDS (one study) and neonatal mortality (one study).

### Perinatal mortality

Perinatal mortality was reported for different ACS intervals in two trials and four cohort studies (Tables S2 and S3). One trial (2904 neonates) reported decreased odds of perinatal mortality at >7 days compared to no ACS,^26^ whereas the other trial reported no differences.^19^ One observational study (460 newborns) had decreased odds of perinatal mortality at >48 h compared to no ACS (OR 0.41, 95% CI 0.18–0.93), but not at <48 h,^53^ whereas the other three studies did not detect differences.

### Neonatal mortality

**Fig. 2:**
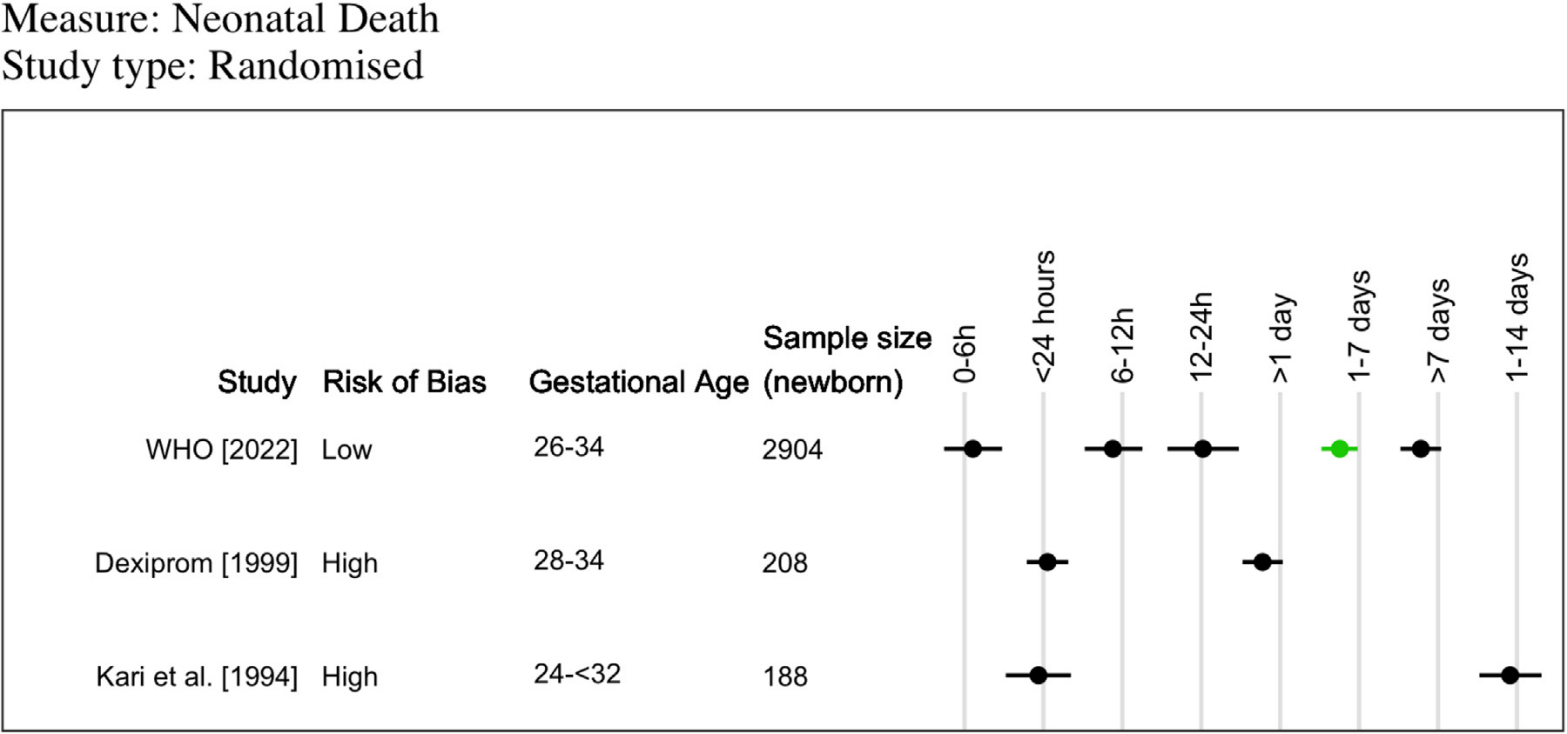
**Descriptive summary of reported neonatal mortality outcomes from randomised controlled trials.** A visual representation summarising odds ratio (or relative risk—WHO 2020 trial) of neonatal mortality at various time intervals compared to “no antenatal corticosteroid” group (3 trials). Green data points indicate a significant reduction in the odds of neonatal mortality. Black data points indicate no effect of ACS on neonatal mortality.

Neonatal mortality was reported in three trials (Table S4). Two trials were small (sample sizes of 208 and 188 newborns) and reported no differences in neonatal mortality across different intervals,^19^^,^^21^ though few intervals were used (Fig. 2). The third trial (2904 newborns) conducted in five LMICs reported that the risk of neonatal death was reduced compared to placebo for intervals of 1–7 days (RR 0.77, 95% CI 0.60–0.98) but not for other time intervals (0–6 h, >6–12 h, >12–24 h, >7 days).^26^

Neonatal mortality was reported in 29 observational studies (Table 3, Table S5). For almost all time intervals investigated it was possible to find a study that reported a beneficial effect associated with that time interval. In nine studies at least one optimal time interval with reduced odds (or risk) of neonatal mortality was identified, though this varied between studies (one study: ≥24 h; three studies: 1–7 days; one study: 1–2 days, 2–7 days and >7 days; three studies: 2–7 days; one study: 2–10 days; one study: 1–7 days and >7 days; one study: ≤7 days; one study:<14 days). In the other 20 studies, no optimal time interval was identified.

**Table 3:** Summary of findings on association between antenatal corticosteroid administration-to-birth intervals and maternal and newborn outcomes.

Outcome	Findings from trials on antenatal corticosteroid administration-to-birth interval	Findings from observational studies on optimal^a^ antenatal corticosteroid administration-to-birth interval	Findings from observational studies that used “no antenatal corticosteroid” as a comparator
Perinatal mortality	• No difference reported (1 trial) • Perinatal mortality reduced at >7 days compared to placebo, but not for <6 h, >6–12 h, >12–24 h, 1–7 days (1 trial)	• No optimal time interval identified (3 studies) • >48 h (1 study)	>**48 h vs no ACS** • 1 study—reduced odds of perinatal death **>7 days vs no ACS** • 1 study—no difference
Neonatal mortality	• No differences reported (2 trials) • Neonatal death reduced at 1–7 days compared to placebo, but not for <6 h, >6–12 h, >12–24 h and >7 days (1 trial)	• No optimal interval identified (20 studies) • 2–7 days optimal (3 studies) • ≥24 h optimal (1 study) • 1–7 days optimal (2 studies) • >7 days optimal (1 study) • ≤7 days optimal (1 study)	**<24 h vs no ACS** • 5 studies—no difference • 2 studies–reduced odds of neonatal death • 1 study—increased odds of neonatal death **4**–**24 h vs no ACS** • 1 study—reduced odds of neonatal death **>1 day vs no ACS** • 1 study—no difference **<2 days vs no ACS** • 2 studies—no difference **1**-**2 days vs no ACS** • 1 study—no difference • 1 study—reduced odds of neonatal death **1**–**7 days vs no ACS** • 3 studies—reduced odds of neonatal death **2**–**7 days vs no ACS** • 2 studies—no difference • 2 studies—reduced odds of neonatal death **<7 days vs no ACS** • 1 study—reduced odds of neonatal death ≤7 **days vs no ACS** • 1 study—no difference **>7 days vs no ACS** • 3 studies—reduced odds of neonatal death • 1 study—no difference **<2 or >7 days vs no ACS** • 1 study—no difference
Respiratory distress syndrome	• No differences reported (8 trials) • RDS reduced at 1–7 days and >7 days compared to placebo (1 trial)	• No optimal time interval (13 studies) • 24–<48 h optimal (2 studies) • 1-2 days optimal (1 study) • 1–7 days optimal (3 studies) • Within 2 days optimal (1 study) • >2 days optimal (1 study) • 2–7 days optimal (6 studies) • 2–<7 days optimal (1 study) • Within 7 days optimal (1 study) • <7 days optimal (1 study) • >7 days optimal (2 studies) • 7–14 days optimal (1 study) • 22–28 days optimal (1 study) • >29 days optimal (1 study)	**<24 h vs no ACS** • 3 studies—no difference **<2 days vs no ACS** • 2 studies—no difference **1**-**2 days vs no ACS** • 1 study—reduced odds of RDS **>2 days vs no ACS** • 1 study—reduced odds of RDS **2**–**7 days vs no ACS** • 2 studies—no difference • 1 study—reduced odds of RDS ≤7 **days vs no ACS** • 1 study—no difference **>7 days vs no ACS** • 1 study—reduced odds of RDS • 1 study—no difference • 1 study—increased odds of RDS **<2 or >7 days vs no ACS** • 1 study—no difference
Intraventricular haemorrhage	• Too few events (1 trial)	• No optimal time interval identified (11 studies) • 7–23 h (1 study) • <24 h (2 studies) • ≥24 h (1 study) • 1–<7 days (1 study) • 1–7 days (4 studies) • <2–7 days (1 study) • 2–7 days (1 study) • 2–<7 days (1 study) • ≥7 days (1 study) • >7 days (5 studies) • 7–<14 days (1 study) • ≥10 days (1 study) • ≥14 days (1 study)	**<24 h vs no ACS** • 3 studies—no difference • 3 studies—reduced odds of IVH **1**–**7 days vs no ACS** • 4 studies—reduced odds of IVH **2**–**7 days vs no ACS** • 1 study—no difference • 1 study—reduced odds of IVH **>7 days vs no ACS** • 5 studies—reduced odds of IVH
Necrotising enterocolitis	• No differences reported (1 trial)	• No optimal time interval identified (14 studies) • <2 days (1 study)	**<24 h vs no ACS** • 2 studies—no difference **4**–**24 h vs no ACS** • 1 study—no difference **<2 days vs no ACS** • 1 study—no difference **1**-**2 days vs no ACS** • 1 study—no difference **1**–**7 days vs no ACS** • 1 study—no difference **2**–**7 days vs no ACS** • 1 study—no difference **>7 days vs no ACS** • 1 study—no difference • 1 study—increased odds of NEC
Broncopulmonary dysplasia	None	• No optimal time interval identified (14 studies) • <2 days (1 study) • 1–7 days (1 study) • ≤7 days (1 study) • >7 days (1 study)	**<24 h vs no ACS** • 4 studies—no difference **1**–**7 days vs no ACS** • 1 study—no difference **2**–**7 days vs no ACS** • 2 studies—no difference • 1 study—increased odds of BPD **<7 days vs no ACS** • 1 study—decreased odds of BPD **>7 days vs no ACS** • 2 studies—no difference • 1 study—decreased odds of BPD **<2 or >7 days vs no ACS** • 1 study—no difference
Neonatal Sepsis	None	• No optimal time interval identified (13 studies)	**<24 h vs no ACS** • 4 studies—no difference **1**–**7 days vs no ACS** • 1 study—no difference **2**–**7 days vs no ACS** • 4 studies—no difference • 1 study—increased odds of sepsis **>7 days vs no ACS** • 4 studies—no difference • 1 study—increased odds of sepsis **<2 or >7 days vs no ACS** • 1 study—no difference
NICU admission	None	• No optimal time interval identified (2 studies)	**N/A**
Neonatal hypoglycaemia	None	• No optimal time interval identified (1 study) • >7 days (1 study)	**N/A**
Retinopathy of prematurity	None	• No optimal time interval identified (7 study)	**N/A**
Patent ductus arteriosus	None	• No optimal time interval identified (6 study) • >7 days (2 studies)	**N/A**
Birthweight	None	• No optimal time interval identified (11 studies) • Optimal time interval not reported (4 studies) • <2 days (1 study) • >7 days (3 studies) • >10 days (1 study) • >14 days (1 study)	**N/A**
Chorioamnionitis	• No difference (1 trial)	• No optimal time interval identified (11 studies) • <2 days (1 study) • No ACS or <6 h (1 study)	**<24 h vs no ACS** • 3 studies—no difference **<2 days vs no ACS** • 1 study—no difference **1**–**7 days vs no ACS** • 1 study—increased odds of chorioamnionitis **2**–**7 days vs no ACS** • 2 studies—no difference **>7 days vs no ACS** • 1 study—no difference • 2 studies—increased odds of chorioamnionitis

ACS = antenatal corticosteroid.

a “Optimal” was defined as those intervals associated with statistically significant reduced odds (or risk) of outcome under consideration.

**Fig. 3:**
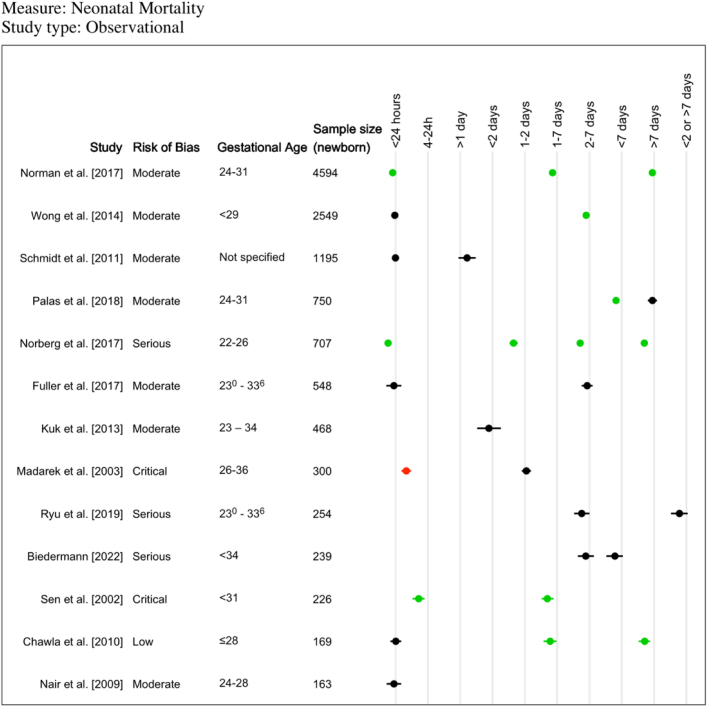
**Descriptive summary of reported neonatal mortality outcomes from observational studies.** A visual representation summarising odds ratio of neonatal mortality for different time intervals compared to “no antenatal corticosteroid” group (13 studies; 16 additional studies did not include a “no antenatal corticosteroid” group or had two few events). Green data points indicate a statistically significant decrease in odds ratio for neonatal mortality (i.e. upper bound of 95% CI was below 1). Red data points indicate a statistically significant increase in the odds of neonatal mortality (i.e. lower bound was above 1). Black data points indicate the odds ratio of neonatal mortality was not significantly different (i.e. 95% CI included 1).

Thirteen studies reported OR for neonatal mortality using the “no ACS” group as a comparator, only three of which had a sample size greater than 1000 newborns (Fig. 3). For <24 h, two studies (4594 and 707 newborns) found reduced odds of neonatal mortality compared to no ACS (OR 0.45, 95% CI 0.35–0.6 and OR 0.27, 95% CI 0.15–0.47, respectively)^55^^,^^56^ while one case–control study of 300 newborns^73^ (critical risk of bias), reported increased odds of neonatal mortality (OR 6.37, 95% CI 2.75–14.47). The other five studies found no differences for <24 h.^32^^,^^37^^,^^54^^,^^61^^,^^70^ For 1–2 days, one study (707 newborns) found reduced odds of neonatal death compared to no ACS (OR 0.11, 95% CI 0.05–0.25),^55^ while one study reported no differences.^73^ For <2 days, two studies found no differences.^31^^,^^46^ For 1–7 or 2–7 days, five studies (169, 226, 707, 2549 and 4594 newborns) found reduced odds of neonatal mortality compared to no ACS,^32^^,^^55^^,^^56^^,^^64^^,^^70^ and three studies (239, 254 and 548 newborns) showed no differences.^31^^,^^37^^,^^60^ For >7 days, three studies (169, 707 and 4594 newborns) found reduced odds of neonatal mortality,^32^^,^^55^^,^^56^ though one (750 newborns) found no difference.^57^ Only one study was identified for the remaining five time-intervals.

A secondary analysis of the WHO ACTION-I trial (2638 women from lower middle income countries) reported relative risk of neonatal mortality with administration to birth interval as a continuous variable from 0 to 28 days, for different gestational ages (from 26 to 33 weeks).^74^ At all gestational ages, risk of neonatal mortality consistently reduced with increasing time from first dose of ACS, reaching a nadir at 13–14 days. The reduction in risk was diminished after this time interval, and risk of neonatal mortality began to increase as the interval of 28 days approached. No benefit or harm was observed from 0 to 24 h. In contrast, one cohort of 4594 women from high income countries reported that relative risk of neonatal mortality rapidly decreased at <12 h, followed by a slower decreased reaching a plateau of >50% risk reduction after 18–36 h.^56^ At 5–7 days or more, the confidence intervals suggest an increase in risk of neonatal mortality from the plateau.

### Respiratory distress syndrome

**Fig. 4:**
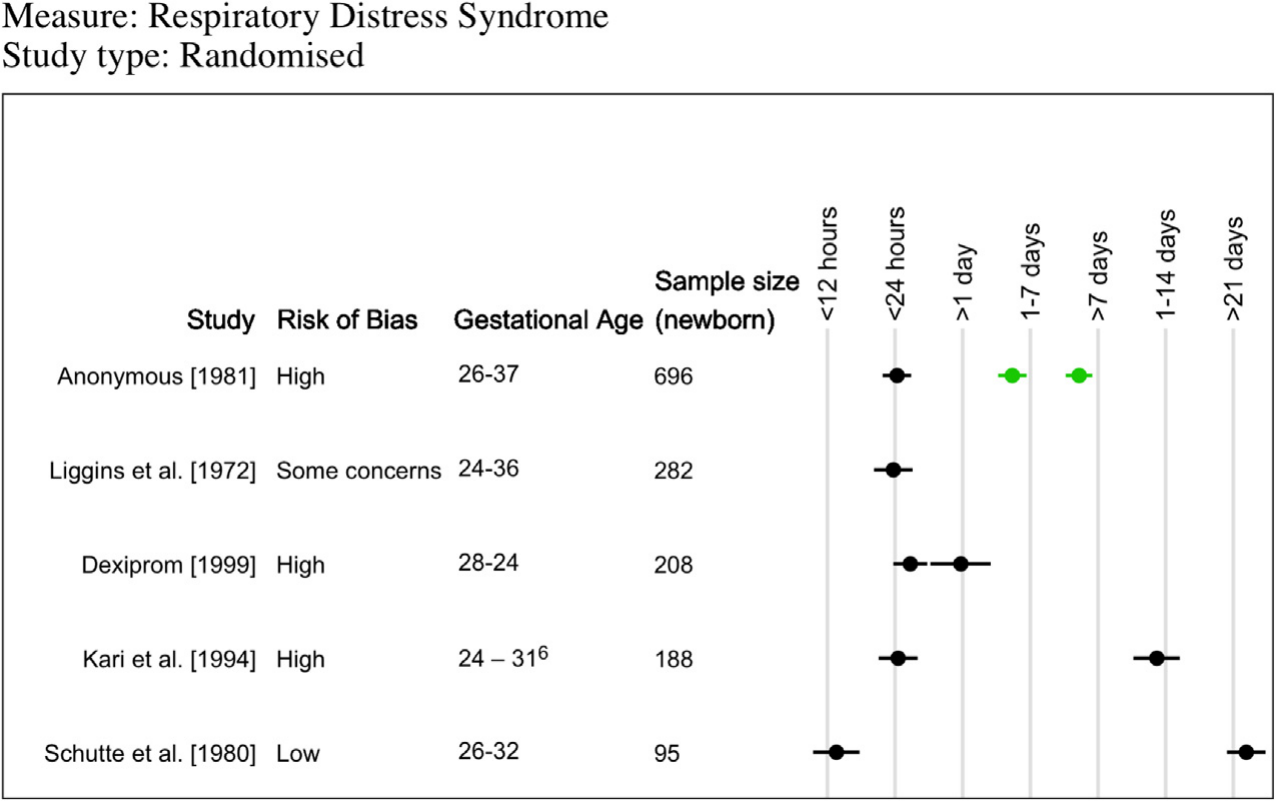
**Descriptive summary of reported Respiratory Distress Syndrome outcomes from randomised controlled trials.** A visual representation summarising odds ratio of respiratory distress syndrome for different time intervals compared to “no antenatal corticosteroid” group (5 trials; 4 additional trials did not include a “no antenatal corticosteroid” group or had two few events). Green data points indicate a statistically significant decrease in odds ratio for respiratory distress syndrome (i.e. upper bound of 95% CI was below 1). Black data points indicate the odds ratio of respiratory distress syndrome was not significantly different (i.e. 95% CI included 1).

RDS was reported in nine trials (Table S6), 8 of which included a “no ACS” groupand reported OR for nine different intervals (Fig. 4). Four small trials (ranging from 95 to 282 newborns) reported that odds of RDS were not different for any ACS administration-to-birth interval.^19^^,^^21^^,^^22^^,^^24^ One trial of 696 newborns found a reduction in odds of RDS at 1–7 days (OR 0.46, 95% CI 0.25–0.86) and >7 days (OR 0.29, 95% CI 0.12–0.69) compared to no ACS.^17^

RDS was reported for different intervals in 29 cohort studies and one case–control study (Table 3, Table S7). In 17 studies, at least one optimal time interval was identified, though it varied between studies (two studies: 24–<48 h; one study: 1 and 2 days; one study: ≤2 days; three studies: 1–7 days; one study: >2 days; six studies: 2–7 days; one study: 2–<7 days; one study: <7 days; one study: ≤7 days two study: >7 days; one study: 7–14 days; one study: 22–28 days; one study: >29 days). In the remaining 13 studies no optimal time interval was identified.

**Fig. 5:**
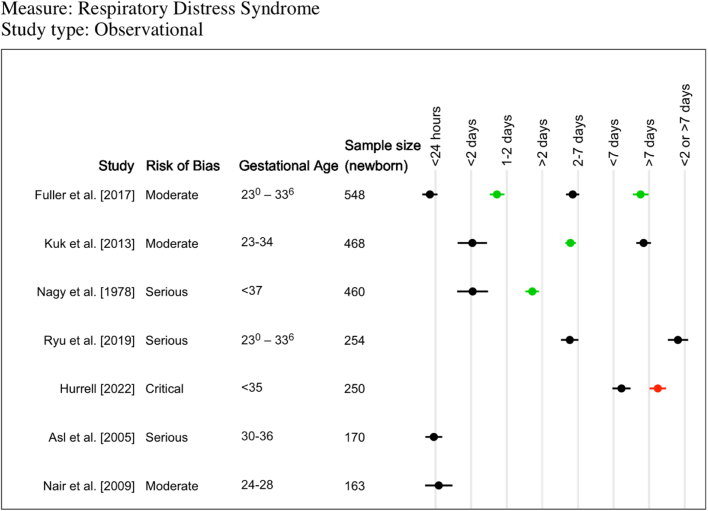
**Descriptive summary of reported Respiratory Distress Syndrome outcomes from observational studies.** A visual representation summarising odds ratio of respiratory distress syndrome for different time intervals compared to “no antenatal corticosteroid” group (7 studies; 23 additional studies did not include a “no antenatal corticosteroid” group or had too few events). Green data points indicate a statistically significant decrease in odds ratio for respiratory distress syndrome (i.e. upper bound of 95% CI was below 1). Black data points indicate the odds ratio of respiratory distress syndrome was not significantly different (i.e. 95% CI included 1).

Amongst these 30 studies, seven studies reported OR using the “no ACS” group as a comparator for eight different intervals, though few intervals were common across studies (Fig. 5). For <24 h, three studies found odds of RDS were not different compared to no ACS.^28^^,^^37^^,^^54^ For 2–7 days, two studies found no differences,^37^^,^^60^ though one study (468 twins) found reduced odds of RDS (OR 0.48, 95% CI 0.29–0.82).^46^ Only one or two studies were identified for the remaining six time intervals. A significant reduction in odds of RDS was found at 1 and 2 days,^37^ >2 days,^53^ and >7 days.^37^

One trial reported the frequency of RDS against the administration to birth interval as a continuous variable in 208 women and demonstrated that betamethasone provided a benefit over no ACS up to 2 weeks.^20^

### Intraventricular haemorrhage

IVH was reported in one trial of 188 newborns (Table S8).^21^ This trial was assessed as high risk of bias and reported a lower prevalence of IVH at 1–14 days (three events in 41 newborns) compared to no ACS (18/64), though no difference was observed at <24 h (4/20). IVH was reported in 20 cohort studies (Table 3, Table S9)–optimal time intervals were identified in nine studies (two studies: <24 h, six studies: 1–7 days, one study: <2–7 days, one study: 2–<7 days, five studies: >7 days, one study: 7–<14 days, one study: ≥14 days). In 11 studies no optimal time interval was identified.

Seven observational studies used a “no ACS” group as a comparator for four different intervals (Fig. S5). At <24 h compared to no ACS, two studies (1195 and 169 newborns) found reduced odds of IVH,^32^^,^^61^ while four studies found no differences.^36^^,^^37^^,^^54^^,^^70^ For 1–7 days, four studies found reduced odds of IVH.^32^^,^^36^^,^^61^^,^^64^ At 2–7 days, one study (548 newborns) found no differences,^37^ though one study (2549 newborns) found reduced odds of IVH.^70^ At >7 days, all five studies found that odds of IVH were reduced.^32^^,^^36^^,^^37^^,^^61^^,^^70^

One cohort of 4594 women from high income countries reported relative risk of IVH with administration-to-birth interval as a continuous variable.^56^ Relative risk of IVH was associated with longer administration-to-birth intervals, until 5–7 days which were associated with increasing risk.

### Necrotising enterocolitis (NEC)

NEC was reported in one trial of 208 newborns (Table S10). This trial found no difference in the odds of NEC at <24 h or >24 h compared to no ACS.^19^ NEC was reported for different intervals in 15 cohort studies (Table 3, Table S11). One study (2259 newborns) reported that, compared to <2 days, odds of developing NEC were increased at 2–7 days and 7–14 days, but were not different at ≥14 days compared to <2 days.^30^ The remaining 14 studies did not identify an optimal interval for this outcome. Four studies reported OR using the “no ACS” group as a comparator for seven different intervals (Fig. S6). Three studies found no differences at any interval,^37^^,^^46^^,^^64^ though one study (169 newborns) reported increased odds of NEC at >7 days (OR 4.35, 95% CI 1.1–17.23).^32^

### Chronic lung disease (bronchopulmonary dysplasia)

BPD was reported for different intervals in 17 cohort studies, though 14 studies did not find an optimal interval (Table 3, Table S12). One study (2259 newborns) identified increased odds of BPD at 2–7 days and 7–14 days compared to <2 days, but no differences for intervals >14 days and <2 days.^30^ A second study (6870 newborns admitted to NICU) identified increased odds of BPD at <24 h and >7 days compared to 1–7 days.^52^ Six studies reported OR using the no ACS group as a comparator for six different intervals (Fig. S7). All studies reporting on <24 h found no differences in odds of BPD.^32^^,^^36^^,^^54^^,^^57^^,^^60^^,^^70^ For 2–7 days, one study (2549 newborns) found increased odds of BPD,^70^ though two other studies (672 and 254 newborns) found no differences.^36^^,^^60^ Findings were conflicting for >7 days—one study (750 newborns) found reduced odds of BPD,^57^ whereas two studies (672 and 169 newborns) found no difference.^32^^,^^36^ The remaining three intervals had a single study each; one study found decreased odds of BPD at <7 days.^57^ Mortality can be a competing outcome with BPD, however few studies reported a composite outcome of BPD and mortality.^32^^,^^50^^,^^60^

### Neonatal sepsis

Neonatal sepsis was reported for different intervals in 13 cohort studies (Table 3, Table S13), though none identified an optimal interval. Six of these studies reported OR using the “no ACS” group as a comparator for five different intervals (Fig. S8). One study (2549 newborns) found increased odds of neonatal sepsis at 2–7 days (OR 1.39, 95% CI 1.07–1.81) and >7 days (OR 1.32, 95% CI 1.12–1.56).^70^ No association with sepsis was found for other time points.

### NICU admission

NICU admission was reported in two cohort studies (Table 3, Table S14), both of which found no differences between time intervals.

### Neonatal hypoglycaemia

Neonatal hypoglycaemia was reported in two cohort studies (Table 3, Table S15). One study (1248 newborns) reported increased odds of hypoglycaemia at <2 days and reduced odds of hypoglycaemia at >7 days, compared to 2–7 days.^39^ The other study (99 newborns) found no difference.^33^

### Retinopathy of prematurity

Retinopathy of prematurity was reported in seven cohort studies (Table 3, Table S16), all of which found no differences between time intervals. Due to significant heterogeneity in the definitions of retinopathy of prematurity reported across studies, data has not been presented as a descriptive forest plot.

### Patent ductus arteriosus

Patent ductus arteriosus was reported in eight cohort studies (Table 3, Table S17). Two studies reported decreased odds patent ductus arteriosus at >7 days, compared to no ACS (OR 0.51, 95% CI 0.27–0.97, 468 twins; and OR 0.70, 95% CI 0.60–0.82, 2549 neonates).^46^^,^^70^ The other studies all found no difference. Due to significant heterogeneity in the definitions of patent ductus arteriosus reported across studies, data has not been presented as a descriptive forest plot.

### Mean birthweight

Birthweight was reported in 21 cohort studies (Table 3, Table S18). Six studies identified an optimal interval (i.e. highest birthweight). Findings varied across studies (one study: <2 days; three studies: >7 days, one study: >10 days, one study: >14 days), but in all but one study the longest interval used was identified as optimal. A further four studies found significant associations between time interval and birthweight but did not report an optimal interval, while the remaining 11 studies found no associations.

### Maternal outcomes

No data were identified for any pre-specified maternal outcomes except chorioamnionitis. This was reported in one trial of 204 women (Table S19) that found no difference in odds at <24 h or >24 h compared to no ACS. Chorioamnionitis was reported in 13 cohort studies (Table 3, Table S20). One study identified an optimal interval–one (2259 women) found reduced odds of chorioamnionitis at <2 days compared to 2 to <7 days, 7 to <14 days and ≥14 days.^29^ Four studies used a “no ACS” group as a comparator for five different intervals (Fig. S9). Two studies showed no differences.^46^^,^^54^ One study (169 newborns) that found increased odds of chorioamnionitis with ACS for 1–7 days (OR 2.97, 95% CI 1.07–8.19) and >7 days (OR 3.31, 95% CI 1.15–9.52).^32^ Another study (2549 newborns; number of women not reported) found increased odds of chorioamnionitis at >7 days (OR 1.74, 95% CI 1.15–2.63).^70^

## Discussion

This systematic review identified 10 randomised trials of 5018 neonates and 47 observational studies of 31,334 neonates from predominantly high-income countries.

Despite the large volume of evidence, studies were heterogeneous in terms of participant characteristics, and outcomes were variably reported using 65 different ACS administration-to-birth intervals. This heterogeneity restricted meaningful evaluation of associations between specific time intervals and outcomes. However, available evidence suggests that the beneficial effects of ACS for some newborn outcomes—such as neonatal mortality, RDS and IVH–possibly varies with different ACS administration-to-birth intervals. While many studies identified an optimal time interval for newborn outcomes, these were not consistent across studies, or for different outcomes. A study identifying a beneficial association for a specific time interval could be found for almost every individual time interval reported. There was insufficient evidence to assess the effects on maternal outcomes, though findings from some observational studies suggest that the risk of chorioamnionitis might be associated with some time intervals.

To the best of our knowledge, this is the first systematic review to explore the role of ACS administration-to-birth interval on maternal and newborn outcomes from randomised and observational studies. The 2006 iteration of the Cochrane review on trials of ACS efficacy included a subgroup analysis that explored the effect of administration to delivery interval.^75^ This Cochrane review used four time intervals: <24 h, <48 h, 1–7 days and >7 days after administration, identifying few trials per interval. The authors reported that neonatal death was reduced at <24 and < 48 h, but not 1–7 or >7 days.^75^ RDS was reduced at <48 h and 1–7 days, but not <24 h or >7 days, and IVH was reduced at <48 h only.^75^ In contrast, trials included in the current review suggest neonatal death and RDS may be reduced at 1–7 days and >7 days. The difference in findings is likely due to the additional trials included in our analysis, particularly the secondary analysis of the WHO ACTION-I trial.^74^ The 2006 Cochrane review authors stated that while these data are from randomised trials, they must be interpreted with caution as the administration-to-birth interval is a post-randomisation variable and may itself be affected by the intervention. For example, if ACS contributed to pregnancy prolongation, the results may be biased. The subgroup analysis was excluded from subsequent review, with the recommendation that individual patient data meta-analysis was preferrable to answer this question.^75^^,^^76^ Observational studies included in our review suggest that neonatal death may be reduced at <24 h, and neonatal death, RDS and IVH might be reduced at 1–7 days and >7 days.

Complicating the analysis of the optimal interval between ACS administration and birth are the many confounding factors that influence neonatal outcomes. For example, longer intervals many present as being more “ideal”, but the additional fetal development that these babies are exposed to must be considered, particularly when comparing short intervals to intervals greater than 7 days. Gestational age at the time of ACS administration is a significant confounding factor in studies examining the efficacy of ACS. Our review included infants across a range of gestational ages. Although the spectrum of morbidity across this range is different, our inclusion criteria aligned with clinical practice for administration of ACS. Unfortunately, the studies included in this systematic review often included a wide range of gestational ages, making sub analysis of gestational age at administration difficult without individual participant data. The seven observational studies that included only babies born <29 weeks’ gestation may shed some light on an optimal time interval in extremely preterm babies, who arguably have the most to benefit from ACS administration. For example, the limited evidence from studies of extremely preterm babies suggests little effect of ACS on odds of neonatal mortality at less than 24 hours. However, any trends observed from this group of studies must be interpreted with caution, as it was rare for these studies to report the same time intervals, allowing for direct comparison.

This systematic review is a comprehensive evaluation of maternal and neonatal outcomes for different ACS administration-to-birth time intervals. We used broad eligibility criteria and a robust search strategy, with screening, extraction and risk of bias assessment conducted in duplicate to minimise errors. Although a large number of studies were identified, variation in time intervals between studies limited meaningful comparisons of available outcome data. Given the considerable heterogeneity in study populations, designs and time intervals, we opted not to pool outcome data, and provided descriptive findings only. An inherent challenge was that administration-to-birth interval was a post-randomisation variable, which increased risk of bias for most studies. Additionally, the lack of studies from LMICs (only 15.8% of included studies), means these findings may be biased towards high-resource settings. We were unable to locate full texts from twenty studies, which may have influenced these findings.

It is well-established that ACS use in women at high risk of early preterm birth can substantively reduce preterm-associated morbidity and mortality.^7^ However, there are differences in global and national-level recommendations regarding the ACS administration-to-birth interval. WHO and Australian guidelines recommend that ACS be given to women when preterm birth is planned or expected within the next seven days, even if birth is likely within 24 h.^77^ The International Federation of Gynecology and Obstetrics (FIGO) similarly recommend ACS even if birth is expected within 18 h,^78^ whereas USA and UK guidelines recommend in favour of ACS for anticipated preterm birth, but do not specify a minimum or optimal interval.^79^^,^^80^ Canadian guidelines state the efficacy of ACS is greatest between 24 h and 7 days, after which there is reduced benefit.^81^ While we are unable to definitively identify an optimal ACS administration-to-birth interval, available evidence suggest that this interval has a key role in maximising benefits for preterm newborns. More advanced statistical modelling, such as those used by three of the studies included in our review,^20^^,^^26^^,^^56^ where the administration-to-birth interval is expressed as a continuous variable can provide an increased understanding of the association between time and the beneficial effects of ACS on neonates. An inherent challenge for this research question is that the administration-to-birth interval can be difficult to predict or modify for individual women. Further research on this topic should consider the use of advanced statistical modelling techniques and individual patient data meta-analysis, as well as explicit consideration of the role of gestational age at time of treatment, which also likely affects newborn outcomes, and any potential harms of steroids, such as a potential association with chorioamnionitis suggested in our data.

This systematic review explored associations between the ACS administration-to-birth interval in pregnant women at risk of preterm birth, identifying 57 studies from predominantly high-income countries. Significant heterogeneity between studies in terms of the enrolled population and time intervals used means that firm conclusions cannot be drawn. However, beneficial effects of ACS for newborn mortality and morbidity appears to vary across different ACS administration-to-birth intervals, suggesting that an optimal ACS interval probably exists. There was insufficient evidence on maternal outcomes, though some time intervals might be associated with chorioamnionitis. Individual patient data meta-analysis or other advanced statistical modelling is likely required to identify the time intervals for which ACS is most beneficial, and how these benefits can be optimised for women and newborns.

## Contributors

JPV and OTO led the conceptualisation and supervision of the project and funding acquisition. AMcD led the development of the protocol and methodology. All authors were involved in conceptualisation of the project and development of the methodology. AMcD, LA and TL descrigned the search strategy and performed literature search. AMcD, LA, TL, JC, GD and JR performed analysis of all data. AMcD and LA performed data visualisation. All authors were involved in interpretation of data. AMcD wrote the original draft of the manuscript and all authors contributed to writing and editing and had full access to the data. AMcD and JPV have accessed and verified all the data in this study. The corresponding author had full access to all the data in the study and had final responsibility for the decision to submit for publication.

## Data sharing statement

All extracted data are available upon appropriate requests by emailing to the authors.

## Declaration of interests

Authors have no competing interests to declare.
