# Supplementary material for: Effect of antenatal corticosteroid administration-to-birth interval on maternal and newborn outcomes: a systematic review
Source: eClinicalMedicine. 2023 Mar 24;58:101916. doi: 10.1016/j.eclinm.2023.101916 (PMC10050784; doi:10.1016/j.eclinm.2023.101916)
Supplement: Table S1 [file mmc1.docx]

**Supplementary Table S1. Antenatal corticosteroid administration-to-birth intervals used in included studies**

| **Interval** | **Trials** | **Cohort studies** | **Case-control studies** |
| --- | --- | --- | --- |
| 0 to 7 days, 8 to 14 days, 15 to 21 days, 22 to 28 days from 1^st^ dose |  | 1 |  |
| 4 to 24 hours, 24 hours to 7 days from 1^st^ dose |  | 1 |  |
| <6 hours, 6 to 12 hours, 12 to 24 hours, 24 hours to 7 days, >7 days from 1^st^ dose | 1 |  |  |
| <6 hours, 7 to 23 hours, ≥24 hours, <10 days, ≥10 days from 1^st^ dose |  | 1 |  |
| <12 hours, 12 hours to 7 days, 8-21 days, >21 days from 1^st^ dose | 1 |  |  |
| <24h or no ACS, 1-7 days, >8 days from last dose |  | 1 |  |
| <24 hours from 1st dose |  | 1 |  |
| <24 hours, >24 hours and <7 days, >7 days from 1^st^dose |  | 1 |  |
| ≤24 hours, >24 hours from 1^st^ dose | 1 | 1 |  |
| <24 hours, 24 hours to 7 days, >7 days from 1^st^ dose | 1 | 3 |  |
| <24 hours, 24 to 47 hours, 48 hours to 7 days, >7 days from 1^st^ dose |  | 1 |  |
| <24 hours, 1 to 14 days from 1^st^ dose | 1 |  |  |
| <24, 24 to 47, 48 to 71, 72 to 95, 96 to 143, >144 hours from 1^st^dose |  | 1 |  |
| <24 hours, 24 to 48 hours, 48 hours to 7 days, >7 days from 1^st^ dose |  | 1 |  |
| <24 hours, 24 to <48 hours from 1^st^ dose |  | 1 |  |
| <24h, 24-48h, >48h from 1st dose |  |  | 1 |
| <24 hours, 24 to 48 hours, 2-7 days, >7 days from 1^st^ dose | 1 | 1 |  |
| 24 hours to 7 days, <24 hours or >7 days |  | 1 |  |
| 2 to 7 days, <48 hrs or >7 days from 1^st^dose |  | 1 |  |
| <24 hours, 48 hours to 7 days, >7days from 1^st^ dose |  | 1 |  |
| 1 to 7 days, >7 days | 1 |  |  |
| 1-6 days, 1-14 days, 1-21 days from 1^st^ dose | 1 |  |  |
| 1, 2, 3,4, 5, 6, 7 days from 1st dose |  |  | 1 |
| <48 hours, 48 hours to 7 days, >7 days from 1^st^ dose |  | 2 |  |
| <2 days, 2-7 days from 1^st^ dose |  | 1 |  |
| <2 days, 2 to 7 days, >7days from ACS (dose/course not specified) | 1 |  |  |
| Within 2 days, within 7 days from last dose |  | 1 |  |
| <2 days, 2 to 7 days, >7 days from 1^st^ dose |  | 3 |  |
| <2 days, 2 to <7 days, 7 to <14 days and 14 days from 1^st^ dose |  | 1 |  |
| ≤48 hours, >48 hours from 1^st^dose |  | 2 |  |
| >48 hours to <7 days, 7 to 14 days from 1^st^ dose |  | 1 |  |
| >48 hours to 14 days, >14 days from 1^st^ dose |  | 1 |  |
| <7 days, ≥7 days from completion of ACS course |  | 2 |  |
| ≤7 days, >7 days from 1^st^ dose |  | 4 |  |
| <7 days, 2 doses between 12h and 7 days of birth, 1 dose <12h from ACS |  | 1 |  |
| <7 days, 7 to 14 days, >14 days from 2nd dose |  | 1 |  |
| ≤14 days, >14 days from last dose |  | 1 |  |
