# Supplementary material for: Effect of antenatal corticosteroid administration-to-birth interval on maternal and newborn outcomes: a systematic review
Source: eClinicalMedicine. 2023 Mar 24;58:101916. doi: 10.1016/j.eclinm.2023.101916 (PMC10050784; doi:10.1016/j.eclinm.2023.101916)
Supplement: Tables S2–S20 [file mmc2.docx]

**Supplementary Table S2. Summary of findings on antenatal corticosteroid administration-to-birth interval and perinatal mortality (Randomized Controlled Trials)**

| **Study** | **Population** | **GA (weeks)** | **N** | **Time intervals** | **Perinatal mortality (n/N) %** | **Effect estimate** | **Narrative Summary^1^** | **Optimal ACS administration-to-birth interval** |
| --- | --- | --- | --- | --- | --- | --- | --- | --- |
| Dexiprom 1999 (1) | Singleton and multiple neonates of women with PPROM | 28-34 | 208 | No ACS | 10/103 (9.7%) | REF | - | None |
|  |  |  |  | <24 h | 3/30 (10.0%) | Too few events |  |  |
|  |  |  |  | >24 h | 1/75 (1.3%) | Too few events |  |  |
| WHO 2022 (2) | Women with singleton or multiple pregnancy and confirmed live fetus | 26-34 | 2904 | No ACS | Not reported | REF | Interval >7 d associated with decreased risk in perinatal mortality compared to no ACS. | >7 d |
|  |  |  |  | 0-6 h | Not reported | RR 1.11 (95% CI 0.87 – 1.41) |  |  |
|  |  |  |  | >6-12 h | Not reported | RR 1.00 (95% CI 0.72 – 1.40) |  |  |
|  |  |  |  | >12-24 h | Not reported | RR1.03 (95% CI 0.72 – 1.48) |  |  |
|  |  |  |  | >24 h – 7 d | Not reported | RR 0.83 (95% CI 0.68 – 1.00) |  |  |
|  |  |  |  | >7 d | Not reported | RR 0.67 (95% CI 0.47 – 0.94) |  |  |

§ Crude odds ratios and 95% confidence intervals calculated by review authors using data provided in published paper. NR: Not reported. The brief narrative summary is based on available effect estimates, and the author’s conclusions for that study

**Supplementary Table S3. Summary of findings on antenatal corticosteroid administration-to-birth interval and Perinatal mortality (Observational Studies)**

| **Study** | **Population** | **GA (weeks)** | **N** | **Time intervals** | **Perinatal Mortality (n/N) %** | **Effect estimate** | **Narrative Summary^1^** | **Optimal ACS administration-to-birth interval** |
| --- | --- | --- | --- | --- | --- | --- | --- | --- |
| Kosinska- Kaczynska 2016 (3) | Women with twin pregnancy | 26-34 | 211 | <7 d | 8/99 (8.1%) | REF | No difference in odds of perinatal mortality at interval ≥7 d compared to <7 d | None |
|  |  |  |  | ≥7 d | 6/112 (5.4%) | OR 0.64 (95% CI 0.22– 1.92) ^§^ |  |  |
| Hurrell 2022 (4) | Women delivering before 35 weeks' gestation with confirmed preeclampsia who were enrolled in the PELICAN study or PARROT trial | <35 | 250 | No ACS | 6/50 (12.0%) | REF | No difference in odds of perinatal mortality at >7 d compared to no ACS | None |
|  |  |  |  | ≤7 d | 3/78 (3.8%) | Too few events |  |  |
|  |  |  |  | >7 d | 5/122 (4.1%) | OR 0.31 (95% CI 0.09 – 1.08) ^§^ |  |  |
| McEvoy 2008 (5) | Singleton and multiple neonates with birthweight ≤2000g and without congenital anomalies | 25-32 | 56 | 1-7 d | 0/28 (0) | REF | **-** | None |
|  |  |  |  | >7 d | 1/28 (3.6%) | Too few events |  |  |
| Nagy 1978 (6) | Women with singleton or multiple pregnancy at risk of preterm birth | <37 | 460 | No ACS | 14/119 (11.8%) | REF | Interval >48 h was associated with decreased odds of perinatal mortality compared to no ACS | >48 h |
|  |  |  |  | <48 h | 24/247 (9.7%) | OR 0.81 (95% CI 0.40- 1.62) ^§^ |  |  |
|  |  |  |  | >48 h | 11/213 (5.2%) | OR 0.41 (95% CI 0.18-0.93) ^§^ |  |  |

§ Crude odds ratios and 95% confidence intervals calculated by review authors using data provided in published paper. The brief narrative summary is based on available effect estimates, and the author’s conclusions for that study

**Supplementary Table S4. Summary of findings on antenatal corticosteroid administration-to-birth interval and neonatal mortality (randomized controlled trials)**

| **Study** | **Population** | **GA (weeks)** | **N** | **Time intervals** | **Neonatal mortality (n/N) %** | **Effect estimate** | **Narrative Summary^1^** | **Optimal ACS administration-to-birth interval** |
| --- | --- | --- | --- | --- | --- | --- | --- | --- |
| Dexiprom 1999 (1) | Singleton and multiple neonates of women with PPROM | 28 - 34 | 208 | No ACS | 8/103 (7.6%) | REF | No difference in odds of neonatal mortality at <24 or >24 h compared to no ACS. | None |
|  |  |  |  | <24 h | 3/30 (10%) | OR 1.32 (95% CI 0.33 – 5.32) |  |  |
|  |  |  |  | >24 h | 1/75 (1.3%) | OR 0.16 (95% CI 0.02 – 1.31) |  |  |
| Kari 1994 **(7)** | Women with singleton or multiple pregnancies and threatened preterm birth with intact fetal membranes and no chorioamnionitis, insulin treated diabetes or fetal anomaly | 24 - 32 | 188 | No ACS | 6/88 (6.8%) | REF | No difference in odds of neonatal mortality at <24 h or 1-14 d compared to no ACS. | None |
|  |  |  |  | <24 h | 1/20 (5%) | OR 0.72 (95% CI 0.08 – 6.33) |  |  |
|  |  |  |  | 1-14 d | 2/41 (4.9%) | OR 0.70 (95% CI 0.14 – 3.63) |  |  |
| WHO 2022 (2) | Women with singleton or multiple pregnancy and confirmed live fetus | 26 - 34 | 2904 | No ACS | Not reported | REF | Interval of >24h – 7d was associated with decreased neonatal mortality compared to no ACS. | >24h – 7d |
|  |  |  |  | 0-6 h | Not reported | RR 1.08 (95% CI 0.83 – 1.41) |  |  |
|  |  |  |  | >6-12 h | Not reported | RR 0.89 (95% CI 0.63 – 1.28) |  |  |
|  |  |  |  | >12-24 h | Not reported | RR 1.02 (95% CI 0.67 – 1.56) |  |  |
|  |  |  |  | >24 h – 7 d | Not reported | RR 0.77 (95% CI 0.60 – 0.98) |  |  |
|  |  |  |  | >7 d | Not reported | RR 0.65 (95% CI 0.39 – 1.08) |  |  |

§ Crude odds ratios and 95% confidence intervals calculated by review authors using data provided in published paper. NR: Not reported. The brief narrative summary is based on available effect estimates, and the author’s conclusions for that study

**Supplementary Table S5. Summary of findings on antenatal corticosteroid administration-to-birth interval and neonatal mortality (observational studies)**

| **Study** | **Population** | **GA (weeks)** | **N** | **Time intervals** | **Neonatal mortality (n/N) %** | **Effect estimate** | **Narrative Summary^1^** | **Optimal ACS administration-to-birth interval** |
| --- | --- | --- | --- | --- | --- | --- | --- | --- |
| Arulalan 2022 (8) | Twin neonates | 28 – 34 | 536 | <14 d | Not reported | REF | Interval of >14 d was associated with an increased risk of neonatal mortality compared to <14 d | <14 d |
|  |  |  |  | >14 d | Not reported | aRR 2.0 (95% CI 1.03 – 3.88) |  |  |
| Barrett 1982 (9) | Women with singleton or multiple pregnancy with ruptured membranes and no chorioamnionitis, fetal distress or abrupt placentae | 26 - 34 | 93 | No ACS | 7/56 (12.6%) | REF | - | None |
|  |  |  |  | ≤24 h | 0/2 (0%) | Too few events |  |  |
|  |  |  |  | 24-47 h | 2/6 (33.3%) | Too few events |  |  |
|  |  |  |  | 48-71 h | 1/13 (7.6%) | Too few events |  |  |
|  |  |  |  | 72-95 h | 0/4 (0%) | Too few events |  |  |
|  |  |  |  | 96-143 h | 0/2 (0%) | Too few events |  |  |
|  |  |  |  | >144 h | 0/9 (0%) | Too few events |  |  |
| Battarbee 2020 (10) | Singleton neonates | 23 - 34 | 2259 | <2 d | 41/662 (6.6%) | REF | No difference in odds of neonatal mortality at any time interval compared to <2 d | None |
|  |  |  |  | 2 - <7 d | 59/821 (7.2%) | OR 1.10 (95% CI 0.73 – 1.66) ^§^ |  |  |
|  |  |  |  | 7 - <14 d | 31/401 (7.7%) | OR 1.19 (95% CI 0.73 – 1.93) ^§^ |  |  |
|  |  |  |  | >14 d | 21/415 (5.1%) | OR 0.76 (95% CI 0.44 – 1.30) ^§^ |  |  |
| Biedermann 2022 (11) | Neonates with a birthweight <1500g admitted to NICU | <34 | 239 | No ACS | 2/7 (28.6%) | REF | No difference in odds of neonatal mortality at <48 h or 2-7 d compared to no ACS | None |
|  |  |  |  | <48 h | 7/47 (14.9%) | OR 0.43 (95% CI 0.07 – 2.72) ^§^ |  |  |
|  |  |  |  | 2-7 d | 7/90 (7.8%) | OR 0.21 (95% CI 0.03 – 1.29) ^§^ |  |  |
|  |  |  |  | >7 d | 2/95 (2.1%) | Too few events |  |  |
| Chawla 2010 (12) | Singleton neonates with birthweight 401-1000g and without congenital anomalies | ≤28 | 169 | No ACS | 15/27 (55.5%) | REF | Intervals of 24 h – 7 d and >7 d were associated with decreased odds of neonatal mortality compared to no ACS. | 24 h – 7 d; <7 d |
|  |  |  |  | <24 h | 27/48 (56.3%) | OR 1.03 (95% CI 0.40 – 2.66) ^§^ |  |  |
|  |  |  |  | 24 h - 7 d | 16/53 (30.2%) | OR 0.35 (95% CI 0.13 – 0.9) ^§^ |  |  |
|  |  |  |  | >7 d | 6/41 (14.6%) | OR 0.14 (95% CI 0.04 – 0.43) ^§^ |  |  |
| Fortmann 2022 (13) | VLBW infants | 23 – 30 | 672 | No ACS | 19/238 (8.0%) | REF | No difference in odds of neonatal mortality at any time interval compared to no ACS | None |
|  |  |  |  | 0 – 24 h | 5/70 (7.1%) | aOR 0.77 (95% CI 0.24 – 2.5) |  |  |
|  |  |  |  | 24h – 7d | 9/187 (4.8%) | aOR 0.49 (95% CI 0.2 – 1.2) |  |  |
|  |  |  |  | >7 d | 8/177 (4.5%) | aOR 1.6 (95% CI 0.6 – 4.5) |  |  |
| Fuller 2017 (14) | Singleton neonates | 23 - 34 | 548 | No ACS | 4/58 (6.9%) | REF | No difference in odds of neonatal mortality at any time interval compared to no ACS | None |
|  |  |  |  | 1-23 h | 6/116 (5.2%) | OR 0.74 (95% CI 0.20 – 2.72) ^§^ |  |  |
|  |  |  |  | 24-47 h | 0/26 (0%) | Too few events |  |  |
|  |  |  |  | 2-7 d | 8/190 (4.2%) | OR 0.59 (95% CI 0.17 – 2.05) ^§^ |  |  |
|  |  |  |  | >7 d | 5/158 (3.2%) | Too few events |  |  |
| Gaur 2017 (15) | Women aged 18-45 with singleton or multiple pregnancy and without diabetes or other illness | <37 | 111 | <24 h | 8/71 (11.3%) | REF | - | None |
|  |  |  |  | >24 h | 2/40 (5.0%) | Too few events |  |  |
| Haas 2006 (16) | Neonates without congenital anomalies or fetal demise | 24 - 36 | 163 | <24 h | 9/113 (8.0%) | REF | - | None |
|  |  |  |  | 24 - 48 h | 1/50 (2.0%) | Too few events |  |  |
| Hurrell 2022 (4) | Women delivering before 35 weeks' gestation with confirmed preeclampsia who were enrolled in the PELICAN study or PARROT trial | <35 | 250 | No ACS | 1/50 (2.0%) | REF | - | None |
|  |  |  |  | ≤7 d | 2/78 (2.6%) | Too few events |  |  |
|  |  |  |  | >7 d | 4/122 (3.3%) | Too few events |  |  |
| Karmoker 2020 (17) | Singleton neonates without congenital anomalies | 24 - 34 | 200 | 2 d – <7 d | 0/140 (0%) | REF | - | None |
|  |  |  |  | 7-14 d | 1/60 (1.6%) | Too few events |  |  |
| Kuk 2013 (18) | Twin neonates | 23 - 34 | 468 | No ACS | 7/122 (5.7%) | REF | No difference in odds of neonatal mortality at <2d compared to no ACS | None |
|  |  |  |  | < 2 d | 7/166 (4.2%) | OR 0.72 (95% CI 0.25 – 2.12) ^§^ |  |  |
|  |  |  |  | 2-7 d | 1/114 (0.8%) | Too few events |  |  |
|  |  |  |  | >7 d | 0/66 (0%) | Too few events |  |  |
| Kyser 2012 (19) | Singleton or multiple neonates with birthweight 401-1000 g and without major anomalies admitted to the NICU | 22 - 25 | 237 | Any ACS exposure | 42/227 (18.5%) | REF | No difference in odds of neonatal mortality at any time interval compared to any ACS exposure. | None |
|  |  |  |  | <12 h | 15/67 (22.3%) | OR 1.27 (95% CI 0.65 – 2.47) ^§^ |  |  |
|  |  |  |  | 12 h – 7 d | 22/126 (17.5%) | OR 0.93 (95% CI 0.53 – 1.65) ^§^ |  |  |
|  |  |  |  | <7 d | 44/209 (21.0%) | OR 1.17 (95% CI 0.73 – 1.88) ^§^ |  |  |
| Liebowitz 2016 (20) | Singleton and multiple neonates without major anomalies admitted to the NICU | <28 | 667 | ≤6 h | 64/183 (35.0%) | REF | Interval of ≥24 h associated with decreased odds of neonatal mortality compared to ≤6 h. | ≥24 h |
|  |  |  |  | ≥24 h | 73/429 (17.0%) | aOR 0.25 (95%CI 0.13 – 0.47) |  |  |
| Li 2022 (21) | Neonates admitted to NICU | 24-32 | 706 | <24 h | 28/264 (10.6%) | aOR 2.8 (95% CI 1.1 – 6.8) | Interval of <24 h associated with increased odds of neonatal mortality compared to 2-7 d. | 1-2d, 2-7d and >7 d. |
|  |  |  |  | 1-2 d | 3/83 (3.6%) | aOR 1.5 (95% CI 0.4 – 6.1) |  |  |
|  |  |  |  | 2-7 d | 11/292 (3.8%) | REF |  |  |
|  |  |  |  | >7 d | 2/67 (3%) | aOR 1.3 (95% CI 0.2 – 7.1) |  |  |
| Madarek 2003 (22) | Women with singleton pregnancies giving birth preterm | 26 - 36 | 300 | No ACS | 32/150 (21.3%) | REF | Interval of <24 h associated with increased odds of neonatal mortality compared to no ACS. | None |
|  |  |  |  | < 24 h | 19/30 (63.3%) | OR 6.37 (95% CI 2.74-14.48) |  |  |
|  |  |  |  | 24-48 h | 8/30 (26.7%) | OR 1.34 (95% CI 0.55-3.29) |  |  |
|  |  |  |  | >48 h | 3/30 (10.0%) | Too few events |  |  |
| McEvoy 2008 (5) | Singleton and multiple neonates with birthweight ≤2000g and without congenital anomalies | 25 - 32 | 56 | 1-7 d | 0/28 (0%) | REF | - | None |
|  |  |  |  | >7 d | 1/28 (2.6%) | Too few events |  |  |
| Melamed 2015 (23) | Singleton live born neonates admitted to level III NICU | 24 - 34 | 6870 | No ACS | 107/1378 (7.8%) | aOR 2.56 (95%CI 1.83 – 3.59) | Interval of <24 h or >7 d was associated with increased odds of neonatal mortality compared to 1-7 d. | 1-7 d |
|  |  |  |  | <24 h | 86/1473 (6.0%) | aOR 1.59 (95%CI 1.16 – 2.18) |  |  |
|  |  |  |  | 1-7 d | 121/2721 (4.0%) | REF |  |  |
|  |  |  |  | >7 d | 50/1298 (4.0%) | aOR 1.40 (95% CI 1.00 – 1.97) |  |  |
| Nair 2009 (24) | Singleton neonates without congenital anomalies admitted to NICU | 24 - 28 | 163 | No ACS | 7/27 (25.9%) | REF | No difference in odds of neonatal mortality at <24 h compared to no ACS. | None |
|  |  |  |  | <24 h | 6/29 (20.7%) | OR 0.75 (95% CI 0.2 – 2.59) |  |  |
| Norberg 2017 (25) | Singleton and multiple neonates, including with congenital anomalies | 22 - 26 | 707 | No ACS | 52/85 (61.2%) | HR *survival* 0.22 (95% CI 0.12 – 0.38)  REF^§^ | Interval of <24 h was associated with increased odds of neonatal mortality compared to 48 h – 7 d.  No difference in odds of neonatal mortality at 24 – < 48 h and >7 d compared to 48 h – 7 d. | 48 h – 7 d |
|  |  |  |  | <24 h | 44/149 (29.5%) | HR *survival* 0.44 (95% CI 0.26 – 0.74)  OR mortality 0.27 (95% CI 0.15-0.47) ^§^ |  |  |
|  |  |  |  | 24 – <48 h | 10/66 (15.2%) | HR *survival* 2.08 (95% CI 0.8 – 5.47)  OR mortality 0.11 (95% CI 0.05-0.25) ^§^ |  |  |
|  |  |  |  | 48 h – 7 d | 28/171 (16.4%) | REF  OR mortality 0.12 (95% 0.07-0.23) ^§^ |  |  |
|  |  |  |  | >7 d | 18/103 (17.5%) | HR *survival* 0.54 (95% CI 0.29 – 1.03)  OR mortality 0.13 (0.07-0.26) ^§^ |  |  |
| Norman 2017 (26) | Singleton live births | 24 - 31 | 4594 | No ACS | 136/661 (20.6%) | REF | All time intervals were associated with decreased risk of neonatal mortality compared to no ACS. | None |
|  |  |  |  | <24 h | 117/1110 (10.5%) | aRR 0.6 (95%CI 0.5 – 0.7) |  |  |
|  |  |  |  | 24 h – 7 d | 171/1871 (9.1%) | aRR 0.5 (95%CI 0.4 – 0.6) |  |  |
|  |  |  |  | >7d | 89/950 (9.4%) | aRR 0.7 (95%CI 0.6 – 0.9) |  |  |
| Palas 2018 (27) | Twin neonates admitted to NICU | 24 - 31 | 750 | No ACS | 57/595 (9.6%) | REF | Interval of ≤7 d was associated with decreased odds of neonatal mortality, compared to no ACS. | ≤7 d |
|  |  |  |  | ≤7 d | 22/272 (8.0%) | aOR 0.27 (95% CI 0.12 – 0.62) |  |  |
|  |  |  |  | >7 d | 15/230 (6.5%) | aOR 0.71 (95% CI 0.27 – 1.84) |  |  |
| Peaceman 2005 (28) | Single and multiple neonates | 26 - 34 | 197 | ≤7 d | 2/99 (2.0%) | REF | - | None |
|  |  |  |  | >7 d | 0/98 (0%) | Too few events |  |  |
| Ryu 2019 (29) | Singleton preterm neonates born to women with and without histological chrioamnionitis | 23 - 34 | 254 | No ACS | 4/35 (11.4%) | REF | No difference in odds of neonatal mortality at any time interval compared to no ACS | None |
|  |  |  |  | 2-7 d | 2/91 (2.2%) | OR 0.17 (95% CI 0.03 – 1.00) |  |  |
|  |  |  |  | <48 h or >7 d | 5/128 (3.9%) | OR 0.32 (95% CI 0.08 – 1.24) |  |  |
| Schmidt 2011 (30) | Singleton and multiple neonates birth weight 500-999g | NR | 1195 | No ACS | Placebo for >24 hours exposure: 45/331 (13.6%)  Placebo for <24 hours exposure: 66/261 (25.3) | REF | No difference in odds of neonatal mortality at any time interval compared to no ACS | None |
|  |  |  |  | >24 h | 60/332 (18.1%) | OR 1.40 (95% CI 0.92 – 2.14) |  |  |
|  |  |  |  | <24 h | 64/258 (24.8%) | OR 0.97 (95% CI 0.66 – 1.45) |  |  |
| Sehdev 2004 (31) | Singleton neonates with birth weight 500-1500g born to women admitted for preterm labour, PROM, or indicated for labour (chorioamnionitis, non-reassuring fetal testing) | <28 | 325 | <24 h | 4/44 (9.1%) | OR 0.73 (95% CI 0.05 – 11.75) | No difference in odds of neonatal mortality at any time interval compared to 48 h - 7 d. | None |
|  |  |  |  | 24-48 h | 6/95 (6.3%) | OR 0.97 (95% CI 0.12 – 8.15) |  |  |
|  |  |  |  | 48 h - 7 d | 5/106 (4.7%) | REF |  |  |
|  |  |  |  | >7 d | 2 (2.5%) | OR 2.21 (95% CI 0.06 – 85.40) |  |  |
| Sen 2002 (32) | Singleton and multiple neonates admitted to NICU having received surfactant within first 2hrs of life | <31 | 226 | No ACS | 28/89 (31.5%) | REF | Intervals of 4-24 h and 24 h - 7 d were associated with decreased odds of neonatal mortality compared to no ACS.  No difference in odds of neonatal mortality at 4-24 h compared to 24 h – 7 d. | None |
|  |  |  |  | 4-24 h | 6/69 (8.6%) | 4-24 h vs no ACS: RR 0.28 (95% CI 0.12 – 0.63)  4-24 h vs 24 h-7 d: RR 1.18 (95% CI 0.38 – 3.69) |  |  |
|  |  |  |  | 24 h - 7 d | 5/68 (7.4%) | 24 h-7 d vs no ACS: RR 0.23 (95% CI 0.10 – 0.57) |  |  |
| Siegler 2022 (33) | Singleton neonates | 24 – 34 | 327 | <2 d | 7/200 (3.5%) | REF | No difference in odds of neonatal mortality at 2-7 d compared <2 d. | None |
|  |  |  |  | 2-7 d | 9/172 (5.2%) | OR 1.52 (95% CI 0.55 – 4.18) *^§^* |  |  |
| Wong 2014 (34) | Singleton or multiple neonates without congenital anomalies admitted to NICU | <29 | 2549 | No ACS | 96/319 (30.1%) | REF | Interval of 48 h - 7 d was associated with decreased odds of neonatal mortality compared to <24 h or no ACS. | 48 h – 7 d |
|  |  |  |  | <24 h | 184/677 (27.2%) | OR 0.87 (95% CI 0.65 – 1.16) |  |  |
|  |  |  |  | 48-7 d | 214/1281 (16.7%) | OR 0.47 (95% CI 0.35 – 0.62) |  |  |
|  |  |  |  | No ACS/<24 h | 280/996 (28.1%) | REF (for 48 h-7 d/>7 d comparison) |  |  |
|  |  |  |  | 48 h-7 d/>7 d | 266/1553 (17.1%) | OR 0.53 (95% CI 0.44 – 0.64) |  |  |

§ Crude odds ratios and 95% confidence intervals calculated by review authors using data provided in published paper. NR: Not reported. The brief narrative summary is based on available effect estimates, and the author’s conclusions for that study

**Supplementary Table S6. Summary of findings on antenatal corticosteroid administration-to-birth interval and Respiratory Distress Syndrome (RDS) (randomized controlled trials)**

| **Study** | **Population** | **GA (weeks)** | **N** | **Time intervals** | **RDS (n/N) %** | **Effect estimate** | **Narrative Summary^1^** | **Optimal ACS administration-to-birth interval** |
| --- | --- | --- | --- | --- | --- | --- | --- | --- |
| Anonymous  1981 (35) | Women with singleton or multiple pregnancy at high risk of preterm labour | 26 - 37 | 696 | No ACS | 65/359 (18.1%) | REF | Intervals of 24 h – 7 d and >7 d were associated with decreased odds RDS compared to no ACS. | 24 h -7d and >7 d |
|  |  |  |  | <24 h | 11/56 (19.6%) | OR 1.11(95% CI 0.54 – 2.26) ^§^ |  |  |
|  |  |  |  | 24 h-7 d | 14/151 (9.3%) | OR 0.46 (95% CI 0.25 – 0.86) ^§^ |  |  |
|  |  |  |  | >7 d | 6/100 (6.0%) | OR 0.29 (95% CI 0.12 – 0.69) ^§^ |  |  |
| Block  1977 (36) | Singleton neonates | NR | 128 | No ACS | 12/44 (27.3%) | REF | - | None |
|  |  |  |  | ≤24 h | 1/13 (7.7%) | Too few events |  |  |
|  |  |  |  | >24 h | 4/36 (11.1%) | Too few events |  |  |
| Dexiprom  1999 (1) | Singleton and multiple neonates of women with preterm premature rupture of membranes. | 28 - 24 | 208 | No ACS | 27/103 (26.2%) | REF | No difference in odds of RDS at any time interval compared to no ACS. | None |
|  |  |  |  | <24 h | 13/30 (43.3%) | OR 2.15 (95% CI 0.92 – 5.01) ^§^ |  |  |
|  |  |  |  | >24 h | 19/75 (25.3%) | OR 0.96 (05% CI 0.48 – 1.89) ^§^ |  |  |
| Gamsu  1989 (37) | Women with singleton or multiple pregnancy in spontaneous preterm labour or with complications requiring preterm birth | <34 | 262 | No ACS | 16/132 (12.1%) | REF | - | None |
|  |  |  |  | 1-6 d | 0/30 (0%) | Too few events |  |  |
|  |  |  |  | 1-14 d | 1/44 (2.3%) | Too few events |  |  |
|  |  |  |  | 1-21 d | 3/51 (5.9%) | Too few events |  |  |
| Kari  1994 (7) | Women with singleton or multiple pregnancies and threatened preterm birth with intact fetal membranes and no chorioamnionitis, insulin treated diabetes or fetal anomaly | 24 - 32 | 188 | No ACS | 45/88 (51.1%) | REF | No difference in odds of RDS at any time interval compared to no ACS. | None |
|  |  |  |  | <24 h | 11/20 (55.0%) | OR 1.17 (95% CI 0.44 – 3.10) |  |  |
|  |  |  |  | 1-14 d | 18/41 (91.7%) | OR 0.75 (95% CI 0.35 – 1.58) |  |  |
| Liggins 1972 (38) | Women with singleton or multiple pregnancy and threatened or planned (due to obstetric complications) preterm labour | 24 - 36 | 282 maternal | No ACS | 25/97 (25.7%) | REF | No difference in odds of RDS at <24 h compared to no ACS. | None |
|  |  |  |  | <24 h | 7/29 (24.1%) | OR 0.92 (95% CI 0.35 – 2.40) ^§^ |  |  |
|  |  |  |  | 24-48 h | 2/20 (10.0%) | Too few events |  |  |
|  |  |  |  | 2-7 d | 1/28 (3.6%) | Too few events |  |  |
|  |  |  |  | >7 d | 1/45 (2.2%) | Too few events |  |  |
| Luerti 1987 (39) | Women with singleton or multiple pregnancies and threatened or planned (due to obstetric complications) preterm labour | 27 - 34 | 315 | <2 d | 5/16 (31.3%) | REF | No difference in odds of RDS at any time interval compared to no ACS | None |
|  |  |  |  | 2 – 7 d | 13/35 (37.1%) | OR 1.30 (95% CI 0.37 – 4.58) ^§^ |  |  |
|  |  |  |  | >7 d | 9/35 (25.7%) | OR 0.76 (95% CI 0.21 – 2.80) ^§^ |  |  |
| Schutte 1980 (40) | Women with singleton or multiple pregnancies with threatened preterm labour | 26 - 32 | 95 | No ACS | 17/46 (37.0%) | REF | No difference in odds of RDS at any time interval compared to no ACS. | None |
|  |  |  |  | <12 h | 5/10 (50.0%) | OR 1.71 (95% CI 0.43 – 6.76) ^§^ |  |  |
|  |  |  |  | 12 h – 7 d | 0/22 (0%) | Too few events |  |  |
|  |  |  |  | 8-21 d | 1/9 (11.1%) | Too few events |  |  |
|  |  |  |  | >21d | 5/8 (62.5%) | OR 2.84 (95% CI 0.06 – 13.42) ^§^ |  |  |
| Teramo 1980 (41) | Women with singleton or multiple pregnancies | 28 - 36 | 80 | No ACS | 3/42 (7.1% | REF | - | None |
|  |  |  |  | <24 h | 2/11 (18.2%) | Too few events |  |  |
|  |  |  |  | 1-7 d | 1/21 (4.8%) | Too few events |  |  |
|  |  |  |  | >7 d | 0/6 (0%) | Too few events |  |  |

§ Crude odds ratios and 95% confidence intervals calculated by review authors using data provided in published paper. NR: Not reported. The brief narrative summary is based on available effect estimates, and the author’s conclusions for that study

**Supplementary Table S7. Summary of findings on antenatal corticosteroid administration-to-birth interval and Respiratory Distress Syndrome (RDS) (observational studies)**

| **Study** | **Population** | **GA (weeks)** | **N** | **Time intervals** | **RDS (n/N) %** | **Effect estimate** | **Narrative Summary** | **Optimal ACS administration-to-birth interval** |
| --- | --- | --- | --- | --- | --- | --- | --- | --- |
| Arulalan 2022 (8) | Twin neonates | 28 – 34 | 536 | <14 d | Not reported | REF | No difference in risk of RDS at >14 d compared to <14 d. | None |
|  |  |  |  | >14 d | Not reported | aRR 1.13 (95% CI 0.93 – 1.33) |  |  |
| Asl et al  2005 (42) | Neonates born at 30-36 weeks | 30 - 36 | 170 | No ACS | 17/70 (21.4%) | REF | No difference in odds of RDS at <24 h compared to no ACS. | None |
|  |  |  |  | <24 h | 19/100 (19.0%) | OR 0.86 (95% CI 0.4-1.84)*^§^* |  |  |
| Battarbee  2020 (10) | Singleton neonates | 23 - 34 | 2259 | <2 d | 390/622 (62.7%) | OR 2.07 (95% CI 1.61-2.66) | Intervals <2 d, 7 - <14 d and ≤14 d were associated with increased odds of RDS compared to 2 - <7 d. | 2 - <7 d |
|  |  |  |  | 2-<7 d | 421/821 (51.3%) | REF |  |  |
|  |  |  |  | 7-<14 d | 224/401 (55.9%) | OR 1.40 (95% CI 1.07 – 1.83) |  |  |
|  |  |  |  | ≤14 d | 239/415 (57.6%) | OR 2.38 (95% CI 1.78 – 3.07) |  |  |
| Caspi  1976 (43) | Threatened preterm delivery | 28 - 36 | 126 | No ACS | 25/71 (35.2%) | REF | - | None |
|  |  |  |  | 1 d | 0/2 (0%) | Too few events |  |  |
|  |  |  |  | 2 d | 1/4 (25.0%) | Too few events |  |  |
|  |  |  |  | 3 d | 3/6 (50.0%) | Too few events |  |  |
|  |  |  |  | 4 d | 1/15 (6.7%) | Too few events |  |  |
|  |  |  |  | 5 d | 0/2 (0%) | Too few events |  |  |
|  |  |  |  | 6 d | 0/4 (0%) | Too few events |  |  |
|  |  |  |  | 7 d | 0/22 (0%) | Too few events |  |  |
| Ferguson  2009 (44) | Women with singleton pregnancy and severe hypertension of pregnancy | 26 - 34 | 172 | ≤48 h | 31/55 (56.4%) | REF | No difference in odds of RDS at >48 h compared to ≤48 h. | None |
|  |  |  |  | >48 h | 50/117 (42.7%) | OR 0.58 (95% CI 0.3 – 1.1)*^§^* |  |  |
| Frandberg  2018 (45) | Preterm neonates admitted to the NICU | 23 - 34 | 498 | No ACS | NR | OR 4.62 (95% CI 1.28 – 16.66) | Intervals of 0 – 24 h, >7 d and no ACS were associated with increased odds of RDS compared to 24 h – 7 d. | 24 h - 7 d |
|  |  |  |  | 0-24 h | NR | OR 2.16 (95% CI 1.11 – 4.22) |  |  |
|  |  |  |  | 24h-7 d | NR | REF |  |  |
|  |  |  |  | > 7 d | NR | OR 2.00 (95% CI 1.05 – 3.79) |  |  |
| Fuller 2017 (14) | Singleton neonates | 23 - 34 | 548 | No ACS | 39/58 (67.2%) | REF | Intervals of 24 - <2 d and >7 d associated with decreased odds of RDS compared to no ACS. | 24 h - <2 d; >7 d |
|  |  |  |  | 1- <24 h | 64/116 (55.2%) | OR 0.6 (95% CI 0.3-1.19) |  |  |
|  |  |  |  | 24 h - <2 d | 10/26 (38.5%) | OR 0.28 (95% CI 0.11 – 0.74) |  |  |
|  |  |  |  | 2 - 7 d | 106/190 (55.8%) | OR 0.6 (95% CI 0.31-1.14) |  |  |
|  |  |  |  | >7 d | 79/158 (50.0%) | OR 0.49 (95% CI 0.26-0.95) |  |  |
| Gaur 2017 (15) | Women aged 18-45 with singleton or multiple pregnancy and without diabetes or other illness | <37 | 111 | <24 h | 12/71 (16.9%) | REF | - | None |
|  |  |  |  | >24 h | 2/40 (5.0%) | Too few events |  |  |
| Gulersen  2021 (46) | Singleton neonates | 34 - 37 | 1248 | <2 d | 74/772 (9.6%) | REF | - | None |
|  |  |  |  | 2-7 d | 1/168 (0.6%) | Too few events |  |  |
|  |  |  |  | >7 d | 2/308 (0.7%) | Too few events |  |  |
| Guruvare  2015 (47) | Singleton neonates | 28 - 34 | 284 | 0-7 d | 35/97 (36.1%) | REF | Intervals of 22 – 28 d and >29 d were associated with decreased odds of RDS compared to 0 – 7 d. | 22-28 d; >29 d |
|  |  |  |  | 8-14 d | 10/29 (34.5%) | OR 0.87 (95% CI 0.33 – 2.29) |  |  |
|  |  |  |  | 15-21 d | 4/16 (25.0%) | OR 1.79 (95% CI 0.48 – 6.5) |  |  |
|  |  |  |  | 22-28 d | 4/19 (21.1%) | OR 0.16 (95% CI 0.03 – 0.83) |  |  |
|  |  |  |  | >29 d | 6/42 (14.3%) | OR 0.01 (95% CI 0.001 - 0.03) |  |  |
| Haas 2006 (16) | Singleton neonates without congenital anomalies or fetal demise | 24 - 36 | 163 | <24 h | 74/113 (65.5%) | REF | Interval of 24 - <48 h was associated with decreased odds of RDS compared to <24 h. | 24 - <48 h |
|  |  |  |  | 24 – <48 h | 19/50 (38.0%) | OR 0.32 (95% CI 0.16 – 0.64)*^§^* |  |  |
| Hurrell 2022 (4) | Women delivering before 35 weeks' gestation with confirmed preeclampsia who were enrolled in the PELICAN study or PARROT trial | <35 | 250 | No ACS | 16/50 (32.0%) | REF | Interval of >7 d was associated with increased odds of RDS compared to no ACS. | None |
|  |  |  |  | ≤7 d | 37/78 (47.4%) | OR 1.92 (95% CI 0.91 – 4.03) *^§^* |  |  |
|  |  |  |  | >7 d | 60/122 (49.2%) | OR 2.06 (95% CI 1.03 – 4.11) *^§^* |  |  |
| Janssen 2021 (48) | Preterm neonates delivered between 34- and 37-weeks’ gestation | 34^0/7^-35^6/7^ (immature group)  36^0/7^-36^6/7^ (mature group) | 500 | No ACS (immature) | Not reported | REF | Intervals of within 2 d and within 7 d were associated with decreased odds of RDS compared to no ACS, in the immature group.  No difference in odds of RDS at any time interval compared to no ACS in the mature group. | Within 2 and 7 d (immature group). |
|  |  |  |  | Within 2 d (immature) | Not reported | aOR 0.37 (95% CI 0.17 – 0.78) |  |  |
|  |  |  |  | Within 7 d (immature) | Not reported | aOR 0.42 (95% CI 0.20 – 0.88) |  |  |
|  |  |  |  | No ACS (mature) | Not reported | REF |  |  |
|  |  |  |  | Within 2 d (mature) | Not reported | aOR 1.63 (95% CI 0.60 – 4.39) |  |  |
|  |  |  |  | Within 7 d (mature) | Not reported | aOR 1.41 (95% CI 0.54 – 3.64) |  |  |
| Karmoker 2020 (17) | Singleton neonates without congenital anomalies | 24 - 34 | 200 | 2 d – <7 d | 105/140 (75.0%) | REF | Interval of 7-14 d was associated with increased odds of RDS compared to 48 h – <7 d. | 7-14 d |
|  |  |  |  | 7-14 d | 55/60 (91.7%) | OR 3.67 (95%CI 1.36 – 9.89)*^§^* |  |  |
| Kosinska- Kaczynska 2016 (3) | Women with twin pregnancy | 26 - 34 | 211 | <7 d | 32/99 (32.3%) | REF | No difference in odds of RDS at ≥ 7 d compared to <7 d. | None |
|  |  |  |  | ≥ 7 d | 44/112 (39.3%) | OR 1.35 (95% CI 0.77-2.39)*^§^* |  |  |
| Kuk 2013 (18) | Twin neonates | 23 - 34 | 468 | No ACS | 86/166 (51.8%) | REF | Interval of 2-7 d was associated with decreased odds of RDS compared to no ACS. | 2-7 d |
|  |  |  |  | <2 d | 38/144 (33.3%) | OR 1.04 (95% CI 0.65 – 1.66)*^§^* |  |  |
|  |  |  |  | 2 – 7 d | 26/66 (39.4%) | OR 0.48 (95% CI 0.29 – 0.82)*^§^* |  |  |
|  |  |  |  | >7 d | 62/122 (50.8%) | OR 0.63 (95% CI 0.34-1.16)*^§^* |  |  |
| Lau 2017 (49) | Singleton and multiple neonates | 23^5^ - 37 | 352 | <2 d | 21/99 (21.2%) | aOR 0.53 (95% CI 0.17-1.72) | Interval of >7 d associated with increased odds of RDS compared to no ACS. | 2-7 d |
|  |  |  |  | 2 -7 d | 17/51 (33.3%) | REF |  |  |
|  |  |  |  | >7 d | 12/66 (18.2%) | aOR 7.02 (95% CI 1.54-32.07) |  |  |
| Li 2022 (21) | Neonates admitted to NICU | 24-32 | 706 | <24 h | 174/264 (65.9%) | aOR 1.8 (95% CI 1.2 – 2.7) | Interval of <24 h associated with increased odds of RDS, compared to 2-7 d. | 1-2 d, 2-7 d and >7d |
|  |  |  |  | 1-2 d | 40/83 (48.2%) | aOR 1.4 (95% CI 0.8 – 2.4) |  |  |
|  |  |  |  | 2-7 d | 133/292 (45.6%) | REF |  |  |
|  |  |  |  | >7 d | 29/67 (43.3%) | aOR 0.8 (95% CI 0.4 – 1.4) |  |  |
| McEvoy 2008 (5) | Singleton and multiple neonates with birthweight ≤2000g and without congenital anomalies | 25 - 32 | 56 | 1-7 d | 5/28 (17.8%) | REF | Interval of >14 d associated with increased odds of RDS compared to 1 – 7d. | 1-7 d |
|  |  |  |  | >7 d | 11/28 (39.2%) | OR 2.98 (95% CI 0.87-10.17)*^§^* |  |  |
|  |  |  |  | 8-14 d | 2/10 (20.0%) | Too few events |  |  |
|  |  |  |  | >14 d | 9/18 (50.0%) | OR 4.60 (95% CI 1.21- 17.52)*^§^* |  |  |
| Nagy 1978 (6) | Women with singleton or multiple pregnancy at risk of preterm birth | <37 | 460 | No ACS | 32/119 (27.0%) | REF | Interval of >48 h associated with decreased odds of RDS compared to no ACS. | >48 h |
|  |  |  |  | <48 h | 69/247 (27.9%) | OR 1.05 (95% CI 0.64- 1.72)*^§^* |  |  |
|  |  |  |  | >48 h | 29/213 (13.6%) | OR 0.43 (95% CI 0.24-0.75)*^§^* |  |  |
| Nair 2009 (24) | Singleton neonates without congenital anomalies admitted to NICU | 24 - 28 | 163 | No ACS | 20/27 (74.1%) | REF | No difference in odds of RDS at <24 h compared to no ACS. | None |
|  |  |  |  | <24 h | 23/29 (79.3%) | OR 1.34 (95% CI 0.39- 4.66)*^§^* |  |  |
| Ryu 2019 (29) | Singleton preterm neonates born to women with and without histological chorioamnionitis | 23 - 34 | 254 | No ACS | 13/35 (37.1%) | REF | No difference in odds of RDS at 2 -7 d and <48 h or > 7 d compared to no ACS. | None |
|  |  |  |  | 2-7 d | 19/91 (20.9%) | OR 0.45 (95% CI 0.19-1.05)*^§^* |  |  |
|  |  |  |  | <48 h or >7 d | 33/128 (25.8%) | OR 0.59 (95% CI 0.27- 1.3)*^§^* |  |  |
| Sehdev 2004 (31) | Singleton neonates with birthweight 500-1500g born to women admitted for preterm labour, PROM, or indicated for labour (chorioamnionitis, non-reassuring fetal testing) | <28 | 325 | <24 h | 21/44 (47.7%) | REF | No difference in odds of RDS at any interval compared to <24 h. | None. |
|  |  |  |  | 24-48 h | 43/95 (45.3%) | OR 0.91 (95% CI 0.44-1.85)*^§^* |  |  |
|  |  |  |  | 48 h -7 d | 53/106 (50.0%) | OR 1.1 (95% CI 0.54-2.21 )*^§^* |  |  |
|  |  |  |  | >7 d | 38/80 (47.5%) | OR 0.44 (95% CI 0.12-1.63)*^§^* |  |  |
| Sekhavat 2011 (50) | Singleton neonates | 28 - 34 | 104 | < 2 d | 25/29 (86.2%) | REF | Interval of 2 - 7 d associated with decreased odds of RDS compared to <2 d. | 2-7 d |
|  |  |  |  | 2-7 d | 21/41 (51.2%) | OR 0.17 (95% CI 0.05-0.57)*^§^* |  |  |
|  |  |  |  | >7 d | 25/34 (73.5%) | OR 0.44 (95% CI 0.12-1.63)*^§^* |  |  |
| Siegler 2022 (33) | Singleton neonates | 24 – 34 | 327 | <2 d | 76/200 (38.0%) | REF | Interval of 2 - 7 d associated with decreased odds of RDS compared to <2 d. | 2-7 d |
|  |  |  |  | 2-7 d | 36/172 (20.9%) | OR 0.43 (95% CI 0.27 – 0.69) *^§^* |  |  |
| Tomotaki 2021 (51) | VLBW neonates | <30 | 115 | No ACS or <24 h | 31/36 (86.1%) | REF | Interval of 1-7 d associated with reduced odds of RDS compared to no ACS or <24 h. | 1-7 d |
|  |  |  |  | 1-7 d | 25/41 (61.0%) | OR 0.25 (95% CI 0.08 – 0.78) *^§^* |  |  |
|  |  |  |  | >8 d | 27/38 (71.1%) | OR 0.40 (95% CI 0.12 – 1.28) *^§^* |  |  |
| Vermillion 2001 (52) | Women with singleton pregnancy, intact membranes and no fetal anomalies | 28 - 34 | 216 | 1-2 d | 38/97 (39.1%) | REF | No difference in odds of RDS at any time interval compared to 1-2 d. | None |
|  |  |  |  | 3-7 d | 32/78 (41.0%) | OR 1.08 (95% CI 0.59-1.98)*^§^* |  |  |
|  |  |  |  | 8-14 d | 15/41 (36.6%) | OR 0.9 (95% CI 0.42-1.91)*^§^* |  |  |
| Waters 2009 (53) | Singleton neonates without congenital anomalies. | 30 - 34 | 524 | 2-7 d | 68/120 (56.7%) | REF | Interval of >7 d associated with increased odds of RDS compared to 2-7 d. | 2-7 d |
|  |  |  |  | >7 d | 91/131 (69.5%) | OR 1.74 (95% CI 1.04-2.92)*^§^* |  |  |
| Wilms 2011 (54) | Singleton and multiple neonates | <34 | 254 | 0-7 d | 55/147 (37.4%) | REF | No difference in odds of RDS at any time interval compared to 0-7 d. | None |
|  |  |  |  | 8-14 d | 17/62 (27.4%) | OR 0.63 (95% CI 0.33-1.21)*^§^* |  |  |
|  |  |  |  | 15-21 d | 9/23 (39.1%) | OR 1.08 (95% CI 0.44-2.65)*^§^* |  |  |
|  |  |  |  | 22-28 d | 2/9 (22.2%) | Too few events |  |  |
| Yasuhi 2017 (55) | Women with singleton pregnancy with no fetal anomalies | 24 - 33 | 397 | <7 d | 20/83 (24.1%) | REF | Interval of 7-14 d and >14 d associated with increased odds of RDS compared to <7 d. | <7 d |
|  |  |  |  | 7-14 d | 5/14 (35.7%) | aOR 12.8 (95% CI 1.31-164.7) |  |  |
|  |  |  |  | >14 d | 9/20 (45.0%) | aOR 64.0 (95% CI 1.32-5808.6) |  |  |

§ Crude odds ratios and 95% confidence intervals calculated by review authors using data provided in published paper. NR: Not reported. Where OR was reported by original study authors in groups with less than 5 events, we have included these ORs. The brief narrative summary is based on available effect estimates, and the author’s conclusions for that study

**Supplementary Table S8. Summary of findings on antenatal corticosteroid administration-to-birth interval and Intraventricular haemorrhage (IVH) (randomized controlled trials)**

| **Study** | **Population** | **GA (weeks)** | **N** | **Time intervals** | **IVH (n/N) %** | **Effect estimate** | **Narrative Summary** | **Optimal ACS administration- to-birth interval** |
| --- | --- | --- | --- | --- | --- | --- | --- | --- |
| Kari (1994) (7) | Preterm neonates with absence of congenital fetal anomalies detectable by ultrasound or documented fetal lung maturity | 24 - 32 | 188 | No ACS | 18/64 | REF | - | None |
|  |  |  |  | <24 h | 4/20 | Too few events |  |  |
|  |  |  |  | 1-14 d | 3/41 | Too few events |  |  |

**Supplementary Table S9. Summary of findings on antenatal corticosteroid administration-to-birth interval and Intraventricular haemorrhage (IVH) (observational studies)**

| **Study** | **Population** | **GA (weeks)** | **N** | **Time intervals** | **IVH (n/N) %** | **Effect estimate** | **Narrative Summary^1^** | **Optimal ACS administration- to-birth interval** |
| --- | --- | --- | --- | --- | --- | --- | --- | --- |
| Battarbee 2020 (10) | Singleton neonates | 23 - 34 | 2259 | <2 d | 42/622 | REF | All time intervals were associated with decreased odds of IVH compared to <2 d. | 2-<7 d, 7-<14d, ≥ 14 d |
|  |  |  |  | 2 - <7 d | 20/821 | OR 0.34 (95% CI 0.20 – 0.59)^§^ |  |  |
|  |  |  |  | 7 - <14 d | 11/401 | OR 0.39 (95% CI 0.20 – 0.77)^§^ |  |  |
|  |  |  |  | ≥ 14 d | 11/415 | OR 0.38 (95% CI 0.19 – 0.74)^§^ |  |  |
| Chawla 2019 (12) | Singleton neonates with birthweight 401-1000g and without congenital anomalies | ≤28 | 169 | No ACS | 18/26 | REF | All time intervals were associated with decreased odds of IVH compared to no ACS. | <24 h, 24 h – 7 d, >7 d |
|  |  |  |  | <24 | 18/47 | OR 0.28 (95% CI 0.10 – 0.76)^§^ |  |  |
|  |  |  |  | 24 h - 7 d | 5/50 | OR 0.05 (95% CI 0.01 – 0.17)^§^ |  |  |
|  |  |  |  | >7 d | 12/40 | OR 0.19 (95% CI 0.07 – 0.56)^§^ |  |  |
| Fortmann 2022 (13) | VLBW infants | 23 – 30 | 672 | No ACS | 34/238 (14.3%) | REF | Intervals of 24h – 7d and >7 d were associated with decreased odds of IVH compared to no ACS. | 24 h – 7 d, >7 d |
|  |  |  |  | 0 – 24 h | 9/70 (12.9%) | OR 0.89 (95% CI 0.40 – 1.95) ^§^ |  |  |
|  |  |  |  | 24h – 7d | 7/187 (3.7%) | OR 0.23 (95% CI 0.10 – 0.54) ^§^ |  |  |
|  |  |  |  | >7 d | 10/177 (5.6%) | OR 0.36 (95% CI 0.17 – 0.75) ^§^ |  |  |
| Fuller 2017 (14) | Singleton neonates | 23 - 34 | 548 | No ACS | 7/58 | REF | Interval of >7 d was associated with decreased odds of IVH compared to no ACS. | >7 d |
|  |  |  |  | 1-23 h | 13/116 | OR 0.92 (95% CI 0.35 - 2.45) |  |  |
|  |  |  |  | 24-47 h | 0/26 | Too few events |  |  |
|  |  |  |  | 2-7 d | 13/190 | OR 0.54 (95%CI 0.20 – 1.41) |  |  |
|  |  |  |  | >7 d | 6/158 | OR 0.29 (95% CI 0.09 – 0.90) |  |  |
| Haas 2006 (16) | Singleton neonates without congenital anomalies or fetal demise | 24 - 36 | 166 | <24 h | 14/113 | REF | - | None |
|  |  |  |  | 24 - <48 h | 3/50 | Too few events |  |  |
| Karmoker 2020 ^(17)^ | Singleton neonates without congenital anomalies | 24 - 34 | 200 | >2 d - <7 d | 2/140 | Too few evenst | - | None |
|  |  |  |  | 7-14 d | 0/60 | Too few events |  |  |
| Kosinska-Kaczynska 2016 (3) | Women with twin pregnancy | 26 - 34 | 211 | <7 d | 27/99 | REF | No difference in odds of IVH at ≥7 d compared to <7 d | None |
|  |  |  |  | ≥7 d | 38/112 | OR 1.37 (95% CI 0.76 – 2.47) ^§^ |  |  |
| Kuk 2013 (18) | Twin neonates | 24 - 34 | 468 | No ACS | 7/122 | REF | - | None |
|  |  |  |  | <2 d | 3/166 | Too few events |  |  |
|  |  |  |  | 2-7 d | 3/114 | Too few events |  |  |
|  |  |  |  | >7 d | 0/66 | Too few events |  |  |
| Liebowitz 2016 (20) | Singleton and multiple neonates without major anomalies admitted to the NICU. | <28 | 667 | <6 h | 61/175 | REF | Intervals 7-23h, ≥24 h, 2-7 d and ≥10 d were associated with decreased odds of IVH compared to <6 h | 7-23h, ≥24 h, 2-7 d and ≥10 d |
|  |  |  |  | 7 h-23 h | 10/53 | OR 0.43 (95%CI 0.20 – 0.92) ^§^ |  |  |
|  |  |  |  | ≥24 h | 37/411 | OR 0.18 (95%CI 0.12 – 0.29) ^§^ |  |  |
|  |  |  |  | 24-47 h | 1/45 | Too few events |  |  |
|  |  |  |  | 48 h-7 d | 18/231 | OR 0.15 (95% CI 0.08 – 0.27) ^§^ |  |  |
|  |  |  |  | 8 d-9 d | 2/21 | Too few events |  |  |
|  |  |  |  | ≥10 d | 13/77 | OR 0.38 (95% CI 0.19 – 0.74) ^§^ |  |  |
|  |  |  |  | 8-9 d | 2/21 | Too few events |  |  |
| Melamed 2015 (23) | Singleton live-born neonates admitted to level III NICU | 24 - 34 | 6870 | No ACS | 161/1378 | REF | Intervals of 1-7 d and >7 d were associated with decreased odds of IVH compared to no ACS. | 1-7 d; >7 d |
|  |  |  |  | <24 h | 148/1473 | OR 0.84 (95% CI 0.67 – 1.07) ^§^ |  |  |
|  |  |  |  | 1-7 d | 172/2721 | OR 0.51 (95% CI 0.41 – 0.64) ^§^ |  |  |
|  |  |  |  | >7 d | 61/1298 | OR 0.37 (95% CI 0.27 – 0.51) ^§^ |  |  |
| Nair 2009 (24) | Singleton neonates without congenital anomalies admitted to NICU | 24 - 28 | 163 | No ACS | 10/27 | REF | No difference in odds of IVH at <24 h compared to no ACS | None |
|  |  |  |  | <24 h | 12/29 | OR 1.2 (95% CI 0.41 – 3.52) |  |  |
| Peaceman 2005 (28) | Singleton and multiple neonates | 26 - 34 | 197 | ≤7 d | 2/99 | Too few events | - | None |
|  |  |  |  | >7 d | 0/98 | Too few events |  |  |
| Ryu 2019 (29) | Singleton neonates born to women with and without histological chorioamnionitis | 23 - 34 | 254 | No ACS | 2/35 | Too few events | No difference in odds of IVH at any time interval compared to no ACS | None |
|  |  |  |  | 2-7 d | 5/91 | Too few events in reference group |  |  |
|  |  |  |  | <48 h or >7 d | 5/128 | Too few events in reference group |  |  |
| Schmidt 2011 (30) | Singleton and multiple neonates with birthweight 500-999 g | NR | 1995 | No ACS | 27/104 | REF | All time intervals were associated with decreased odds of IVH compared to no ACS | <24 h, ≥24 h - <7 d, ≥7 d |
|  |  |  |  | <24 h | 20/139 | OR 0.48 (95% CI 0.25 – 0.91) ^§^ |  |  |
|  |  |  |  | ≥24 h - <7 d | 23/248 | OR 0.29 (95% CI 0.16 – 0.54) ^§^ |  |  |
|  |  |  |  | ≥7 d | 5/74 | OR 0.21 (95% CI 0.08 - 0.57) ^§^ |  |  |
| Sehdev 2004 (31) | Singleton neonates with birth weight 500-1500g born to women admitted for preterm labour, premature rupture of membranes, or indicated for labour (chorioamnionitis, non-reassuring fetal testing) | <28 | 325 | <24 h | 2/44 | Too few events | - | None |
|  |  |  |  | 24 h – 48 h | 2/95 | Too few events |  |  |
|  |  |  |  | 2 - <7 d | 2/106 | Too few events |  |  |
|  |  |  |  | ≥7 d | 3/80 | Too few events |  |  |
| Sen 2002 (32) | Singleton and multiple neonates admitted to NICU having received surfactant within first 2hrs of life | <31 | 226 | No ACS | 26/89 | REF | Interval 24 h – 7 d was associated with decreased odds of IVH compared to no ACS | 24 h – 7 d |
|  |  |  |  | 4-24 h | 4/69 | Too few events |  |  |
|  |  |  |  | 24 h - 7 d | 5/68 | OR 0.19 (95% CI 0.07 – 0.53) ^§^ |  |  |
| Siegler 2022 (33) | Singleton neonates | 24 – 34 | 327 | <2 d | 4/200 (2.0%) | Too few events | - | None |
|  |  |  |  | 2-7 d | 7/172 (4.1%) | Too few events in reference group |  |  |
| Tomotaki 2021 (51) | VLBW neonates | <30 | 115 | No ACS or <24 h | 5/36 (13.9%) | REF | - | None |
|  |  |  |  | 1-7 d | 2/41 (4.9%) | Too few events |  |  |
|  |  |  |  | >8 d | 2/38 (5.3%) | Too few events |  |  |
| Vermillion 2001 (52) | Women with singleton pregnancy, intact membranes and no fetal anomalies | 28 - 34 | 216 | 1-2 d | 1/97 | Too few events | - | None |
|  |  |  |  | 3-7 d | 1/78 | Too few events |  |  |
|  |  |  |  | 8-14 d | 0/41 | Too few events |  |  |
| Wong 2014 (34) | Singleton or multiple neonates without congenital anomalies admitted to NICU | <29 | 2549 | No ACS | 53/284 | REF | Intervals of <2 d - 7 d  and >7 d were associated with decreased odds of IVH compared to no ACS. | <2 d - 7 d; >7 d |
|  |  |  |  | <24 h | 131/644 | OR 1.11 (95% CI 0.78 – 1.59) |  |  |
|  |  |  |  | <2 d - 7 d | 99/1230 | OR 0.38 (95% CI 0.27 – 0.55) |  |  |
|  |  |  |  | >7 d | 120/1486 | OR 0.36 (95% CI 0.28 – 0.45) |  |  |

§ Crude odds ratios and 95% confidence intervals calculated by review authors using data provided in published paper. NR: Not reported. The brief narrative summary is based on available effect estimates, and the author’s conclusions for that study

**Supplementary Table S10. Summary of findings on antenatal corticosteroid administration-to-birth interval and Necrotizing enterocolitis (NEC) (Randomized Controlled Trials)**

| **Study** | **Population** | **GA (weeks)** | **N** | **Time intervals** | **NEC (n/N) %** | **Effect estimate** | **Narrative Summary** | **Optimal ACS administration-to-birth interval** |
| --- | --- | --- | --- | --- | --- | --- | --- | --- |
| Dexiprom  1999 (1) | Singleton and multiple neonates of women with preterm premature rupture of membranes | 28 - 24 | 208 | No ACs | 8/103 (7.8%) | REF | **-** | None |
|  |  |  |  | <24 h | 2/30 (6.7%) | Too few events |  |  |
|  |  |  |  | >24 h | 4/75 (5.3%) | Too few events |  |  |

**Supplementary Table S11. Summary of findings on antenatal corticosteroid administration-to-birth interval and necrotizing enterocolitis (NEC) (Observational studies)**

| **Study** | **Population** | **GA (weeks)** | **N** | **Time intervals** | **NEC (n/N) %** | **Effect estimate** | **Narrative Summary^1^** | **Optimal ACS administration-to-birth interval** |
| --- | --- | --- | --- | --- | --- | --- | --- | --- |
| Battarbee 2020 (10) | Singleton neonates | 23 - 34 | 2259 | <2 d | 31/622 (4.9%) | REF | Intervals of 2-7 d and 7-14 d were associated with increased odds of NEC compared to no <2 d. | <2 d |
|  |  |  |  | 2-7 d | 80/821 (9.7 %) | OR 2.06 (95%CI 1.34 – 3.16)^§^ |  |  |
|  |  |  |  | 7-14 d | 45/401 (11.2%) | OR 2.41 (95%CI 1.50 – 3.88)^§^ |  |  |
|  |  |  |  | ≥14 d | 33/415 (7.9%) | OR 1.65 (95%CI 0.99 – 2.73)^§^ |  |  |
| Biedermann 2022 (11) | Neonates with a birthweight <1500g admitted to NICU | <34 | 239 | No ACS | 0/7 (0.0%) | Too few events | - | None |
|  |  |  |  | <48 h | 6/47 (12.8%) | Too few events |  |  |
|  |  |  |  | 2-7 d | 5/90 (5.6%) | Too few events |  |  |
|  |  |  |  | >7 d | 1/95 (1.1%) | Too few events |  |  |
| Chawla 2010 (12) | Singleton neonates with birthweight 401-1000g and without congenital anomalies | ≤28 | 169 | No ACS | 3/23 (13.0%) | REF | Interval of >7 d was associated with increased odds of NEC compared to no ACS. | None |
|  |  |  |  | <24 h | 13/46 (28.3%) | OR 2.63 (95% CI 0.67- 10.36)^§^ |  |  |
|  |  |  |  | 24 h – 7 d | 16/52 (30.7%) | OR 2.96 (95% CI 0.77 – 11.42)^§^ |  |  |
|  |  |  |  | >7 d | 15/38 (39.5%) | OR 4.35 (95% CI 1.1 – 17.23)^§^ |  |  |
| Fuller 2017 (14) | Singleton neonates | 23 - 34 | 548 | No ACS | 2/58 (3.5%) | REF | No difference in odds of NEC at any time interval compared to no ACS | **None** |
|  |  |  |  | 1-23 h | 2/116 (1.7%) | OR 0.49 (95% CI 0.07 – 3.58) |  |  |
|  |  |  |  | 24-47 h | 2/26 (7.7%) | OR 2.33 (95% CI 0.31 – 17.55) |  |  |
|  |  |  |  | 2-7 d | 11/190 (5.8%) | OR 1.72 (95% CI 0.37 – 8.00) |  |  |
|  |  |  |  | ≥7 d | 10/158 (6.3%) | OR 1.89 (95% CI 0.4 – 8.91) |  |  |
| Haas 2006 (16) | Singleton neonates without congenital anomalies or fetal demise | 24-36 | 163 | <24 h | 3/113 (2.7%) | REF | **-** | None |
|  |  |  |  | 1-2 d | 0/50 (0%) | Too few events |  |  |
| Karmoker 2020 (17) | Singleton neonates without congenital anomalies pregnancies | 24 - 34 | 200 | 2 d – <7 d | 0/140 (0%) | REF | **-** | None |
|  |  |  |  | 7-14 d | 0/60 (0%) | Too few events |  |  |
| Kosinska- Kaczynska 2016 (3) | Women with twin pregnancy | 26-34 | 211 | <7 d | 3/99 (3.0%) | REF | **-** | None |
|  |  |  |  | ≥7 d | 0/112 (0) | Too few events |  |  |
| Kuk 2013 (18) | Twin neonates | 23 – 34 | 468 | No ACS | 4/122 (3.3%) | REF | No difference in odds of NEC at <2 d compared to no ACS | None |
|  |  |  |  | <2 d | 5/166 (3.0%) | OR 0.92 (95% CI 0.24 – 3.49)^§^ |  |  |
|  |  |  |  | 2-7 d | 3/114(2.6%) | Too few events |  |  |
|  |  |  |  | >7 d | 1/66 (1.5%) | Too few events |  |  |
| Liebowitz 2016 (20) | Singleton and multiple neonates without major anomalies admitted to the NICU. | <28 | 667 | <6 h | 28/136 (20.6%) | REF | No difference in odds of NEC at any time interval compared to <6 h | None |
|  |  |  |  | 7-23 h | 10/48 (20.8%) | OR 1.02 (95%CI 0.45 – 2.28)^§^ |  |  |
|  |  |  |  | ≥24 h | 53/380 (13.9%) | OR 0.63 (95%CI 0.38 – 1.04)^§^ |  |  |
| Melamed 2015 (23) | Singleton live-born neonates admitted to level III NICU | 24-34 | 6870 | No ACS | 48/1378 (3.5%) | aOR 0.97 (95% CI 0.65-1.45) | No difference in odds of NEC at any time interval compared to 1-7 days. | None |
|  |  |  |  | <24 h | 60/1473 (4.0%) | aOR 0.99 ((5% CI 0.70-1.40) |  |  |
|  |  |  |  | 1-7 d | 108/2721 (3.9%) | REF |  |  |
|  |  |  |  | >7 d | 57/1298 (4.4%) | aOR 1.32 (0.94-1.85) |  |  |
| Peaceman 2005 (28) | Single and multiple neonates | 26-34 | 197 | ≤7 d | 6/99 (6.1%) | REF | - | None |
|  |  |  |  | >7 d | 4/98 (4.1%) | Too few events |  |  |
| Ryu 2019 ^(29)^ * | Singleton neonates born to women with and without histological chorioamnionitis | 23 - 34 | 254 | No ACS | 1/35 (2.9%) | REF | - | None |
|  |  |  |  | 2-7 d | 3/91 (3.3%) | Too few events |  |  |
|  |  |  |  | <48 h or >7 d | 2/128 (16.0%) | Too few events |  |  |
| Sehdev 2004 (31) | Singleton neonates with birth weight 500-1500g born to women admitted for preterm labour, premature rupture of membranes, or indicated for labour (chorioamnionitis, non-reassuring fetal testing) | <28 | 325 | <24 h | 1/44 (2.3%) | REF | - | None |
|  |  |  |  | 24 h-48 h | 1/95 (1.1%) | Too few events |  |  |
|  |  |  |  | 48 h-7 d | 3/106 (2.8%) | Too few events |  |  |
|  |  |  |  | >7 d | 1/80 (1.3%) | Too few events |  |  |
| Sen 2002 (32) | Singleton and multiple neonates admitted to NICU having received surfactant within first 2hrs of life | <31 | 226 | No ACS | 7/89 (7.9%) | REF | No difference in odds of NEC at 4-24 h compared to no ACS | None |
|  |  |  |  | 4-24 h | 5/69 (7.3%) | OR 0.92 (95% CI 0.28 – 3.02)^§^ |  |  |
|  |  |  |  | 24 h-7 d | 4/68 (5.9%) | Too few events |  |  |
| Siegler 2022 (33) | Singleton neonates | 24 – 34 | 327 | <2 d | 2/200 (1.0%) | Too few events | **-** | None |
|  |  |  |  | 2-7 d | 1/172 (0.6%) | Too few events |  |  |

*Ryu 2019 used definition of NEC as stage >/=2b. § Crude odds ratios and 95% confidence intervals calculated by review authors using data provided in published paper. Where OR was reported by original study authors in cases of fewer than 5 events, we have included these ORs. NR: Not reported. The brief narrative summary is based on available effect estimates, and the author’s conclusions for that study

**Supplementary Table S12. Summary of findings on antenatal corticosteroid administration-to-birth interval and Bronchopulmonary Dysplasia (BPD) (Observational studies)**

| **Study** | **Population** | **GA (weeks)** | **N** | **Time intervals** | **BPD (n/N) %** | **Effect estimate** | **Narrative Summary^1^** | **Optimal ACS administration-to-birth interval** |
| --- | --- | --- | --- | --- | --- | --- | --- | --- |
| Battarbee 2020 (10) | Singleton neonates | 23 - 34 | 2259 | <2 d | 67/622 (10.8%) | REF | Intervals of 2-7 d and 7-14 d were associated with increased odds of BPD compared to <2 d. | <2 d |
|  |  |  |  | 2-7 d | 146/821 (17.9 %) | OR 1.79 (95%CI 1.31 – 2.44)^§^ |  |  |
|  |  |  |  | 7-14 d | 67/401 (16.8%) | OR 1.66 (95%CI 1.15 – 2.39) ^§^ |  |  |
|  |  |  |  | >14 d | 41/415 (10.0%) | OR 0.91 (95%CI 0.60 – 1.37) ^§^ |  |  |
| Biedermann 2022 (11) | Neonates with a birthweight <1500g admitted to NICU | <34 | 239 | No ACS | 1/7 (14.3%) | Too few events | - | None |
|  |  |  |  | <48 h | 4/47 (8.5%) | Too few events |  |  |
|  |  |  |  | 2-7 d | 15/90 (5.6%) | Too few events in reference group |  |  |
|  |  |  |  | >7 d | 7/95 (7.4%) | Too few events in reference group |  |  |
| Chawla 2010 (12) | Singleton neonates with birthweight 401-1000g and without congenital anomalies | ≤28 | 169 | No ACS | 9/12 (75%) | REF | No difference in odds of BPD at any time interval compared to no ACS. | None |
|  |  |  |  | <24 h | 14/21 (67%) | OR 0.82 (95%CI 0.30 – 2.27) ^§^ |  |  |
|  |  |  |  | 24 h – 7 d | 20/37 (54%) | OR 1.21 (95%CI 0.46 – 3.21) ^§^ |  |  |
|  |  |  |  | >7 d | 17/35 (49%) | OR 1.42 (95%CI 0.51 – 3.90) ^§^ |  |  |
| Haas 2006 (16) | Singleton neonates without congenital anomalies or fetal demise | 24 - 36 | 163 | <24 h | 15/113 (13%) | REF | No difference in odds of BPD at 24-48 h compared to <24 h. | None |
|  |  |  |  | 24-48 h | 6/50 (12%) | OR 0.89 (95%CI 0.32 – 2.45) ^§^ |  |  |
| Fortmann 2022 (13) | VLBW infants | 23 – 30 | 672 | No ACS | 52/238 (21.8%) | REF | No difference in odds of BPD at any time interval compared to no ACS. | None |
|  |  |  |  | 0 – 24 h | 16/70 (22.9%) | OR 1.06 (95% CI 0.56 – 2.00) ^§^ |  |  |
|  |  |  |  | 24h – 7d | 38/187 (20.3%) | OR 0.91 (95% CI 0.57 – 1.46) ^§^ |  |  |
|  |  |  |  | >7 d | 30/177 (16.9%) | OR 0.73 (95% CI 0.44 – 1.20) ^§^ |  |  |
| Kosinska- Kaczynska 2016 (3) | Women with twin pregnancy | 26 - 34 | 211 | <7 d | 16/99 (16.0%) | REF | No difference in odds of BPD at <7 d compared to ≥7 d. | None |
|  |  |  |  | ≥7 d | 21/112 (19.0%) | OR 1.20 (95%CI 0.59 – 2.45) ^§^ |  |  |
| Li 2022 (21) | Neonates admitted to NICU | 24-30 | 706 | <24 h | 29/228 (12.7%) | aOR 1.3 (95% CI 0.7 – 2.6) | No difference in the odds of BPD at any time interval compared to 2-7 d. | None |
|  |  |  |  | 1-2 d | 7/76 (9.2%) | aOR 1.4 (0.5 – 3.7) |  |  |
|  |  |  |  | 2-7 d | 30/275 (10.9%) | REF |  |  |
|  |  |  |  | >7 d | 2/67 (3.1%) | aOR 0.4 (95% CI 0.1 – 1.8) |  |  |
| Liebowitz 2016 (20) | Singleton and multiple neonates without major anomalies admitted to the NICU. | <28 | 667 | <6 h | 40/123 (32.5%) | REF | No difference in odds of BPD at any time interval compared to <6 h. | None |
|  |  |  |  | 7-24 h | 12/42 (28.6%) | OR 0.83 (95%CI 0.38 – 1.79) ^§^ |  |  |
|  |  |  |  | ≥24 h | 115/363 (31.7%) | OR 0.96 (95%CI 0.62 – 1.49) ^§^ |  |  |
| Melamed 2015 (23) | Singleton live-born neonates admitted to level III NICU | 24 - 34 | 6870 | No ACS | 138/1378 (11.0%) | aOR 1.45 (95%CI 1.10 – 1.91) | Intervals of <24 h, >7 d and no ACS were associated with increased odds of BPD compared to 1-7 d. | 1-7 d |
|  |  |  |  | <24 h | 385/1473 (15.0%) | aOR 1.26 (95%CI 1.00 – 1.59) |  |  |
|  |  |  |  | 1-7 d | 173/2721 (14.0%) | REF |  |  |
|  |  |  |  | >7 d | 187/1298 (14.0%) | aOR 1.39 (95%CI 1.11 – 1.75) |  |  |
| Nair 2009 (24) | Singleton neonates without congenital anomalies admitted to NICU | 24 - 28 | 163 | No ACS | 11/27 (41.0%) | REF | No difference in odds of BPD at <24 h compared to no ACS. | None |
|  |  |  |  | <24 h | 14/29 (50.0%) | OR 1.36 (95%CI 0.47 – 3.91) |  |  |
| Palas 2018 (27) | Twin neonates admitted to NICU | 24 - 31 | 750 | No ACS | 11/19 (57.9%) | REF | Intervals of ≤7 d and >7 d were associated decreased odds of BPD compared to no ACS. | ≤7 d; >7 d |
|  |  |  |  | ≤7 d | 25/272 (9.2%) | OR 0.07 (95%CI 0.03 – 0.20) ^§^ |  |  |
|  |  |  |  | >7 d | 20/230 (8.7%) | OR 0.07 (95%CI 0.02 – 0.19) ^§^ |  |  |
| Ryu 2019 (29) | Singleton neonates born to women with and without histological chorioamnionitis | 23 - 34 | 254 | No ACS | 11/35 (31.4%) | REF | No difference in odds of BPD at any time interval compared to no ACS. | None |
|  |  |  |  | 2-7 d | 17/91 (18.7%) | OR 0.50 (95%CI 0.21 – 1.22) ^§^ |  |  |
|  |  |  |  | <48 h or >7 d | 26/128 (20.3%) | OR 0.56 (95%CI 0.24 – 1.28) ^§^ |  |  |
| Sehdev 2004 (31) | Singleton neonates with birthweight 500-1500g born to women admitted for preterm labour, premature rupture of membranes, or indicated for labour (chorioamnionitis, non-reassuring fetal testing) | <28 | 325 | <24 h | 14/44 (32.0%) | OR 0.63 (95%CI 0.26 – 1.48) | No difference in odds of BPD at any time interval compared to 2-7 d. | None |
|  |  |  |  | 24-48 h | 27/95 (28.0%) | OR 0.60 (95%CI 0.31 – 1.16) |  |  |
|  |  |  |  | 2-7 d | 44/106 (42.0%) | REF |  |  |
|  |  |  |  | >7 d | 33/80 (36.0%) | OR 1.18 (95%CI 0.60 – 2.29) |  |  |
| Siegler 2022 (33) | Singleton neonates | 24 – 34 | 327 | <2 d | 1/200 (0.5%) | REF | - | None |
|  |  |  |  | 2-7 d | 1/172 (0.6%) | Too few events |  |  |
| Tomotaki 2021 (51) | VLBW neonates | <30 | 115 | No ACS or <24 h | 24/36 (66.7%) | REF | No difference in odds of BPD at any time interval compared to No ACS or <24 h. | None |
|  |  |  |  | 1-7 d | 31/41 (75.6%) | OR 1.55 (95% CI 0.57 – 4.19) ^§^ |  |  |
|  |  |  |  | >8 d | 28/38 (73.7%) | OR 1.40 (95% CI 0.51 – 3.81) ^§^ |  |  |
| Wilms 2011 (54) | Singleton or multiple neonates | 24^5^ - 34 | 254 | <7 d | 22/146 (15.0%) | REF | Interval of 15-21 d was associated with increased odds of BPD compared to <7 d. | None |
|  |  |  |  | 8-14 d | 6/53 (11.0%) | OR 1.4 (95%CI 0.46 – 41.0) |  |  |
|  |  |  |  | 15-21 d | 5/24 (32.0%) | OR 4.0 (95%CI 1.10 – 15.0) |  |  |
| Wong 2014 (34) | Singleton or multiple neonates without congenital anomalies admitted to NICU | <29 | 2549 | No ACS | 84/319 (26.3%) | REF | Interval of 2-7 d was associated with increased odds of BPD compared to no ACS. | None |
|  |  |  |  | <24 h | 168/677 (24.8%) | OR 0.92 (95%CI 0.68 – 1.25) |  |  |
|  |  |  |  | 2-7 d | 425/1281 (33.2%) | OR 1.39 (95%CI 1.06 – 1.83) |  |  |
|  |  |  |  | >7 d compared to no ACS + <24 h | 509/1553 (32.8%) | OR 1.44 (95%CI 1.21 –1.72) |  |  |

§ Crude odds ratios and 95% confidence intervals calculated by review authors using data provided in published paper. NR: Not reported. The brief narrative summary is based on available effect estimates, and the author’s conclusions for that study

**Supplementary Table S13. Summary of findings on antenatal corticosteroid administration-to-birth interval and neonatal sepsis (observational studies)**

| **Study** | **Population** | **GA (weeks)** | **N** | **Time intervals** | **NN Sepsis (n/N) %** | **Effect estimate** | **Narrative Summary^1^** | **Optimal ACS administration-to-birth interval** |
| --- | --- | --- | --- | --- | --- | --- | --- | --- |
| Barrett 1982 (9) | Women with singleton or multiple pregnancy with ruptured membranes and no chorioamnionitis, fetal distress or abrupt placentae | 26 - 34 | 93 | No ACS | 1/56 (1.78%) | REF | - | None |
|  |  |  |  | ≤24 h | 0/2 (0%) | Too few events |  |  |
|  |  |  |  | 24 - 47 h | 1/6 (16.67%) | Too few events |  |  |
|  |  |  |  | 48 - 71 h | 0/13 (0%) | Too few events |  |  |
|  |  |  |  | 72 - 95 h | 0/4 (0%) | Too few events |  |  |
|  |  |  |  | 96 - 143 h | 0/2 (0%) | Too few events |  |  |
|  |  |  |  | >144 h | 0/9 (0%) | Too few events |  |  |
| Biedermann 2022 (11) | Neonates with a birthweight <1500g admitted to NICU | <34 | 239 | No ACS | 0/7 (0%) | Too few events | - | None |
|  |  |  |  | <48 h | 3/47 (6.4%) | Too few events |  |  |
|  |  |  |  | 2-7 d | 14/90 (15.6%) | Too few events in reference group |  |  |
|  |  |  |  | >7 d | 4/95 (4.2%) | Too few events |  |  |
| Chawla 2010 (12) | Singleton neonates with birthweight 401-1000g and without congenital anomalies | ≤28 | 169 | No ACS | 7/16 (43.75%) | REF | No difference in odds of sepsis at any time interval compared to no ACS. | None |
|  |  |  |  | <24 h | 20/40 (50%) | OR 1.29 (95% CI 0.4- 4.13) ^§^ |  |  |
|  |  |  |  | 24 h – 7 d | 20/41 (48.78%) | OR 1.22 (95% CI 0.38-3.91) ^§^ |  |  |
|  |  |  |  | >7 d | 22/35 (62.86%) | OR 2.18 (95% CI 0.65 - 7.24) ^§^ |  |  |
| Ferguson 2009 (44) | Women with singleton pregnancy and severe hypertension of pregnancy | 26 - 34 | 172 | ≤48 h | 13/55 (23.63%) | REF | No difference in odds of sepsis at >48 h compared to ≤48 h. | None |
|  |  |  |  | >48 h | 21/117 (17.95%) | OR 0.71 (95% CI 0.32-1.54) |  |  |
| Fortmann 2022 (13) | VLBW infants | 23 – 30 | 672 | No ACS | 40/238 (16.8%) | REF | No difference in odds of sepsis at any time interval compared to no ACS. | None |
|  |  |  |  | 0 – 24 h | 11/70 (15.7%) | OR 0.92 (95% CI 0.45 – 1.91) ^§^ |  |  |
|  |  |  |  | 24h – 7d | 27/187 (14.4%) | OR 0.84 (95% CI 0.49 – 1.42) ^§^ |  |  |
|  |  |  |  | >7 d | 30/177 (16.9%) | OR 1.01 (95% CI 0.60 – 1.70) ^§^ |  |  |
| Fuller 2017 (14) | Singleton neonates. | 23 - 34 | 548 | No ACS | 4/58 (6.9%) | REF | No difference in odds of sepsis at any time interval compared to no ACS. | None |
|  |  |  |  | 1-23 h | 6/116 (5.2%) | OR 0.98 (95% CI 0.09 – 11.08) |  |  |
|  |  |  |  | 24-47 h | 0/26 (0%) | Too few events |  |  |
|  |  |  |  | 2-7 d | 8/190 (4.2%) | OR 1.98 (95% CI 0.23 – 16.96) |  |  |
|  |  |  |  | >7 d | 5/158 (3.2%) | OR 0.34 (95% CI 0.02 – 5.58) |  |  |
| Kosinska- Kaczynska 2016 (3) | Women with twin Pregnancy | 26 - 37 | 211 | <7 d | 2/99 (2.02) | REF | - | None |
|  |  |  |  | ≥ 7 d | 0/112 (0%) | Too few events |  |  |
| Kuk 2013 (18) | Twin neonates | 23 - 34 | 468 | No ACS | 37/122 (30.33%) | REF | No difference in odds of sepsis at any time interval compared to no ACS. | None |
|  |  |  |  | < 2 d | 37/166 (22.29%) | OR 0.66 (95% CI 0.39 - 1.12) ^§^ |  |  |
|  |  |  |  | 2-7 d | 33/114 (28.95%) | OR 0.94 (95% CI 0.53 - 1.64) ^§^ |  |  |
|  |  |  |  | >7 d | 13/66 (19.7%) | OR 0.56 (95% CI 0.27-1.16) ^§^ |  |  |
| Liebowitz 2016 (20) | Singleton and multiple neonates without major anomalies admitted to level III NICU | <28 | 667 | ≤6 h | 7/183 (3.8%) | REF | No difference in odds of sepsis at any time interval compared to ≤6 h. | None |
|  |  |  |  | ≥24 h | 21/429 (4.9%) | OR 1.29 (95% CI 0.54-3.1) ^§^ |  |  |
|  |  |  |  | <10 d | 12/312 (3.84%) | OR 1.01 (95% CI 0.39-2.6) ^§^ |  |  |
|  |  |  |  | ≥10 d | 6/80 (7.5%) | OR 2.04 (95% CI 0.66-6.27) ^§^ |  |  |
| Peaceman 2005 (28) | Single and multiple neonates | 26 - 34 | 197 | ≤7 d | 19/99 (19.19%) | REF | No difference in odds of sepsis at ≥7 d compared to ≤7 d. | None |
|  |  |  |  | ≥7 d | 22/98 (022.45%) | OR 1.22 (95% CI 0.61-2.43) ^§^ |  |  |
| Ryu 2019 (29) | Singleton neonates born to women with and without histological chorioamnionitis | 23 - 37 | 254 | No ACS | 6/35 (17.14%) | REF | No difference in odds of sepsis at any time interval compared to no ACS. | None |
|  |  |  |  | 2-7 d | 16/91 (17.58%) | OR 1.03 (95% CI 0.37-2.89) ^§^ |  |  |
|  |  |  |  | <48 h or >7 d | 18/128 (14%) | OR 0.79 (95% CI 0.29-2.17) ^§^ |  |  |
| Vermillion 2001 (52) | Women with singleton pregnancy, intact membranes and no fetal anomalies | 28 - 34 | 216 | 1-2 d | 1/97 (1%) | REF | - | None |
|  |  |  |  | 3-7 d | 0/78 (0%) | Too few events |  |  |
|  |  |  |  | 8-14 d | 1/41 (2.44%) | Too few events |  |  |
| Wong 2014 (34) | Singleton or multiple neonates without congenital anomalies admitted to NICU | <29 | 2549 | No ACS | 100/319 (31.4%) | REF | Intervals of 2-7 d and >7 d were associated with increased odds of sepsis compared to no ACS. | None |
|  |  |  |  | <24 h | 218/677 (32.2%) | OR 1.04 (0.78 - 1.39) |  |  |
|  |  |  |  | 2-7 d | 498/1281 (38.9%) | OR 1.39 (1.07 - 1.81) |  |  |
|  |  |  |  | >7 d | 594/1553 (38.3%) | OR 1.32 (95% CI 1.12 – 1.56) |  |  |

§ Crude odds ratios and 95% confidence intervals calculated by review authors using data provided in published paper. NR: Not reported. The brief narrative summary is based on available effect estimates, and the author’s conclusions for that study

**Supplementary Table S14. Summary of findings on antenatal corticosteroid administration-to-birth interval and NICU admission (Observational studies)**

| **Study** | **Population** | **GA (weeks)** | **N** | **Time intervals** | **NICU Admit (n/N) %** | **Effect estimate** | **Narrative Summary** | **Optimal ACS administration-to-birth interval** |
| --- | --- | --- | --- | --- | --- | --- | --- | --- |
| Kosinska- Kaczynska 2016 (3) | Women with twin pregnancy | 26^0^-33^6^ | 211 | <7 d | 65/99 (65.7) | REF | No difference in odds of NICU admission at ≥7 d compared to <7 d. | None |
|  |  |  |  | ≥7 d | 84/112 (75) | OR 1.57 (95%CI 0.86 – 2.85) ^§^ |  |  |
| Kuk 2013 (18) | Twin neonates | 23 – 34 | 468 | No ACS | 122/122 (100) | REF | **-** | None |
|  |  |  |  | <2 d | 166/166 (100) | OR NaN (95% CI NaN – NaN) ^§^ |  |  |
|  |  |  |  | 2 – 7 d | 110/114 (96.5) | OR 0 (95% CI 0 – NaN ) ^§^ |  |  |
|  |  |  |  | >7 d | 65/66 (98.5) | OR 0 (95% CI 0 -NaN) ^§^ |  |  |

§ Crude odds ratios and 95% confidence intervals calculated by review authors using data provided in published paper. NR: Not reported. The brief narrative summary is based on available effect estimates, and the author’s conclusions for that study

**Supplementary Table S15. Summary of findings on antenatal corticosteroid administration-to-birth interval and neonatal hypoglycaemia (Observational Studies)**

| **Study** | **Population** | **GA (weeks)** | **N** | **Time intervals** | **Hypoglycaemia (n/N) %** | **Effect estimate** | **Narrative Summary^1^** | **Optimal ACS administration-to-birth interval** |
| --- | --- | --- | --- | --- | --- | --- | --- | --- |
| Gulersen  2021 (46) | Singleton neonates. | 34 - 37 | 1248 | <2 d | 383/772 (49.6) | aOR 3.44 (95% CI 2.10 – 5.63) | Interval of <2 d was associated with increased odds of hypoglycaemia compared to 2-7 d; interval of >7 d was associated with decreased hypoglycaemia compared to 2-7d | >7 d |
|  |  |  |  | 2-7 d | 61/168 (36.6) | REF |  |  |
|  |  |  |  | >7 d | 47/308 (15.3) | aOR 0.32 (95% CI 0.20 – 0.51) |  |  |
| di Pasquo  2020 (56) | Early preterm (born 24^0^ – 33^6^ and late preterm (born 34^0^ – 36^6^ neonates | 24 – 34 and 34 – 37 | 99 | 24 h - 7 d | 13/34 (38.2) | REF | No difference in odds of hypoglycaemia for <24h and >7 d compared to 24 h – 7d | None |
|  |  |  |  | <24 h and >7 d | 25/65 (38.5) | OR 1.01 (95% CI 0.43 – 2.37) |  |  |

The brief narrative summary is based on available effect estimates, and the author’s conclusions for that study

**Supplementary Table S16. Summary of findings on antenatal corticosteroid administration-to-birth interval and retinopathy of prematurity* (observational studies)**

| **Study** | **Population** | **GA (weeks)** | **N** | **Time intervals** | **Retinopathy of prematurity (n/N) %** | **Effect estimate** | **Narrative Summary^1^** | **Optimal ACS administration-to-birth interval** |
| --- | --- | --- | --- | --- | --- | --- | --- | --- |
| Chawla 2010 (12) | Singleton neonates with birthweight 401-1000g and without congenital anomalies | ≤28 | 169 | No ACS | 1/27 (3.7%) | REF | No difference in odds of retinopathy of prematurity at any time interval compared to no ACS. | None |
|  |  |  |  | <24 h | 3/48 (6.3%) | Too few events |  |  |
|  |  |  |  | 24 h - 7 d | 5/53 (9.4%) | OR 2.71 (95% CI 0.30-24.43) ^§^ |  |  |
|  |  |  |  | >7 d | 3/41 (7.3%) | Too few events |  |  |
| Fuller 2017 (14) | Singleton neonates | 23 - 34 | 548 | No ACS | 6/58 (10.3%) | REF | No difference in odds of retinopathy of prematurity at any time interval compared to no ACS. | None |
|  |  |  |  | 1-23 h | 5/116 (4.3%) | OR 0.39 (95% CI 0.11 – 1.34) ^§^ |  |  |
|  |  |  |  | 24-47 h | 3/26 (11.5%) | Too few events |  |  |
|  |  |  |  | 2-7 d | 21/190 (11.1%) | OR 1.08 (95% CI 0.41 – 2.81) ^§^ |  |  |
|  |  |  |  | >7 d | 8/158 (5.1%) | OR 0.46 (95% CI 0.15-1.39) ^§^ |  |  |
| Kuk 2013 (18) | Twin neonates | 23 - 34 | 468 | No ACS | 14/122 (11.5%) | REF | No difference in odds of retinopathy of prematurity at any time interval compared to no ACS. | None |
|  |  |  |  | < 2 d | 17/166 (10.2%) | OR 0.88 (95% CI 0.42 – 1.86) ^§^ |  |  |
|  |  |  |  | 2-7 d | 10/114 (8.9%) | OR 0.74 (95% CI 0.32 – 1.74) ^§^ |  |  |
|  |  |  |  | >7 d | 1/66 (1.5%) | Too few events |  |  |
| Liebowitz 2016 (20) | Singleton and multiple neonates without major anomalies admitted to the NICU | <28 | 667 | ≤6 h | 16/183 (35.0%) | REF | No difference in odds of retinopathy of prematurity at any time interval compared to ≤6 h. | None |
|  |  |  |  | ≥24 h | 12/429 (17.0%) | aOR 1.30 (95%CI 0.51 – 3.12) |  |  |
| Melamed 2015 (23) | Singleton live born neonates admitted to level III NICU | 24 - 34 | 6870 | No ACS | 27/1378 (7.8%) | aOR 1.63 (95%CI 0.94 – 2.83) | No difference in odds of retinopathy of prematurity at any time interval compared to 1-7 d. | None |
|  |  |  |  | <24 h | 33/1473 (6.0%) | aOR 1.15 (95%CI 0.71 – 1.84) |  |  |
|  |  |  |  | 1-7 d | 87/2721 (4.0%) | REF |  |  |
|  |  |  |  | >7 d | 36/1298 (4.0%) | aOR 1.49 (95% CI 0.96 – 1.85) |  |  |
| Ryu 2019 (29) | Singleton preterm neonates born to women with and without histological chrioamnionitis | 23 - 34 | 254 | No ACS | 4/35 (11.4%) | Too few events | - | None |
|  |  |  |  | 2-7 d | 4/91 (2.2%) | Too few events |  |  |
|  |  |  |  | <48 h or >7 d | 8/128 (3.9%) | Too few events (REF group) |  |  |
| Tomotaki 2021 (51) | VLBW neonates | <30 | 115 | No ACS or <24 h | 16/36 (44.4%) | REF | No difference in odds of retinopathy of prematurity at any time interval compared to no ACS or <24 h. | None |
|  |  |  |  | 1-7 d | 11/41 (26.8%) | OR 0.46 (95% CI 0.17 – 1.19) ^§^ |  |  |
|  |  |  |  | >8 d | 15/38 (39.5%) | OR 0.81 (95% CI 0.32 – 2.06) ^§^ |  |  |

*Definition of retinopathy of prematurity varied across studies. § Crude odds ratios and 95% confidence intervals calculated by review authors using data provided in published paper. NR: Not reported. The brief narrative summary is based on available effect estimates, and the author’s conclusions for that study

**Supplementary Table S17. Summary of findings on antenatal corticosteroid administration-to-birth interval and patent ductus arteriosus* (observational studies)**

| **Study** | **Population** | **GA (weeks)** | **N** | **Time intervals** | **Neonatal mortality (n/N) %** | **Effect estimate** | **Narrative Summary^1^** | **Optimal ACS administration-to-birth interval** |
| --- | --- | --- | --- | --- | --- | --- | --- | --- |
| Barrett 1982 (9) | Women with singleton or multiple pregnancy with ruptured membranes and no chorioamnionitis, fetal distress or abrupt placentae | 26 - 34 | 93 | No ACS | Not reported | REF | - | None |
|  |  |  |  | ≤24 h | 0/2 (0%) | Too few events |  |  |
|  |  |  |  | 24-47 h | 2/6 (33.3%) | Too few events |  |  |
|  |  |  |  | 48-71 h | 2/13 (7.6%) | Too few events |  |  |
|  |  |  |  | 72-95 h | 0/4 (0%) | Too few events |  |  |
|  |  |  |  | 96-143 h | 0/2 (0%) | Too few events |  |  |
|  |  |  |  | >144 h | 0/9 (0%) | Too few events |  |  |
| Chawla 2010 (12) | Singleton neonates with birthweight 401-1000g and without congenital anomalies | ≤28 | 169 | No ACS | 11/27 (40.7%) | REF | No difference in odds of patent ductus arteriosus at any time interval compared to no ACS | None |
|  |  |  |  | <24 h | 25/48 (52.1%) | OR 1.58 (95% CI 0.61 – 4.10) ^§^ |  |  |
|  |  |  |  | 24 h - 7 d | 31/53 (58.5%) | OR 2.05 (95% CI 0.80 – 5.26) ^§^ |  |  |
|  |  |  |  | >7 d | 17/41 (41.5%) | OR 1.03 (95% CI 0.38 – 2.77) ^§^ |  |  |
| Kuk 2013 (18) | Twin neonates | 23 - 34 | 468 | No ACS | 56/122 (45.9%) | REF | Interval of >7 d was associated with decreased odds of patent ductus arteriosus compared to no ACS. | >7 days |
|  |  |  |  | < 2 d | 70/166 (42.2%) | OR 0.86 (95% CI 0.54 – 1.38) ^§^ |  |  |
|  |  |  |  | 2-7 d | 42/114 (36.8%) | OR 0.69 (95% CI 0.41 – 1.16) ^§^ |  |  |
|  |  |  |  | >7 d | 20/66 (30.3%) | OR 0.51 (95% CI 0.27 – 0.97) ^§^ |  |  |
| Nair 2009 (24) | Singleton neonates without congenital anomalies admitted to NICU | 24 - 28 | 163 | No ACS | 12/27 (44.4%) | REF | No difference in odds of patent ductus arteriosus at <24 h compared to no ACS. | None |
|  |  |  |  | <24 h | 14/29 (48.3%) | OR 1.17 (95% CI 0.41 – 3.34) ^§^ |  |  |
| Ryu 2019 (29) | Singleton preterm neonates born to women with and without histological chrioamnionitis | 23 - 34 | 254 | No ACS | 16/35 (11.4%) | REF | No difference in odds of patent ductus arteriosus at any time interval compared to no ACS | None |
|  |  |  |  | 2-7 d | 29/91 (2.2%) | OR 0.56 (95% CI 0.25 – 1.23) ^§^ |  |  |
|  |  |  |  | <48 h or >7 d | 45/128 (3.9%) | OR 0.64 (95% CI 0.30 – 1.37) ^§^ |  |  |
| Sehdev 2004 (31) | Singleton neonates with birth weight 500-1500g born to women admitted for preterm labour, PROM, or indicated for labour (chorioamnionitis, non-reassuring fetal testing) | <28 | 325 | <24 h | 18/44 (40.9%) | OR 0.73 (95% CI 0.33 – 1.61) | No difference in odds of patent ductus arteriosus at any time interval compared to 48 h - 7 d. | None |
|  |  |  |  | 24-48 h | 44/95 (46.3%) | OR 1.16 (95% CI 0.64 – 2.21) |  |  |
|  |  |  |  | 48 h - 7 d | 46/106 (43.4%) | REF |  |  |
|  |  |  |  | >7 d | 27/80 (33.8%) | OR 0.74 (95% CI 0.39 – 1.41) |  |  |
| Wong 2014 (34) | Singleton or multiple neonates without congenital anomalies admitted to NICU | <29 | 2549 | No ACS | 142/319 (44.5%) | REF | Interval of >7 days was associated with decreased odds of patent ductus arteriosus compared to <24 h or no ACS. | >7 days |
|  |  |  |  | <24 h | 325/677 (48.0%) | OR 1.15 (95% CI 0.88 – 1.50) |  |  |
|  |  |  |  | 48-7 d | 512/1281 (40.0%) | OR 0.83 (95% CI 0.65 – 1.06) |  |  |
|  |  |  |  | No ACS/<24 h | 467/996 (46.9%) | REF (for 48 h-7 d/>7 d comparison) |  |  |
|  |  |  |  | >7 d | 594/1553 (38.2%) | OR 0.70 (95% CI 0.60 – 0.82) |  |  |
| Tomotaki 2021 (51) | VLBW neonates | <30 | 115 | No ACS or <24 h | 10/36 (27.8%) | REF | No difference in odds of patent ductus arteriosus at any time interval compared to no ACS or <24 h | None |
|  |  |  |  | 1-7 d | 12/41 (29.3%) | OR 1.08 (95% CI 0.40 – 2.90) ^§^ |  |  |
|  |  |  |  | >8 d | 14/38 (36.8%) | OR 1.52 (95% CI 0.57 – 4.05) ^§^ |  |  |

*Definition of patent ductus arteriosis varied across studies § Crude odds ratios and 95% confidence intervals calculated by review authors using data provided in published paper. NR: Not reported. The brief narrative summary is based on available effect estimates, and the author’s conclusions for that study

**Supplementary Table S18. Summary of findings on antenatal corticosteroid administration-to-birth interval and birthweight (observational studies)**

| **Study** | **Population** | **GA (weeks)** | **N** | **Time intervals** | **Mean Birthweight (g)** | **Standard Deviation** | **P value** | **Narrative Summary^1^** | **Optimal ACS administration-to-birth interval** |
| --- | --- | --- | --- | --- | --- | --- | --- | --- | --- |
| Battarbee  2020 (10) | Singleton neonates | 23 - 34 | 2259 | <2 d | 1486 | 550 | p <0.001 | Significant differences in birthweight across groups | NR |
|  |  |  |  | 2-<7 d | 1296 | 475 |  |  |  |
|  |  |  |  | 7-<14 d | 1310 | 458 |  |  |  |
|  |  |  |  | ≤14 d | 1585 | 469 |  |  |  |
| Chawla 2010 (12) | Singleton neonates with birthweight 401-1000g and without congenital anomalies | ≤28 | 169 | No ACS | 704 | 114 | *p =* 0.009 | Birthweight significantly greater in >7 d compared to other time intervals | >7 d |
|  |  |  |  | <24 h | 711 | 128 |  |  |  |
|  |  |  |  | 24 h – 7 d | 702 | 146 |  |  |  |
|  |  |  |  | >7 d | 786 | 118 |  |  |  |
| Dzidek 2020 (57) | Women with singleton or multiple pregnancy and threatened preterm birth, premature rupture of membranes, medical indications for birth or cervical incompetence | 24 - 34 | 530 | ≤7 d | 1769 | 644.15 | p <0.001 | Birthweight significantly greater in >7 d compared to ≤7 d | >7 d |
|  |  |  |  | >7 d | 2680.4 | 752.38 |  |  |  |
| Ferguson  2009 (44) | Women with singleton pregnancy and severe hypertension of pregnancy | 26 - 34 | 172 | ≤48 h | 1345 | 431 | p = 0.400 | No significant differences in birthweight across groups | None |
|  |  |  |  | >48 h | 1408 | 464 |  |  |  |
| Fuller 2017 (14) | Singleton neonates | 23 - 34 | 548 | No ACS | 1599 | 569 | p = 0.009 | Significant difference in birthweight across groups | NR |
|  |  |  |  | 1-23 h | 1529 | 512 |  |  |  |
|  |  |  |  | 24-47 h | 1558 | 599 |  |  |  |
|  |  |  |  | 2-7 d | 1408 | 542 |  |  |  |
|  |  |  |  | >7 d | 1607 | 542 |  |  |  |
| Hurrell 2022 (4) | Women delivering before 35 weeks' gestation with confirmed preeclampsia who were enrolled in the PELICAN study or PARROT trial | <35 | 250 | No ACS | 1294 | 582.2 | NR | No significant differences in birthweight across groups | None |
|  |  |  |  | ≤7 d | 1412 | 469.6 |  |  |  |
|  |  |  |  | >7 d | 1467 | 576.7 |  |  |  |
| Kosinska- Kaczynska 2016 (3) | Women with twin pregnancy | 26 - 34 | 211 | <7 d | Twin 1: 1495 Twin 2: 1540 | Twin 1: 500 Twin 2: 428 | Twin 1: p=0.1  Twin 2: p=0.3 | No significant differences in birthweight between groups | None |
|  |  |  |  | ≥7 d | Twin 1: 1638  Twin 2: 1452 | Twin 1: 339 Twin 2: 385 |  |  |  |
| Kuk 2013 (18) | Twin neonates | 23 - 34 | 468 | No ACS | 1417.4 | *445.5* | p <0.001 | Birthweight in >7 ds significantly greater compared to no ACS group. | >7 d |
|  |  |  |  | <2 d | 1484.4 | *424.2* |  |  |  |
|  |  |  |  | 2-7 d | 1712.0 | *364.9* |  |  |  |
|  |  |  |  | >7 d | 1461.2 | *455.8* |  |  |  |
| Lau 2017 (49) | Singleton and multiple neonates | 23^5^ - 37 | 352 | <2 d | 2170 | 60 | p = 0.025 | Significant difference in birthweight across groups | NR |
|  |  |  |  | 2-7 d | 1870 | 86 |  |  |  |
|  |  |  |  | >7 d | 2110 | 77 |  |  |  |
| Liebowitz 2016 (20) | Singleton and multiple neonates without major anomalies admitted to the NICU | <28 | 667 | ≤6 h | 830 | 188 | p = 0.12 between none or ≤6 h and ≥24 h | No significant differences in birthweight between groups | None |
|  |  |  |  | ≥24 h | 803 | 197 |  |  |  |
|  |  |  |  | <10 d | 766 | 193 | p <0.001 between <10 d and ≥10 d. | Birthweight in ≥10 d significantly greater compared to <10 d. | >10 d |
|  |  |  |  | ≥10 d | 919 | 183 |  |  |  |
| McEvoy 2008 (5) | Singleton and multiple neonates with birthweight ≤2000g and without congenital anomalies | 25 - 32 | 56 | 1-7d | 1485 | 332 | NR | No significant differences in birthweight across groups | None |
|  |  |  |  | >7d | 1517 | 302 |  |  |  |
|  |  |  |  | 8-14 d | 1552 | 328 |  |  |  |
|  |  |  |  | >14 d | 1498 | 296 |  |  |  |
| Melamed 2015 (23) | Singleton live born neonates admitted to level III NICU | 24 - 34 | 6870 | No ACS | 1668 | 581 | p <0.001 | Significant difference in birthweight across groups | NR |
|  |  |  |  | <24 h | 1551 | 533 |  |  |  |
|  |  |  |  | 1-7 d | 1394 | 506 |  |  |  |
|  |  |  |  | >7 d | 1493 | 502 |  |  |  |
| Nair 2009 (24) | Singleton neonates without congenital anomalies admitted to NICU | 24 - 28 | 163 | No ACS | 906 | 244 | NR | NR | None |
|  |  |  |  | <24 h | 923 | 248 |  |  |  |
| Peaceman 2005 (28) | Single and multiple neonates | 26 - 34 | 197 | ≤7 d | 1443 | 481 | p = 0.23 | No significant differences in birthweight across groups | None |
|  |  |  |  | >7 d | 1524 | 460 |  |  |  |
| Sehdev 2004 (31) | Singleton neonates with birthweight 500-1500g born to women admitted for preterm labour, premature rupture of membranes, or indicated for labour (chorioamnionitis, non-reassuring fetal testing) | <28 | 325 | <24 h | 980 | 287 | p = 0.43 | No significant differences in birthweight across groups | None |
|  |  |  |  | 24 h - 48 h | 1042 | 281 |  |  |  |
|  |  |  |  | 48 h -7 d | 1016 | 288 |  |  |  |
|  |  |  |  | >7 d | 1071 | 280 |  |  |  |
| Sekhavat 2011 (50) | Singleton neonates | 28 - 34 | 104 | < 2d | 1451 | 385 | p = 0.30 | No significant differences in birthweight across groups | None |
|  |  |  |  | 2-7 d | 1424 | 349 |  |  |  |
|  |  |  |  | >7 d | 1460 | 361 |  |  |  |
| Sen 2002 (32) | Singleton and multiple neonates admitted to NICU having received surfactant within first 2hrs of life | <31 | 226 | No ACS | 1090 | 95% CI 1020 - 1170 | p >0.2 | No significant differences in birthweight across groups | None |
|  |  |  |  | 4-24 h | 1170 | 95% CI 1090-1260 |  |  |  |
|  |  |  |  | 24 h- 7 d | 1120 | 95% CI 1060-1190 |  |  |  |
| Siegler 2022 (33) | Singleton neonates | 24 – 34 | 327 | <2 d | 1700 | 500 | p<0.0001 | Birthweight in <2d significantly greater compared to <2-7d. | <2 d |
|  |  |  |  | 2-7 d | 1500 | 500 |  |  |  |
| Vermillion 2001 (52) | Women with singleton pregnancy, intact membranes and no fetal anomalies | 28 - 34 | 216 | 1-2 d | 1243 | 237 | P = 0.73 | No significant differences in birthweight across groups | None |
|  |  |  |  | 3-7 d | 1340 | 271 |  |  |  |
|  |  |  |  | 8-14 d | 1199 | 301 |  |  |  |
| Yasuhi 2017 (55) | Women with singleton pregnancy with no fetal anomalies | 24 - 33 | 397 | <7 d | 1257 | 432 | <7 d vs 7-14 d: p=0.740  <7 d vs >14 d: p=0.033  7-14 d vs >14 d: p=0.071 | Birthweight in >14 d significantly greater than <7 d. | >14 d |
|  |  |  |  | 7-14 d | 1214 | 498 |  |  |  |
|  |  |  |  | >14 d | 1497 | 466 |  |  |  |

NR: Not reported. The brief narrative summary is based on available effect estimates, and the author’s conclusions for that study

**Supplementary Table S19. Summary of findings on antenatal corticosteroid administration-to-birth interval and chorioamnionitis (randomized controlled trials)**

| **Study** | **Population** | **GA (weeks)** | **N** | **Time intervals** | **Chorioamnionitis (n/N) %** | **Effect estimate** | **Narrative Summary** | **Optimal ACS administration-to-birth interval** |
| --- | --- | --- | --- | --- | --- | --- | --- | --- |
| Dexiprom 1999 (1) | Singleton and multiple neonates of women with PPROM | 28 - 24 | 208 | No ACS | 8/102 (7.8) | REF | No difference in odds of chorioamnionitis between >24 h and No ACS. | None |
|  |  |  |  | <24 h | 1/28 (3.6) | Too few events |  |  |
|  |  |  |  | >24 h | 10/74 (13.5) | OR 1.84 (95% CI 0.69 – 4.90) |  |  |

**Supplementary Table S20. Summary of findings on antenatal corticosteroid administration-to-birth interval and chorioamnionitis (observational studies)**

| **Study** | **Population** | **GA (weeks)** | **N** | **Time intervals** | **Chorioamnionitis (n/N) %** | **Effect estimate** | **Narrative Summary^1^** | **Optimal ACS administration-to-birth interval** |
| --- | --- | --- | --- | --- | --- | --- | --- | --- |
| Barrett 1982 (9) | Women with singleton or multiple pregnancy with ruptured membranes and no chorioamnionitis, fetal distress or abrupt placentae | 26 - 34 | 93 | No ACS | 20/56 (35.7%) | REF | -. | None |
|  |  |  |  | <24 h | 0/2 (0) | Too few events |  |  |
|  |  |  |  | 1 - <2 d | 2/6 (33.3%) | Too few events |  |  |
|  |  |  |  | 2 - <3 d | 3/13 (23.1%) | Too few events |  |  |
|  |  |  |  | 3 - <4 d | 3/4 (75.0%) | Too few events |  |  |
|  |  |  |  | 4 - <6 d | 1/2 (50.0%) | Too few events |  |  |
|  |  |  |  | ≥ 6 d | 1/9 (11.1%) | Too few events |  |  |
| Battarbee 2020 (10) | Singleton neonates | 23 - 34 | 2259 | <2 d | 56/622 (9.0%) | REF | Intervals of 2 - <7 d, 7 - <14 d and ≥14 d associated with increased odds of chorioamnionitis compared to <2 days. | <2 d |
|  |  |  |  | 2 - <7 d | 140/821 (17.1%) | OR 2.08 (95% CI 1.49-2.89) ^§^ |  |  |
|  |  |  |  | 7 - <14 d | 88/401 (21.9%) | OR 2.84 (95% CI 1.98-4.08) ^§^ |  |  |
|  |  |  |  | ≥14 d | 67/415 (16.1%) | OR 1.95 (95% CI 1.33-2.84) ^§^ |  |  |
| Chawla 2010 (12) | Singleton neonates with birthweight 401-1000g and without congenital anomalies | ≤28 | 169 | No ACS | 7/27 (25.9%) | REF | Intervals of 24 h – 7 d and >7 d associated with increased odds of chorioamnionitis compared to no ACS. | None |
|  |  |  |  | <24 h | 21/48 (43.8%) | OR 2.22 (95% CI 0.79-6.24) ^§^ |  |  |
|  |  |  |  | 24 h - 7 d | 27/53 (50.9%) | OR 2.97 (95% CI 1.07-8.19) ^§^ |  |  |
|  |  |  |  | >7 d | 22/41 (53.7%) | OR 3.31 (95% CI 1.15-9.52) ^§^ |  |  |
| Karmoker 2020 (17) | Singleton neonates without congenital anomalies | 24 - 34 | 200 | 48 h - <7d | 7/140 (5.0%) | REF | - | None |
|  |  |  |  | 7-14 d | 3/60 (5.0%) | Too few events |  |  |
| Kuk 2013 (18) | Twin neonates | 23 - 34 | 468 | No ACS | 1/61 (1.6%) | *Clinical chorioamnionitis*  REF | No difference in odds of histological chorioamnionitis at any time interval compared to no ACS. | None |
|  |  |  |  | <2 d | 4/83 (4.8%) | Too few events |  |  |
|  |  |  |  | 2-7 d | 2/57 (3.5%) | Too few events |  |  |
|  |  |  |  | >7 d | 3/33 (9.1%) | Too few events |  |  |
|  |  |  |  | No ACS | 11/52 (21.2%) | *Histological chorioamnionitis*  REF |  |  |
|  |  |  |  | <2 d | 22/84 (26.2%) | OR 1.65 (95% CI 0.73-3.71) ^§^ |  |  |
|  |  |  |  | 2-7 d | 15/55 (27.3%) | OR 1.74 (95% CI 0.72-4.2) ^§^ |  |  |
|  |  |  |  | >7 d | 11/31 (35.4%) | OR 1.7 (95% CI 0.66-4.39) ^§^ |  |  |
| Liebowitz 2016 (20) | Singleton and multiple neonates without major anomalies admitted to the NICU. | <28 | 667 | No ACS or < 6 h | 26/183 (14.2%) | REF | Intervals ≥24 h, < 10 d and ≥10 d associated with increased odds of chorioamnionitis compared to no ACS or <6 h duration. | No ACS or <6 h. |
|  |  |  |  | ≥24 h | 112/429 (26.1%) | OR 2.13 (95% CI 1.34- 3.41) ^§^ |  |  |
|  |  |  |  | <10 d | 78/312 (25.0%) | OR 2.01 (95% CI 1.24- 3.28) ^§^ |  |  |
|  |  |  |  | ≥10 d | 23/80 (38.8%) | OR 2.44 (95% CI 1.29-4.61) ^§^ |  |  |
| Nair 2009 (24) | Singleton neonates without congenital anomalies admitted to NICU | 24 - 28 | 163 | No ACS | 10/27 (37.0%) | REF | No difference in odds of chorioamnionitis at <24 h compared to no ACS. | None |
|  |  |  |  | <24 h | 16/29 (55.2%) | OR 1.18 (95% CI 0.43- 3.24) ^§^ |  |  |
| Ring 2007 (58) | Singleton neonates without congenital anomalies | 26 - 34 | 357 | 2-14 d | 11/259 (4.2%) | REF | - | None |
|  |  |  |  | >14 d | 4/98 (4.1%) | Too few events |  |  |
| Sehdev 2004 (31) | Singleton neonates with birthweight 500-1500g born to women admitted for preterm labour, PROM, or indicated for labour (CAM, non-reassuring fetal testing) | <28 | 325 | <24 h | 19/44 (43.2%) | REF | No difference in odds of chorioamnionitis at any time interval compared to <24 h. | None |
|  |  |  |  | 1-2 d | 28/95 (29.5%) | OR 0.55 (95% CI 0.26-1.15) ^§^ |  |  |
|  |  |  |  | 2-7 d | 47/106 (44.3%) | OR 1.05 (95% CI 0.52-2.13) ^§^ |  |  |
|  |  |  |  | >7 d | 35/80 (43.8%) | OR 1.02 (95% CI 0.49-2.15) ^§^ |  |  |
|  |  |  |  | <24 h | 5/119 (4.2%) | REF |  |  |
|  |  |  |  | <24 h or >7 d | 20/249 (8.0%) | OR 1.99 (95% CI 0.73-5.44) ^§^ |  |  |
|  |  |  |  | 24 h – 7 d | 40/420 (9.5%) | OR 2.40 (95% CI 0.93-6.22) ^§^ |  |  |
| Tomotaki 2021 (51) | VLBW neonates | <30 | 115 | No ACS or <24 h | 8/36 (22.2%) | REF | No difference in odds of chorioamnionitis at any time interval compared to no ACS or <24 h. | None |
|  |  |  |  | 1-7 d | 16/41 (39.0%) | OR 2.24 (95% CI 0.82 – 6.12)^§^ |  |  |
|  |  |  |  | >8 d | 14/38 (36.8%) | OR 2.04 (95% CI 0.73 – 5.69)^§^ |  |  |
| Vermillion 2001 (52) | Women with singleton pregnancy, intact membranes and no fetal anomalies | 28 - 34 | 216 | 1-2 d | 3/97 (3.0%) | REF | - | None |
|  |  |  |  | 3-7 d | 2/78 (2.6%) | Too few events |  |  |
|  |  |  |  | 8-14 d | 2/41 (4.9%) | Too few events |  |  |
| Wong 2014 (34) | Singleton or multiple neonates without congenital anomalies admitted to NICU | <29 | 2549 | No ACS | 31/176 (17.6%) | REF | Interval of >7 d associated with increased odds of chorioamnionitis compared to no ACS. | None |
|  |  |  |  | <24 h | 84/414 (20.3%) | OR 1.34 (95% CI 0.85-2.10) ^§^ |  |  |
|  |  |  |  | < - 7 d | 155/713 (21.7%) | OR 1.46 (95% CI 0.96-2.23) ^§^ |  |  |
|  |  |  |  | >7 d | 219/880 (24.9%) | OR 1.74 (95% CI 1.15-2.63) ^§^ |  |  |
| Yasuhi 2017 (55) | Women with singleton pregnancy with no fetal anomalies | 24 - 33 | 397 | <7 d | 4/83 (4.8%) | REF | - | None |
|  |  |  |  | 7-14 d | 1/14 (7.1%) | Too few events |  |  |
|  |  |  |  | >14 d | 1/20 (5.0%) | Too few events |  |  |

§ Crude odds ratios and 95% confidence intervals calculated by review authors using data provided in published paper. NR: Not reported. The brief narrative summary is based on available effect estimates, and the author’s conclusions for that study

**References**

1. Pattinson RC, Makin JD, Funk M, Delport SD, Macdonald AP, Norman K, et al. The use of dexamethasone in women with preterm premature rupture of membranes--a multicentre, double-blind, placebo-controlled, randomised trial. Dexiprom Study Group. South African medical journal. 1999;89(8):865‐70.

2. Who Action Trials C. Antenatal dexamethasone for late preterm birth: A multi-centre, two-arm, parallel, double-blind, placebo-controlled, randomized trial. EClinicalMedicine.44:101285.

3. Kosinska Kaczynska K, Szymusik I, Urban P, Zachara M, Wielgos M. Relation between time interval from antenatal corticosteroids administration to delivery and neonatal outcome in twins. Journal of Obstetrics & Gynaecology Research. 2016;42(6):625-31.

4. Hurrell A, Busuulwa P, Webster L, Duhig K, Seed PT, Chappell LC, et al. Optimising timing of steroid administration in preterm pre-eclampsia. Pregnancy Hypertension.30:177-80.

5. McEvoy C, Schilling D, Spitale P, Peters D, O'Malley J, Durand M. Decreased respiratory compliance in infants less than or equal to 32 weeks' gestation, delivered more than 7 days after antenatal steroid therapy. Pediatrics. 2008;121(5):e1032-8.

6. Nagy G, Kardos Z, Mahunka M. [Relationship of time interval between steroid therapy and labor to the incidence of respiratory distress syndrome]. Zusammenhang der zwischen Steroidtherapie und Geburt vergangenen Zeit mit der Haufigkeit des Respirations-Distress-Syndroms. 1978;100(13):833-41.

7. Kari MA, Hallman M, Eronen M, Teramo K, Virtanen M, Koivisto M, et al. Prenatal dexamethasone treatment in conjunction with rescue therapy of human surfactant: a randomized placebo-controlled multicenter study. Pediatrics. 1994;93(5):730‐6.

8. Arulalan MJ, Dorairajan G, Mondal N, Chinnakali P. Comparison of respiratory distress syndrome amongst preterm twins (28-34 Weeks) born within and after two weeks of completion of single antenatal corticosteroid course: A bidirectional cohort study. Journal of Mother and Child. 2021;25(4):260-8.

9. Barrett JM, Boehm FH. Comparison of aggressive and conservative management of premature rupture of fetal membranes. Am J Obstet Gynecol. 1982;144(1):12-6.

10. Battarbee AN, Manuck TA, Esplin MS, Varner MW, Ros ST, Biggio J, et al. Optimal timing of antenatal corticosteroid administration and preterm neonatal and early childhood outcomes. American Journal of Obstetrics and Gynecology MFM. 2020;2(1):100077.

11. Biedermann R, Schleussner E, Lauten A, Heimann Y, Lehmann T, Proquitte H, et al. Inadequate Timing Limits the Benefit of Antenatal Corticosteroids on Neonatal Outcome: Retrospective Analysis of a High-Risk Cohort of Preterm Infants in a Tertiary Center in Germany. Geburtshilfe und Frauenheilkunde. 2022;82(3):317-25.

12. Chawla S, Natarajan G, Rane S, Thomas R, Cortez J, Lua J. Outcomes of extremely low birth weight infants with varying doses and intervals of antenatal steroid exposure. Journal of perinatal medicine. 2010;38(4):419-23.

13. Fortmann I, Mertens L, Boeckel H, Gruttner B, Humberg A, Astiz M, et al. A Timely Administration of Antenatal Steroids Is Highly Protective Against Intraventricular Hemorrhage: An Observational Multicenter Cohort Study of Very Low Birth Weight Infants. Frontiers in Pediatrics. 2022;10 (no pagination).

14. Fuller KP, DeGroff S, Borgida AF. Neonatal outcomes based on antenatal corticosteroid exposure time for infants delivered between 23 and 34 weeks gestation. Clinical and experimental obstetrics & gynecology. 2017;44(2):247-51.

15. Gaur K, Ganguly B. Effect of single dose betamethasone administration in pregnancy on maternal and newborn parameters. Journal of Clinical and Diagnostic Research. 2017;11(5):FC15-FC8.

16. Haas DM, McCullough W, McNamara MF, Olsen C. The first 48 hours: Comparing 12-hour and 24-hour betamethasone dosing when preterm deliveries occur rapidly. The journal of maternal-fetal & neonatal medicine : the official journal of the European Association of Perinatal Medicine, the Federation of Asia and Oceania Perinatal Societies, the International Society of Perinatal Obstetricians. 2006;19(6):365-9.

17. Karmoker RK, Mirza TT, Hossain AK, Ali MA, Sarker K, Zaman K, et al. Influence of the Interval between Antenatal Corticosteroid Therapy and Delivery on the Incidence of Respiratory Distress Syndrome in Neonate. Mymensingh medical journal : MMJ. 2020;29(1):60-5.

18. Kuk J-Y, An J-J, Cha H-H, Choi S-J, Vargas JE, Oh S-y, et al. Optimal time interval between a single course of antenatal corticosteroids and delivery for reduction of respiratory distress syndrome in preterm twins. Am J Obstet Gynecol. 2013;209(3):256.e1-7.

19. Kyser KL, Morriss FH, Jr., Bell EF, Klein JM, Dagle JM. Improving survival of extremely preterm infants born between 22 and 25 weeks of gestation. Obstet Gynecol. 2012;119(4):795-800.

20. Liebowitz M, Clyman RI. Antenatal Betamethasone: A Prolonged Time Interval from Administration to Delivery Is Associated with an Increased Incidence of Severe Intraventricular Hemorrhage in Infants Born before 28 Weeks Gestation. The Journal of pediatrics. 2016;177:114-20.e1.

21. Li L, Li H, Jiang Y, Yu B, Wang X, Zhang W. The Relationship between Antenatal Corticosteroid Administration-to-Delivery Intervals and Neonatal Respiratory Distress Syndrome and Respiratory Support. Journal of Healthcare Engineering.2022:2310080.

22. Madarek EO, Najati N. The effect of glucocorticoid therapy in preventing early neonatal complications in preterm delivery. Journal of perinatal medicine. 2003;31(5):441-3.

23. Melamed N, Shah J, Soraisham A, Yoon EW, Lee SK, Shah PS, et al. Association Between Antenatal Corticosteroid Administration-to-Birth Interval and Outcomes of Preterm Neonates. Obstet Gynecol. 2015;125(6):1377-84.

24. Nair GV, Omar SA. Blood pressure support in extremely premature infants is affected by different courses of antenatal steroids. Acta paediatrica (Oslo, Norway : 1992). 2009;98(9):1437-43.

25. Norberg H, Kowalski J, Marsal K, Norman M. Timing of antenatal corticosteroid administration and survival in extremely preterm infants: a national population-based cohort study. BJOG : an international journal of obstetrics and gynaecology. 2017;124(10):1567-74.

26. Norman M, Piedvache A, Børch K, Huusom LD, Bonamy A-KE, Howell EA, et al. Association of Short Antenatal Corticosteroid Administration-to-Birth Intervals With Survival and Morbidity Among Very Preterm Infants: Results From the EPICE Cohort. JAMA Pediatrics. 2017;171(7):678-86.

27. Palas D, Ehlinger V, Alberge C, Truffert P, Kayem G, Goffinet F, et al. Efficacy of antenatal corticosteroids in preterm twins: the EPIPAGE-2 cohort study. BJOG : an international journal of obstetrics and gynaecology. 2018;125(9):1164-70.

28. Peaceman AM, Bajaj K, Kumar P, Grobman WA. The interval between a single course of antenatal steroids and delivery and its association with neonatal outcomes. Am J Obstet Gynecol. 2005;193(3 Pt 2):1165-9.

29. Ryu YH, Oh S, Sohn J, Lee J. The Associations between Antenatal Corticosteroids and In-Hospital Outcomes of Preterm Singleton Appropriate for Gestational Age Neonates according to the Presence of Maternal Histologic Chorioamnionitis. Neonatology. 2019;116(4):369-75.

30. Schmidt B, Seshia M, Shankaran S, Mildenhall L, Tyson J, Lui K, et al. Effects of prophylactic indomethacin in extremely low-birth-weight infants with and without adequate exposure to antenatal corticosteroids. Archives of pediatrics & adolescent medicine. 2011;165(7):642-6.

31. Sehdev HM, Abbasi S, Robertson P, Fisher L, Marchiano DA, Gerdes JS, et al. The effects of the time interval from antenatal corticosteroid exposure to delivery on neonatal outcome of very low birth weight infants. Am J Obstet Gynecol. 2004;191(4):1409-13.

32. Sen S, Reghu A, Ferguson SD. Efficacy of a single dose of antenatal steroid in surfactant-treated babies under 31 weeks' gestation. The journal of maternal-fetal & neonatal medicine : the official journal of the European Association of Perinatal Medicine, the Federation of Asia and Oceania Perinatal Societies, the International Society of Perinatal Obstetricians. 2002;12(5):298-303.

33. Siegler Y, Justman N, Bachar G, Lauterbach R, Zipori Y, Khatib N, et al. Is there a benefit of antenatal corticosteroid when given < 48 h before delivery? Archives of Gynecology and Obstetrics. 2022;306(5):1463-8.

34. Wong D, Abdel-Latif M, Kent A, Network N. Antenatal steroid exposure and outcomes of very premature infants: a regional cohort study. Archives of disease in childhood Fetal and neonatal edition. 2014;99(1):F12-20.

35. Anonymous. Effect of antenatal dexamethasone administration on the prevention of respiratory distress syndrome. Am J Obstet Gynecol. 1981;141(3):276-87.

36. Block MF, Kling OR, Crosby WM. Antenatal glucocorticoid therapy for the prevention of respiratory distress syndrome in the premature infant. Obstet Gynecol. 1977;50(2):186-90.

37. Gamsu HR, Mullinger BM, Donnai P, Dash CH. Antenatal administration of betamethasone to prevent respiratory distress syndrome in preterm infants: report of a UK multicentre trial. British journal of obstetrics and gynaecology. 1989;96(4):401-10.

38. Liggins GC, Howie RN. A controlled trial of antepartum glucocorticoid treatment for prevention of the respiratory distress syndrome in premature infants. Pediatrics. 1972;50(4):515-25.

39. Luerti M, Lazzarin A, Corbella E, Zavattini G. An alternative to steroids for prevention of respiratory distress syndrome (RDS): multicenter controlled study to compare ambroxol and betamethasone. Journal of perinatal medicine. 1987;15(3):227‐38.

40. Schutte MF, Treffers PE, Koppe JG, Breur W. The influence of betamethasone and orciprenaline on the incidence of respiratory distress syndrome in the newborn after preterm labour. British journal of obstetrics and gynaecology. 1980;87(2):127-31.

41. Teramo K, Hallman M, Raivio KO. Maternal glucocorticoid in unplanned premature labor. Controlled study on the effects of betamethasone phosphate on the phospholipids of the gastric aspirate and on the adrenal cortical function of the newborn infant. Pediatr Res. 1980;14(4 Pt 1):326‐9.

42. Asl MM, Kashanian M, Shirzi AA. The incidence of respiratory distress syndrome in preterm infants born during the first 24 hours of dexamethasone administration to mothers. Journal of Iran University of Medical Sciences. 2005;12(45):180-.

43. Caspi E, Schreyer P, Weinraub Z, Reif R, Levi I, Mundel G. Prevention of the respiratory distress syndrome in premature infants by antepartum glucocorticoid therapy. British journal of obstetrics and gynaecology. 1976;83(3):187-93.

44. Ferguson S, Allen VM, Craig C, Allen AC, Dodds L. Timing of indicated delivery after antenatal steroids in preterm pregnancies with severe hypertension. Hypertension in pregnancy. 2009;28(1):63-75.

45. Frandberg J, Sandblom J, Bruschettini M, Marsal K, Kristensen K. Antenatal corticosteroids: a retrospective cohort study on timing, indications and neonatal outcome. Acta obstetricia et gynecologica Scandinavica. 2018;97(5):591-7.

46. Gulersen M, Gyamfi-Bannerman C, Greenman M, Lenchner E, Rochelson B, Bornstein E. Time interval from late preterm antenatal corticosteroid administration to delivery and the impact on neonatal outcomes. Am J Obstet Gynecol MFM. 2021;3(5):100426.

47. Guruvare S, Basu B, Rai L, Hebbar S, Adiga P, Lewis L. Relationship of time interval between antenatal corticosteroid administrations to delivery with respiratory distress in preterm newborns. International Journal of Infertility and Fetal Medicine. 2015;6(3):128-32.

48. Janssen O, Ratner V, Lin J, Fox N, Green R. Respiratory and glycemic control outcomes of late preterm infants after antenatal corticosteroid exposure. Journal of Perinatology. 2021;41(11):2607-13.

49. Lau HCQ, Tung JSZ, Wong TTC, Tan PL, Tagore S. Timing of antenatal steroids exposure and its effects on neonates. Archives of gynecology and obstetrics. 2017;296(6):1091-6.

50. Sekhavat L, Firouzabadi RD, Karbasi SA. Comparison of interval duration between single course antenatal corticosteroid administration and delivery on neonatal outcomes. Journal of the Turkish German Gynecology Association. 2011;12(2):86-9.

51. Tomotaki S, Iwanaga K, Hanaoka S, Tomotaki H, Matsukura T, Niwa F, et al. Antenatal Glucocorticoids Reduce the Incidence of Refractory Hypotension in Low Birthweight Infants during the Early Neonatal Period, but Do Not Affect It beyond This Time. American journal of perinatology. 2021;38(10):1057-61.

52. Vermillion ST, Soper DE, Newman RB. Is betamethasone effective longer than 7 days after treatment? Obstet Gynecol. 2001;97(4):491-3.

53. Waters TP, Mercer B. Impact of timing of antenatal corticosteroid exposure on neonatal outcomes. The journal of maternal-fetal & neonatal medicine : the official journal of the European Association of Perinatal Medicine, the Federation of Asia and Oceania Perinatal Societies, the International Society of Perinatal Obstetricians. 2009;22(4):311-4.

54. Wilms FF, Vis JY, Pattinaja DAPM, Kuin RA, Stam MC, Reuvers JM, et al. Relationship between the time interval from antenatal corticosteroid administration until preterm birth and the occurrence of respiratory morbidity. Am J Obstet Gynecol. 2011;205(1):49.e1-7.

55. Yasuhi I, Myoga M, Suga S, Sugimi S, Umezaki Y, Fukuda M, et al. Influence of the interval between antenatal corticosteroid therapy and delivery on respiratory distress syndrome. J Obstet Gynaecol Res. 2017;43(3):486-91.

56. di Pasquo E, Saccone G, Angeli L, Dall'Asta A, Borghi E, Fieni S, et al. Determinants of neonatal hypoglycemia after antenatal administration of corticosteroids (ACS) for lung maturation: Data from two referral centers and review of the literature. Early human development. 2020;143:104984.

57. Dzidek S, Jasiak H, Bednarek-Jedrzejek M, Tousty P, Fraszczyk-Tousty M, Kwiatkowska E, et al. The temporal link between prenatal steroid therapy and labor. Polski merkuriusz lekarski : organ Polskiego Towarzystwa Lekarskiego. 2020;48(288):394-8.

58. Ring AM, Garland JS, Stafeil BR, Carr MH, Peckman GS, Pircon RA. The effect of a prolonged time interval between antenatal corticosteroid administration and delivery on outcomes in preterm neonates: a cohort study. Am J Obstet Gynecol. 2007;196(5):457.e1-6.
