# Supplementary material for: Effect of antenatal corticosteroid administration-to-birth interval on maternal and newborn outcomes: a systematic review
Source: eClinicalMedicine. 2023 Mar 24;58:101916. doi: 10.1016/j.eclinm.2023.101916 (PMC10050784; doi:10.1016/j.eclinm.2023.101916)
Supplement: Appendix S2 [file mmc4.docx]

# Supplemental data 2: Systematic review of antenatal corticosteroid administration to birth interval in preterm infants

## **Search Methodology**

Five databases were searched on 26 April 2021. The search was limited to human studies. The search strategy was refined with the assistance of an information specialist until the results retrieved appropriately reflected the scope of the project. The final Medline search was amended to run between Cochrane and Embase. CINAHL Complete and Global Index Medicus we utilized a simplified search strategy to attain appropriate/acceptable reference numbers.

The databases searched included:

1. Ovid MEDLINE(R) and Epub Ahead of Print, In-Process, In-Data-Review & Other Non-Indexed Citations and Daily <1946 to April 23, 2021>
2. OvidSP Embase Classic+Embase <1947 to 2021 April 23>
3. Cochrane database, complete
4. Global Index Medicus
5. EBSCO CINAHL Complete, complete

## **Search Results**

| Database | Endnote import order | Number of references before deduplication | Number of references after duplications removed (by Endnote) |
| --- | --- | --- | --- |
| OvidSP Medline | 1 | 2460 | 2460 |
| OvidSP Embase | 2 | 4719 | 6411 |
| Global Index Medicus | 3 | 27 | 6466 |
| CINAHL Complete | 4 | 342 | 6756 |
| Cochrane | 5 | 823 | 7371 |
| Total |  | 8371 | 7371 |

*Note: 2 studies identified by searching reference lists by hand and added to title and abstract screening.

## **Search Strategies**

### 3.1 MEDLINE

| Database | Medline |
| --- | --- |
| Database Platform | OvidSP |
| Dates of database coverage | 1946 to April 23, 2021 |
| Date searched | 26/04/2021 |
| Searched by | LA |
| Number of hits | 2460 |

Ovid MEDLINE(R) and Epub Ahead of Print, In-Process, In-Data-Review & Other Non-Indexed Citations, Daily and Versions(R) <1946 to April 23, 2021>

1 exp Pregnancy/ 914884

2 exp Prenatal Care/ 28959

3 exp Perinatal Care/ 10643

4 exp Obstetric Labor, Premature/ 27722

5 exp Pregnancy Complications/ 436690

6 exp Infant, Newborn/ 621675

7 (pregnan$ or preterm or prematur$ or birth or labo?r or deliver$).ti,ab,kf. 1550047

8 or/1-7 2297918

9 Adrenal Cortex Hormones/ 65860

10 exp Glucocorticoids/ 196004

11 exp Betamethasone/ 7498

12 exp Dexamethasone/ 52369

13 exp Duration of Therapy/ or exp Time Factors/ 1206197

14 (or/9-12) and 13 16410

15 ((antenatal corticosteroid$ or antenatal steroid$ or glucocorticoid$ or betamethasone or dexamethasone or anc or acs or ans) adj10 (duration or course$ or interval$ or timing or time or day$ or hour$)).ti,ab,kf. 18784

16 or/14-15 33985

17 exp Epidemiologic Studies/ 2648675

18 exp Comparative Study/ 1887950

19 exp Clinical Trial/ 889115

20 (trial or study or cohort or prospective or retrospective or compared or association$ or group$ or control$).ti,ab,kf. 13576914

21 or/17-20 14689705

22 8 and 16 and 21 3488

23 exp animals/ not humans.sh. 4816310

24 22 not 23 2460

### 3.2 EMBASE

| Database | Embase Classic + Embase |
| --- | --- |
| Database Platform | OvidSP |
| Dates of database coverage | 1947 to 2021 Week 16 |
| Date searched | 26/04/2021 |
| Searched by | LA |
| Number of hits | 4719 |

Embase Classic+Embase <1947 to 2021 Week 16>

1 exp pregnancy/ 834708

2 exp prenatal care/ 164472

3 exp perinatal care/ 63954

4 exp premature labor/ 50537

5 exp pregnancy complication/ 153548

6 exp infant/ 1210447

7 exp newborn/ 641703

8 (pregnan$ or preterm or prematur$ or birth or labo?r or deliver$).ti,ab,kw. 2120140

9 1 or 2 or 3 or 4 or 5 or 6 or 7 or 8 3315022

10 exp glucocorticoid/ 821034

11 exp betamethasone/ 18888

12 exp dexamethasone/ 163518

13 exp treatment duration/ or exp time factor/ 287894

14 10 or 11 or 12 821034

15 13 and 14 27562

16 ((antenatal corticosteroid$ or antenatal steroid* or glucocorticoid$ or betamethasone or dexamethasone or anc or acs or ans) adj10 (duration or course$ or interval$ or timing or time or day$ or hour$)).ti,ab,kw. 31408

17 15 or 16 58022

18 exp controlled study/ 8416216

19 exp clinical study/ 10760720

20 exp cohort analysis/ 702482

21 (trial or study or cohort or prospective or retrospective or compared or association$ or group$ or control$).ti,ab,kw. 18400616

22 18 or 19 or 20 or 21 23923961

23 9 and 17 and 22 6475

24 limit 23 to human 4879

25 limit 24 to abstracts 4719

### 3.3 GLOBAL INDEX MEDICUS

| Database | Global Index Medicus |
| --- | --- |
| Database Platform | Not applicable |
| Dates of database coverage | Not specified (dates not restricted) |
| Date searched | 26/04/2021 |
| Searched by | LA |
| Number of hits | 27 |

tw:((tw:(pregnan* OR preterm OR prematur* OR birth OR labor OR labour OR deliver*))

AND (tw:(glucocorticoid* OR steroid* OR corticosteroid* OR betamethasone OR dexamethasone))

AND (tw:(duration)))

AND ( mj:("Humans")

### 3.4 CINAHL

| Database | CINAHL Complete |
| --- | --- |
| Database Platform | EBSCOhost |
| Dates of database coverage | 1991 to 2021 |
| Date searched | 26/04/2021 |
| Searched by | LA |
| Number of hits | 342 |

( pregnan$ or preterm or prematur$ or birth or labo?r or deliver$ )

AND ( antenatal corticosteroid$ or antenatal steroid* or glucocorticoid$ or betamethasone or dexamethasone or ans or anc or acs)

AND ( duration or course$ or interval$ or timing or time or day$ or hour$ )

AND ( trial or study or cohort or prospective or retrospective or compared or association$ or group$ or control$ )

MEDLINE excluded

### 3.5 COCHRANE

| Database | Cochrane |
| --- | --- |
| Database Platform | Cochrane Library (Protocols, Reviews, Trials) |
| Dates of database coverage | Not specified (dates not restricted) |
| Date searched | 26/04/2021 |
| Searched by | LA |
| Number of hits | 823 |

#1 MeSH descriptor: [Pregnancy] explode all trees 22394

#2 MeSH descriptor: [Prenatal Care] explode all trees 1529

#3 MeSH descriptor: [Perinatal Care] explode all trees 583

#4 MeSH descriptor: [Obstetric Labor, Premature] explode all trees 2211

#5 ("pregnanc$" OR "preterm" OR "prematur$" OR "labor" OR "labour" OR "deliver$"):ti,ab,kw 34976

#6 #1 or #2 or #3 or #4 or #5 51672

#7 MeSH descriptor: [Glucocorticoids] explode all trees 4601

#8 MeSH descriptor: [Betamethasone] explode all trees 1470

#9 MeSH descriptor: [Dexamethasone] explode all trees 4678

#10 (((antenatal corticosteroid$ or antenatal steroid$ or glucocorticoid$ or betamethasone or dexamethasone or anc or acs or ans) NEAR/10 (duration or course$ or interval$ or timing or time or day$ or hour$))):ti,ab,kw 6621

#11 #7 or #8 or #9 or #10 14695

#12 #11 AND #6 823

## **Updated Search Methodology**

Five databases were searched on 11^th^ November 2022. The search was limited to human studies. The search strategy was repeated from the original search and limited to 27^th^ April – 11^th^ November.

The databases searched included:

1. Ovid MEDLINE(R) and Epub Ahead of Print, In-Process, In-Data-Review & Other Non-Indexed Citations and Daily <1946 to April 23, 2021>
2. OvidSP Embase Classic+Embase <1947 to 2021 April 23>
3. Cochrane database, complete
4. Global Index Medicus
5. EBSCO CINAHL Complete, complete

## **Search Strategies**

### 3.1 MEDLINE

| Database | Medline |
| --- | --- |
| Database Platform | OvidSP |
| Dates of database coverage | 1946 to November 03, 2022 |
| Date searched | 05/11/22 |
| Searched by | AS |
| Number of hits | 367 |

Ovid MEDLINE(R) and Epub Ahead of Print, In-Process, In-Data-Review & Other Non-Indexed Citations, Daily and Versions(R) <2021 to Nov 5, 2022>

*Only able to limit to year*

1 exp Pregnancy/ 983059

2 exp Prenatal Care/ 31625

3 exp Perinatal Care/ 11443

4 exp Obstetric Labor, Premature/ 32199

5 exp Pregnancy Complications/ 465518

6 exp Infant, Newborn/ 661696

7 (pregnan$ or preterm or prematur$ or birth or labo?r or deliver$).ti,ab,kf. 1709895

8 or/1-7 2488784

9 Adrenal Cortex Hormones/ 69260

10 exp Glucocorticoids/ 204835

11 exp Betamethasone/ 7834

12 exp Dexamethasone/ 54968

13 exp Duration of Therapy/ or exp Time Factors/ 1229080

14 (or/9-12) and 13 16642

15 ((antenatal corticosteroid$ or antenatal steroid$ or glucocorticoid$ or betamethasone or dexamethasone or anc or acs or ans) adj10 (duration or course$ or interval$ or timing or time or day$ or hour$)).ti,ab,kf. 20529

16 or/14-15 35930

17 exp Epidemiologic Studies/ 3031084

18 exp Comparative Study/ 1911732

19 exp Clinical Trial/ 954586

20 (trial or study or cohort or prospective or retrospective or compared or association$ or group$ or control$).ti,ab,kf. 15045408

21 or/17-20 16184007

22 8 and 16 and 21 3774

23 exp animals/ not humans.sh. 5060250

24 22 not 23 2710

25 limit 24 to yr=”2021-Current” 367

### 3.2 EMBASE

| Database | Embase Classic + Embase |
| --- | --- |
| Database Platform | OvidSP |
| Dates of database coverage | 1947 to 2022 November 03 |
| Date searched | 05/11/22 |
| Searched by | AS |
| Number of hits | 822 |

Embase Classic+Embase <1947 to 2022 November 03>

1 exp pregnancy/ 883034

2 exp prenatal care/ 176629

3 exp perinatal care/ 68312

4 exp premature labor/ 55715

5 exp pregnancy complication/ 176679

6 exp infant/ 1271991

7 exp newborn/ 673449

8 (pregnan$ or preterm or prematur$ or birth or labo?r or deliver$).ti,ab,kw. 2289007

9 1 or 2 or 3 or 4 or 5 or 6 or 7 or 8 3536208

10 exp glucocorticoid/ 883076

11 exp betamethasone/ 20225

12 exp dexamethasone/ 182042

13 exp treatment duration/ or exp time factor/ 324490

14 10 or 11 or 12 883076

15 13 and 14 31373

16 ((antenatal corticosteroid$ or antenatal steroid* or glucocorticoid$ or betamethasone or dexamethasone or anc or acs or ans) adj10 (duration or course$ or interval$ or timing or time or day$ or hour$)).ti,ab,kw. 34849

17 15 or 16 65091

18 exp controlled study/ 9380523

19 exp clinical study/ 11704604

20 exp cohort analysis/ 915769

21 (trial or study or cohort or prospective or retrospective or compared or association$ or group$ or control$).ti,ab,kw. 20008113

22 18 or 19 or 20 or 21 25789230

23 9 and 17 and 22 7178

24 limit 23 to human 5570

25 limit 24 to abstracts 5392

26 limit 25 to yr=”2021 – Current” 822

### *3.3 GLOBAL INDEX MEDICUS*

| Database | Global Index Medicus |
| --- | --- |
| Database Platform | Not applicable |
| Dates of database coverage | Not specified (dates not restricted) |
| Date searched | 05/11/22 |
| Searched by | AS |
| Number of hits | 2 |

tw:((tw:(pregnan* OR preterm OR prematur* OR birth OR labor OR labour OR deliver*))

AND (tw:(glucocorticoid* OR steroid* OR corticosteroid* OR betamethasone OR dexamethasone))

AND (tw:(duration)))

AND ( mj:("Humans")

### 3.4 CINAHL

| Database | CINAHL Complete |
| --- | --- |
| Database Platform | EBSCOhost |
| Dates of database coverage | 1991 to 2022 |
| Date searched | 05/11/22 |
| Searched by | AS |
| Number of hits | 114 |

( pregnan$ or preterm or prematur$ or birth or labo?r or deliver$ )

AND ( antenatal corticosteroid$ or antenatal steroid* or glucocorticoid$ or betamethasone or dexamethasone or ans or anc or acs)

AND ( duration or course$ or interval$ or timing or time or day$ or hour$ )

AND ( trial or study or cohort or prospective or retrospective or compared or association$ or group$ or control$ )

MEDLINE excluded

Limited to start month April start year 2021 – end month November end year 2022

### 3.5 COCHRANE

| Database | Cochrane |
| --- | --- |
| Database Platform | Cochrane Library (Protocols, Reviews, Trials) |
| Dates of database coverage | Not specified (dates not restricted) |
| Date searched | 05/11/22 |
| Searched by | AS |
| Number of hits | 72 |

#1 MeSH descriptor: [Pregnancy] explode all trees 24962

#2 MeSH descriptor: [Prenatal Care] explode all trees 1695

#3 MeSH descriptor: [Perinatal Care] explode all trees 624

#4 MeSH descriptor: [Obstetric Labor, Premature] explode all trees 2513

#5 ("pregnanc$" OR "preterm" OR "prematur$" OR "labor" OR "labour" OR "deliver$"):ti,ab,kw 39670

#6 #1 or #2 or #3 or #4 or #5 58448

#7 MeSH descriptor: [Glucocorticoids] explode all trees 4825

#8 MeSH descriptor: [Betamethasone] explode all trees 1548

#9 MeSH descriptor: [Dexamethasone] explode all trees 5149

#10 (((antenatal corticosteroid$ or antenatal steroid$ or glucocorticoid$ or betamethasone or dexamethasone or anc or acs or ans) NEAR/10 (duration or course$ or interval$ or timing or time or day$ or hour$))):ti,ab,kw 7447

#11 #7 or #8 or #9 or #10 16104

#12 #11 AND #6 909

Limit #12 to April 2021 – November 2022 72
