# Supplementary material for: Effect of antenatal corticosteroid administration-to-birth interval on maternal and newborn outcomes: a systematic review
Source: eClinicalMedicine. 2023 Mar 24;58:101916. doi: 10.1016/j.eclinm.2023.101916 (PMC10050784; doi:10.1016/j.eclinm.2023.101916)
Supplement: Figs. S1–S4 [file mmc5.docx]

**Supplemental Figure S1. Randomised controlled trials risk of bias assessment**

**
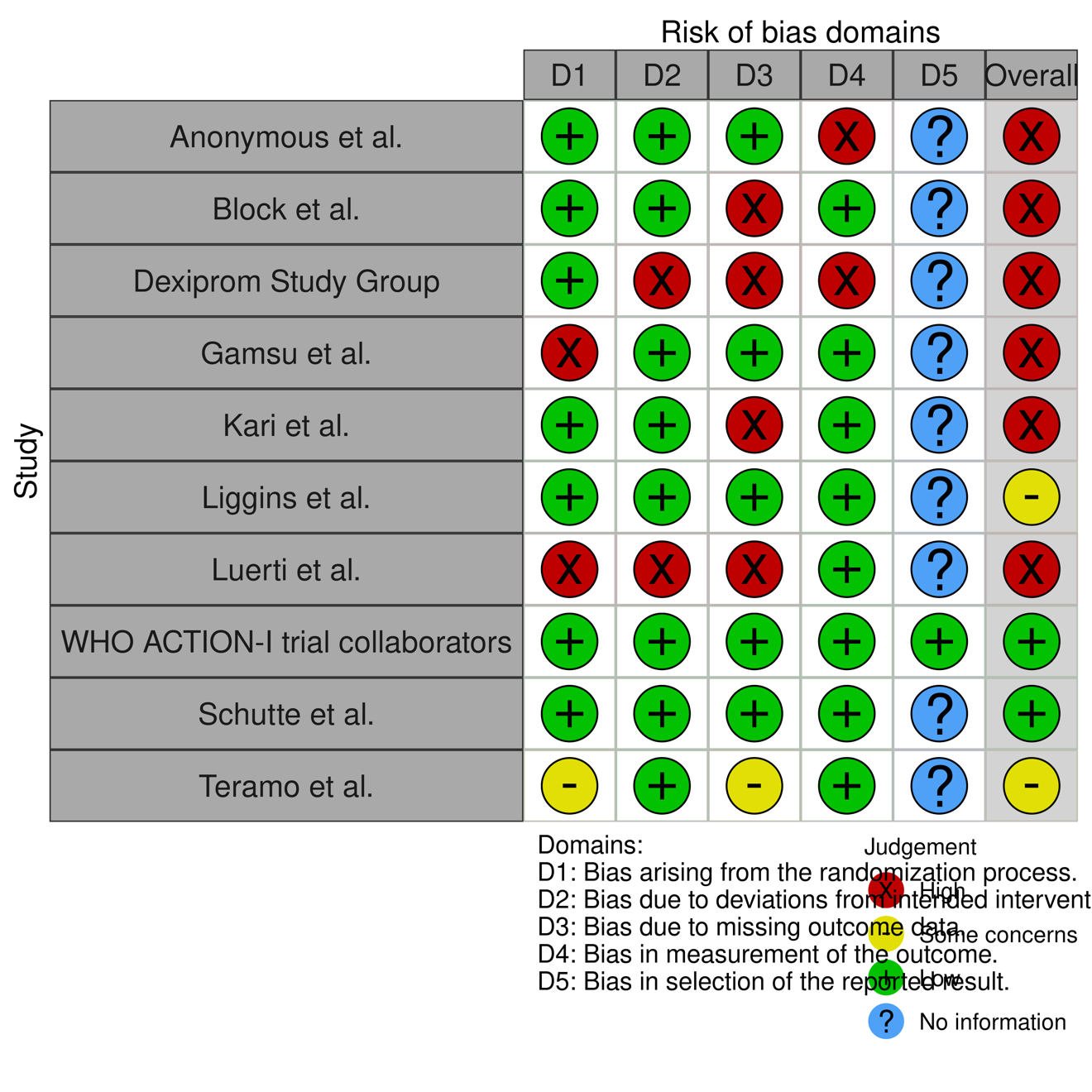
**

**Supplemental Figure S2. Randomised controlled trials: summary of risk of bias assessments**

**
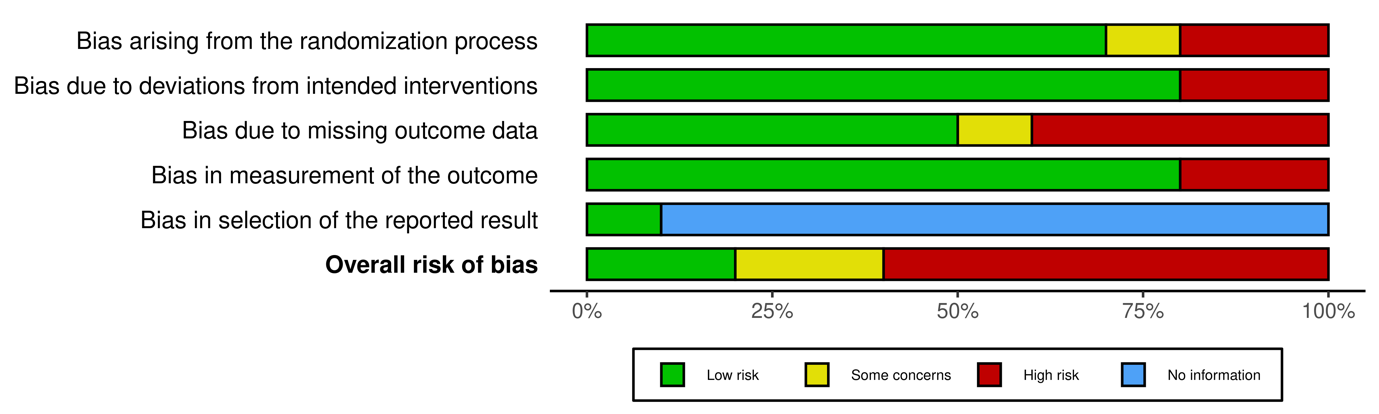
**

**Supplemental Figure S3. Risk of bias assessment for observational studies**

**
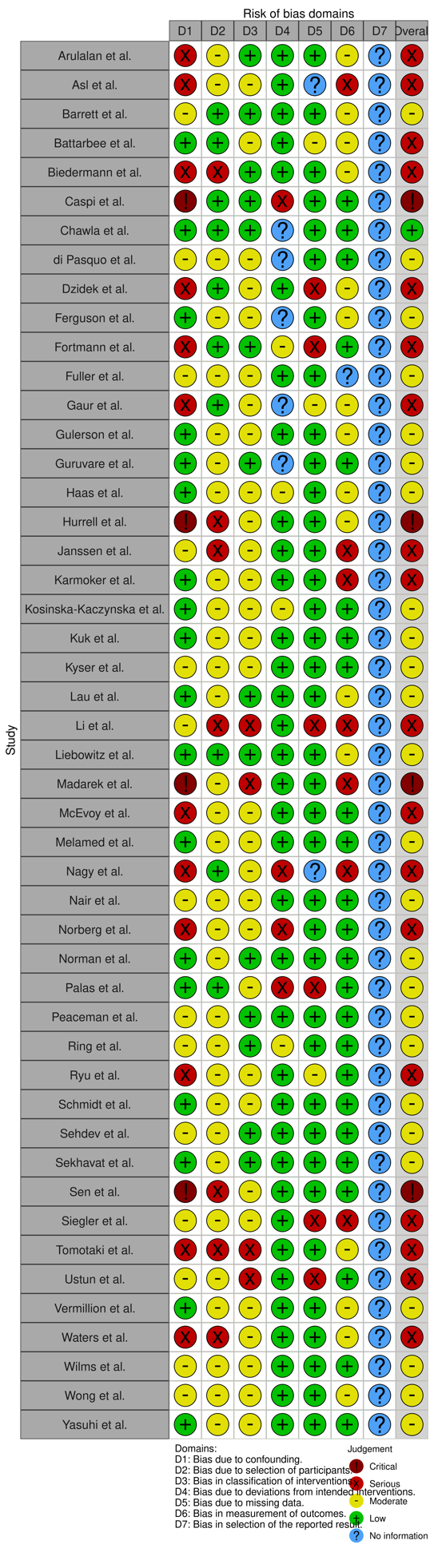
**

**Supplemental Figure S4. Risk of bias assessment summary: observational studies**

**
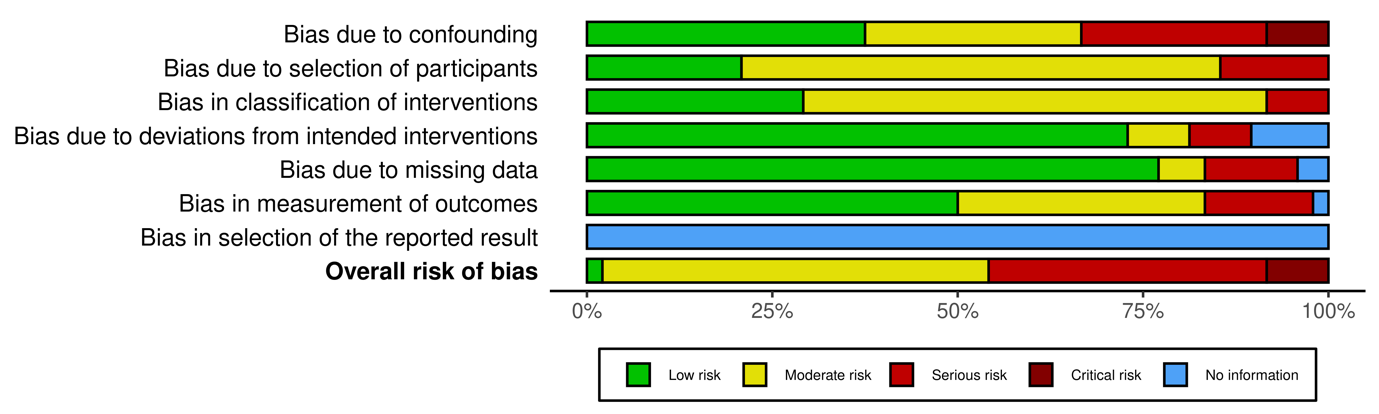
**
