# Supplementary material for: Effect of antenatal corticosteroid administration-to-birth interval on maternal and newborn outcomes: a systematic review
Source: eClinicalMedicine. 2023 Mar 24;58:101916. doi: 10.1016/j.eclinm.2023.101916 (PMC10050784; doi:10.1016/j.eclinm.2023.101916)
Supplement: Figs. S5–S9 [file mmc6.docx]

**Figure S5. Descriptive summary of reported intraventricular haemorrhage outcomes from observational studies**

A visual representation summarising odds ratio of intraventricular haemorrhage for different time intervals compared to “no antenatal corticosteroids” group (7 studies; 13 additional studies did not include a “no antenatal corticosteroids” group or had two few events). Green data points indicate a statistically significant decrease in odds ratio for intraventricular haemorrhage (i.e. upper bound of 95% CI was below 1). Black data points indicate the odds ratio of intraventricular haemorrhage was not significantly different (i.e. 95% CI included 1).

**
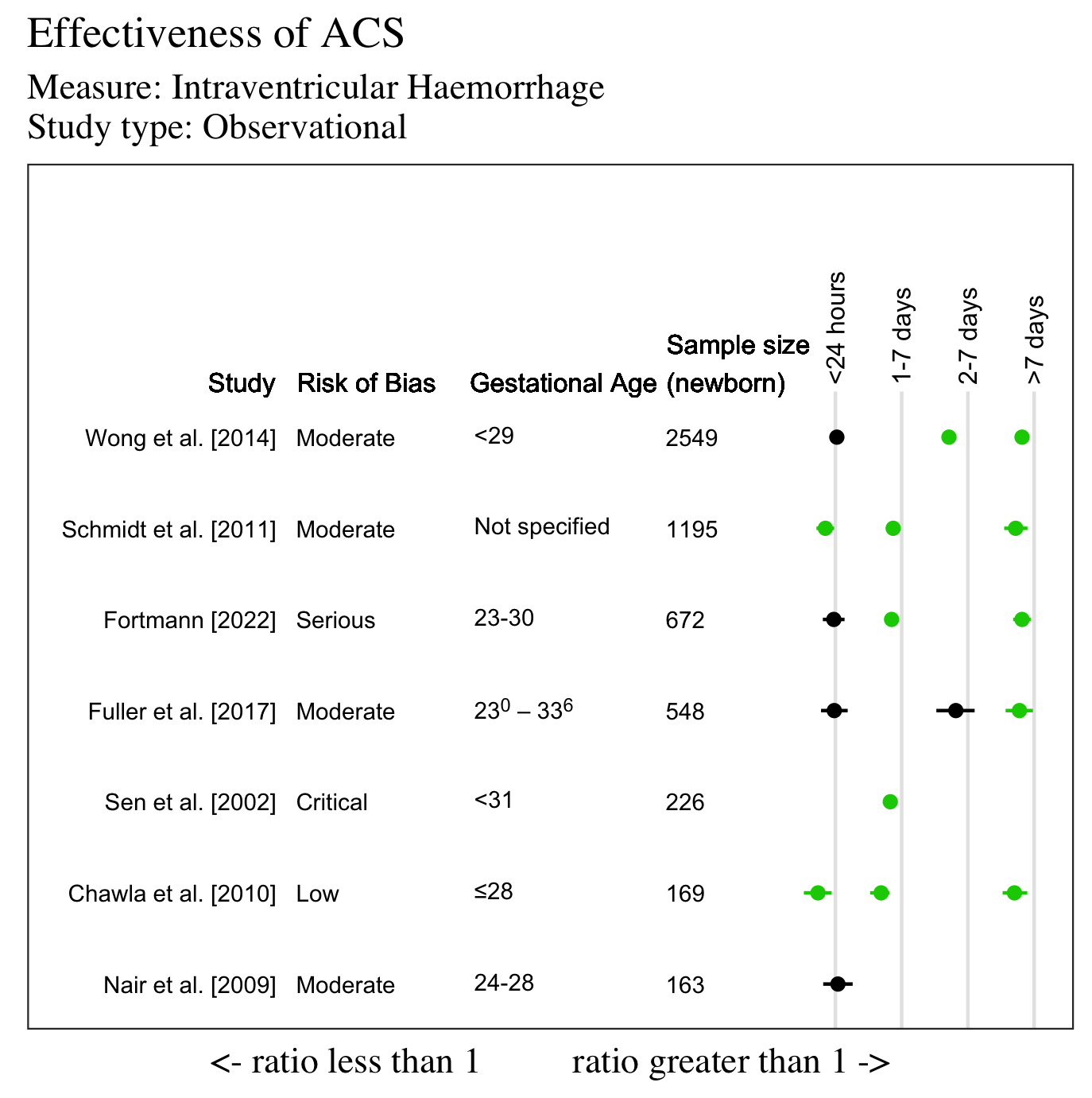
**

**Figure S6. Descriptive summary of reported necrotising enterocolitis outcomes from observational studies**

A visual representation summarising odds ratio of necrotising enterocolitis for different time intervals compared to “no antenatal corticosteroids” group (4 studies; 11 additional studies did not include a “no antenatal corticosteroids” group or had two few events). Red data points indicate a statistically significant increase in the odds of necrotising enterocolitis (i.e. lower bound was above 1). Black data points indicate the odds ratio of necrotising enterocolitis was not significantly different (i.e. 95% CI included 1).

**
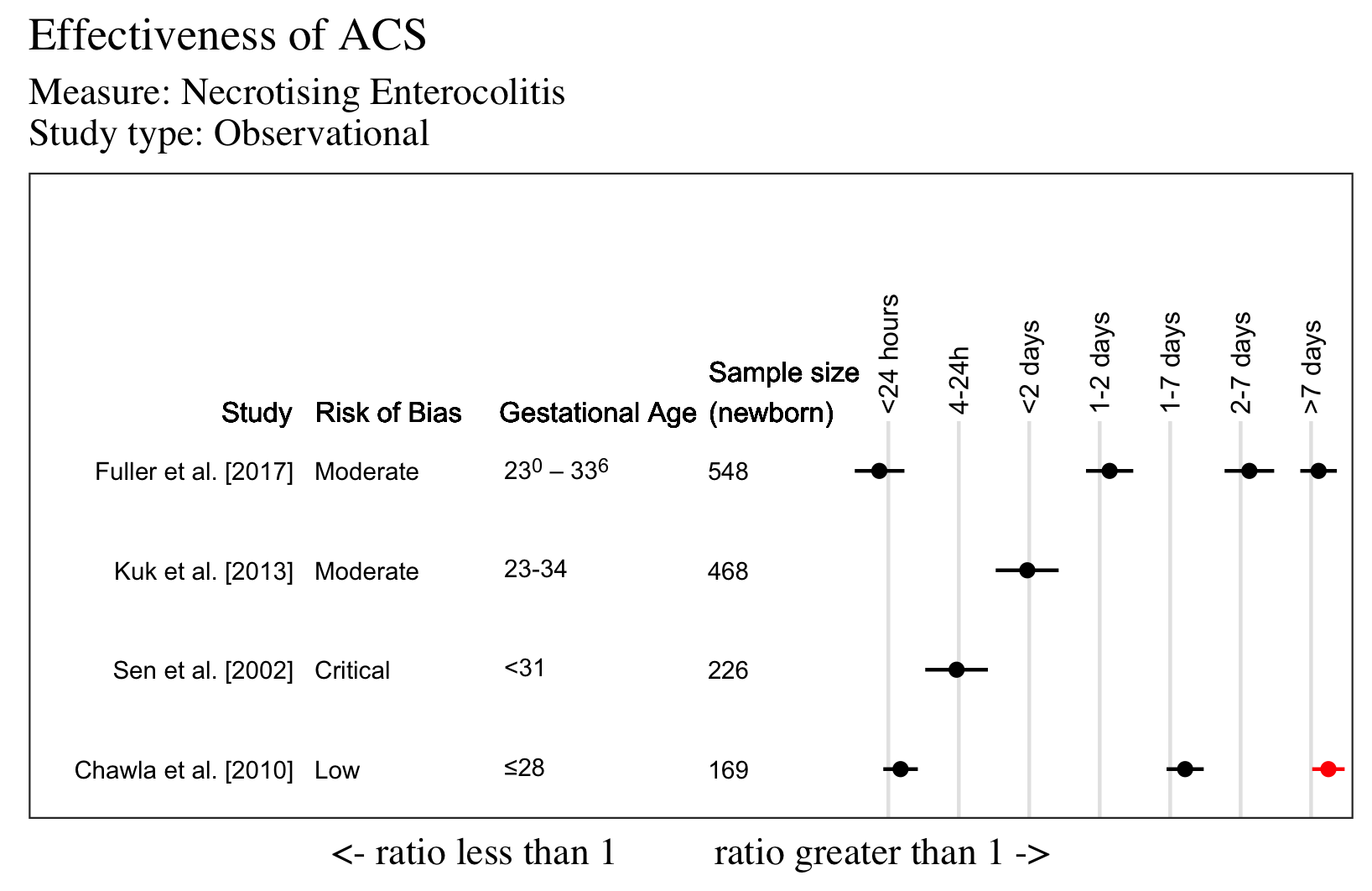
**

**Figure S7. Descriptive summary of reported bronchopulmonary dysplasia outcomes from observational studies**

A visual representation summarising odds ratio of bronchopulmonary dysplasia for different time intervals compared to “no antenatal corticosteroids” group (6 studies; 11 additional studies did not include a “no antenatal corticosteroids” group or had two few events). Green data points indicate a statistically significant decrease in odds ratio for bronchopulmonary dysplasia (i.e. upper bound of 95% CI was below 1). Red data points indicate a statistically significant increase in the odds of bronchopulmonary dysplasia (i.e. lower bound was above 1). Black data points indicate the odds ratio of bronchopulmonary dysplasia was not significantly different (i.e. 95% CI included 1).

**
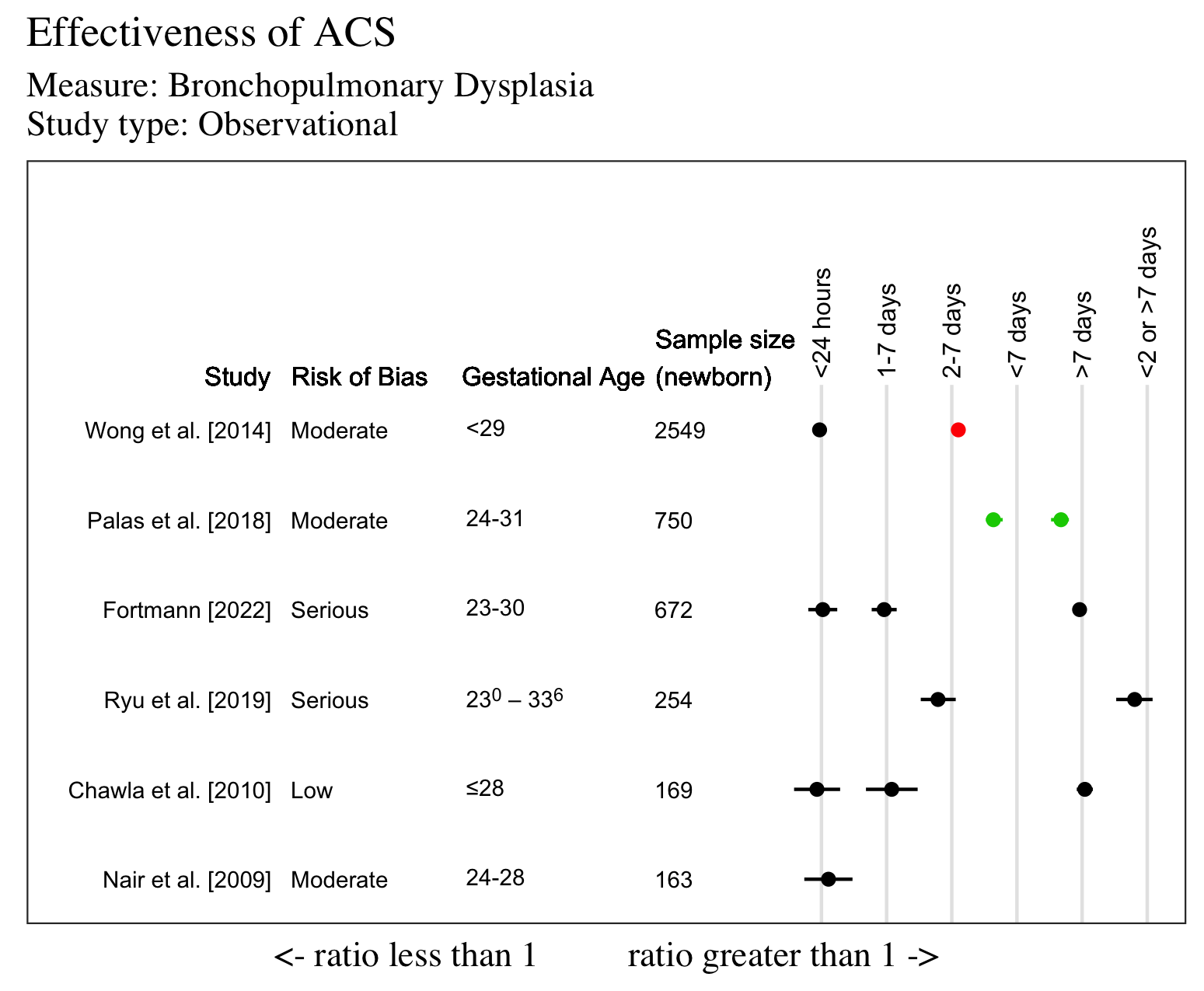
**

**Figure S8. Descriptive summary of reported neonatal sepsis outcomes from observational studies**

A visual representation summarising odds ratio of neonatal sepsis for different time intervals compared to “no antenatal corticosteroids” group (6 studies; 7 additional studies did not include a “no antenatal corticosteroids” group or had two few events). Red data points indicate a statistically significant increase in the odds of neonatal sepsis (i.e. lower bound was above 1). Black data points indicate the odds ratio of neonatal sepsis was not significantly different (i.e. 95% CI included 1).

**
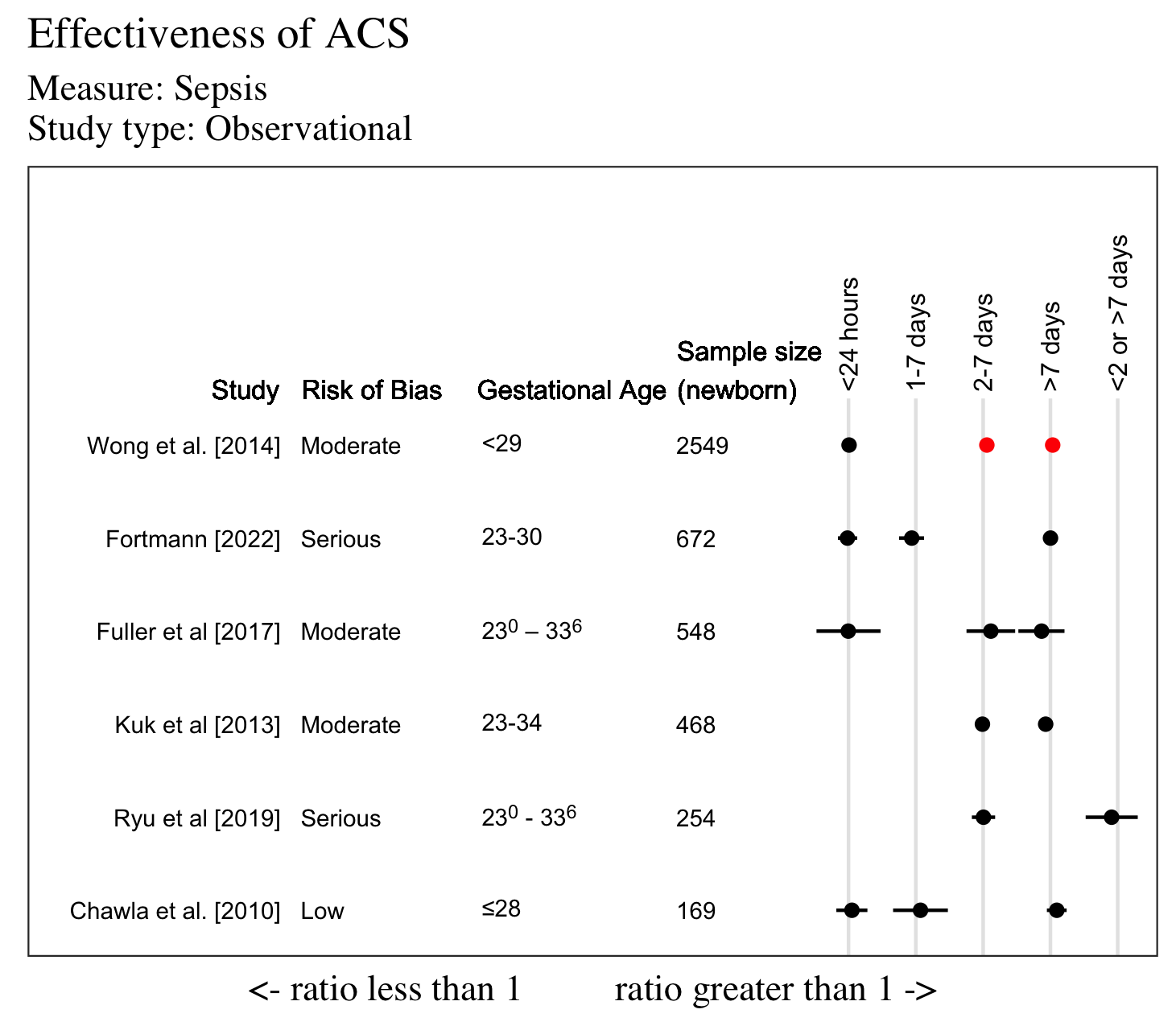
**

**Figure S9. Descriptive summary of reported chorioamnionitis outcomes from observational studies**

A visual representation summarising odds ratio of chorioamnionitis for different time intervals compared to “no antenatal corticosteroids” group (4 studies; 9 additional studies did not include a “no antenatal corticosteroids” group or had two few events). Red data points indicate a statistically significant increase in the odds of chorioamnionitis (i.e. lower bound was above 1). Black data points indicate the odds ratio of chorioamnionitis was not significantly different (i.e. 95% CI included 1).

**
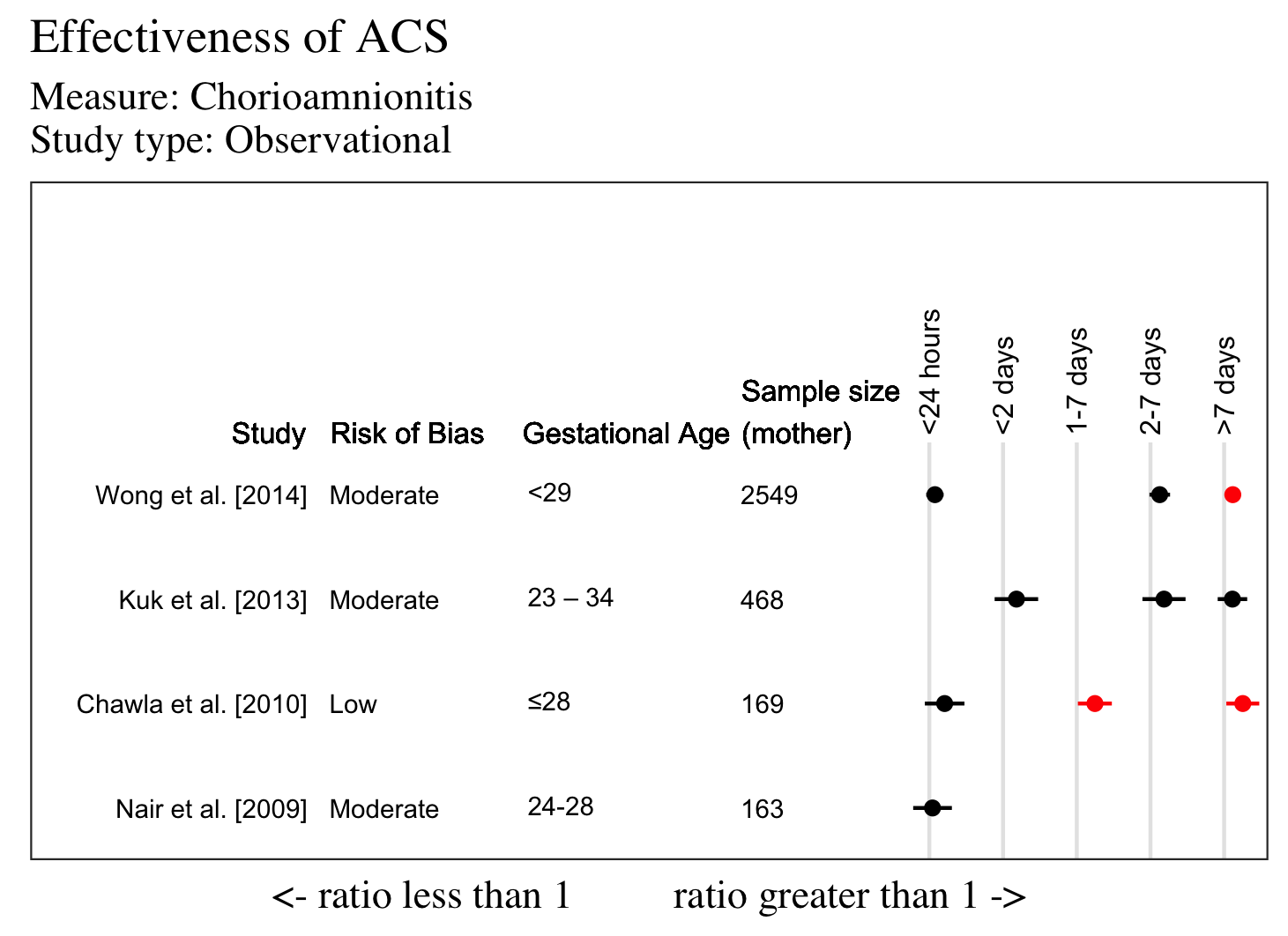
**
